# Supplementary material for: Deaths with COVID-19 and from all-causes following first-ever SARS-CoV-2 infection in individuals with preexisting mental disorders: A national cohort study from Czechia
Source: PLoS Med. 2024 Jul 15;21(7):e1004422. doi: 10.1371/journal.pmed.1004422 (PMC11285938; doi:10.1371/journal.pmed.1004422)
Supplement: S1 Methods — (DOCX) [file pmed.1004422.s001.docx]

Table of Contents

[Supplementary Methods 2](#_Toc167092816)

[Deviations from the Analytical Plan 2](#_Toc167092817)

[Exposure 2](#_Toc167092818)

[Additional Confounders 2](#_Toc167092819)

[References 4](#_Toc167092820)

# Supplementary Methods

## Deviations from the Analytical Plan

First, we restricted the analysis to those aged 10 or more years at the time of their infection. This decision was motivated by the complexity of recognizing and diagnosing mental disorders in the youngest paediatric populations.

Second, we exactly-matched on age, instead of matching on age ± 3 years. We did so because the extent of data allowed this.

Third, we performed matching on the Charlson Comorbidity Index (CCI) [1] that was coded as 0, 1, 2, 3, and 4 or more comorbidities. Subsequently, we used CCI as a continuous measure in regression models. We did so to avoid unnecessary unmatched individuals.

Fourth, we exactly-matched on vaccination status while not considering vaccinations that occurred <= 14 days before the infection. We did so to account for the fact that vaccinations do not offer immediate protection.

Lastly, we did not implement the sensitivity analysis to examine the risk of being tested (positively or negatively) for SARS-CoV-2 infection in individuals who had a history of a mental disorder with individuals who had no such history. We did so because of the unexpected technical complexity of such an analysis that would warrant a separate study.

## Exposure

The table below includes the ICD-10 diagnostic codes and corresponding labels of mental disorders used throughout this study.

| ICD-10 code | Name per ICD-10 | Label used in this study |
| --- | --- | --- |
| F10-F19 | Mental and behavioural disorders due to psychoactive substance use | Substance use disorders |
| F20-F29 | Schizophrenia, schizotypal and delusional disorders | Psychotic disorders |
| F30-F39 | Mood [affective] disorders | Affective disorders |
| F40-F48 | Neurotic, stress-related and somatoform disorders | Anxiety disorders |
| F10-F19, F20-F29, F30-F39, F40-F48 | NA | Any mental disorder |

## Additional Confounders

The list of considered medications included prescription for antihypertensives (ATC code C02), aspirin (ATC codes B01AC06, N02BA01, N02BA51), statins (ATC code C10AA), antithrombotic agents (ATC code B01), non-steroidal anti-inflammatory medications (ATC code M01A), bisphosphonates (ATC codes M05BA and M05BB), oral contraceptives (ATC code G03A), hormone replacement therapy (ATC codes G03C, G03D, G03F), anticonvulsants (ATC code N03), cytostatic chemotherapy (ATC codes L01A, L01B, L01C, L01D, L01E, L01X), radiotherapy (ATC code V10), immunosuppressant medication (ATC code L04), long-acting beta-agonist (ATC code R03AC13, R03AC12), leukotriene receptor antagonists (ATC code R03DC), and inhaled glucocorticoids (ATC code R03BA). We considered prescription of each of the medications or treatment administration in the time period of one year prior to the SARS‑CoV‑2 infection, separately. We selected the prescription medications as in Ranger et al who used this list of medications in the context of pre-existing neuropsychiatric conditions and subsequent severe COVID-19 infection [2].

# References

1. Charlson ME, Pompei P, Ales KL, MacKenzie CR. A new method of classifying prognostic comorbidity in longitudinal studies: Development and validation. J Chronic Dis. 1987;40(5):373-83. doi: 10.1016/0021-9681(87)90171-8.

2. Ranger TA, Clift AK, Patone M, Coupland CAC, Hatch R, Thomas K, et al. Preexisting Neuropsychiatric Conditions and Associated Risk of Severe COVID-19 Infection and Other Acute Respiratory Infections. JAMA Psychiatry. 2022;80(1):57-65. doi: 10.1001/jamapsychiatry.2022.3614.
