# Supplementary material for: Deaths with COVID-19 and from all-causes following first-ever SARS-CoV-2 infection in individuals with preexisting mental disorders: A national cohort study from Czechia
Source: PLoS Med. 2024 Jul 15;21(7):e1004422. doi: 10.1371/journal.pmed.1004422 (PMC11285938; doi:10.1371/journal.pmed.1004422)
Supplement: S1 Table — (DOCX) [file pmed.1004422.s003.docx]

Supplementary Table 1 Unmatched individuals per cohorts

| Cohort | Epoch | Case ascertainment approach | | | |
| --- | --- | --- | --- | --- | --- |
|  |  | diagnosed | | diagnosed and treated | |
|  |  | total, n | unmatched, n (%) | total, n | unmatched, n (%) |
| any mental disorder | 1 | 7509 | 235 (3.13) | 5539 | 418 (7.55) |
| any mental disorder | 2 | 72854 | 39 (0.05) | 57694 | 393 (0.68) |
| any mental disorder | 3 | 99530 | 223 (0.22) | 77366 | 635 (0.82) |
| any mental disorder | 4 | 23320 | 682 (2.92) | 17306 | 984 (5.69) |
| any mental disorder | 5 | 187774 | 453 (0.24) | 136387 | 1384 (1.01) |
| substance use disorders | 1 | 811 | 22 (2.71) | 462 | 28 (6.06) |
| substance use disorders | 2 | 8160 | 0 (0.00) | 5441 | 0 (0.00) |
| substance use disorders | 3 | 12790 | 22 (0.17) | 8083 | 35 (0.43) |
| substance use disorders | 4 | 3089 | 73 (2.36) | 1878 | 83 (4.42) |
| substance use disorders | 5 | 22249 | 44 (0.20) | 13361 | 75 (0.56) |
| psychotic disorders | 1 | 277 | 6 (2.17) | 262 | 15 (5.73) |
| psychotic disorders | 2 | 4300 | 0 (0.00) | 3914 | 0 (0.00) |
| psychotic disorders | 3 | 5418 | 13 (0.24) | 4999 | 31 (0.62) |
| psychotic disorders | 4 | 1134 | 26 (2.29) | 1046 | 40 (3.82) |
| psychotic disorders | 5 | 7647 | 21 (0.27) | 7019 | 38 (0.54) |
| affective disorders | 1 | 1687 | 40 (2.37) | 1548 | 78 (5.04) |
| affective disorders | 2 | 17397 | 1 (0.01) | 16205 | 5 (0.03) |
| affective disorders | 3 | 22931 | 31 (0.14) | 21248 | 83 (0.39) |
| affective disorders | 4 | 5201 | 122 (2.35) | 4781 | 226 (4.73) |
| affective disorders | 5 | 39921 | 61 (0.15) | 36286 | 152 (0.42) |
| anxiety disorders | 1 | 5960 | 163 (2.73) | 4405 | 278 (6.31) |
| anxiety disorders | 2 | 55788 | 30 (0.05) | 44279 | 53 (0.12) |
| anxiety disorders | 3 | 76597 | 135 (0.18) | 60239 | 321 (0.53) |
| anxiety disorders | 4 | 18115 | 472 (2.61) | 13535 | 665 (4.91) |
| anxiety disorders | 5 | 150483 | 272 (0.18) | 110091 | 732 (0.66) |

The results are presented as absolute numbers (n) with proportions (%). The time frames for epochs were: (1) 1^st^ March 2020-30^th^ September 2020 for epoch 1, (2) 1^st^ October 2020-26^th^ December 2020 for epoch 2, (3) 27^th^ December 2020-31^st^ March 2021 for epoch 3, (4) 1^st^ April 2021-31^st^ October 2021 for epoch 4, and (5) 1^st^ November 2021-29^th^ February 2022 for epoch 5. “Diagnosed” refers to cases ascertained by diagnosis per the International Classification of Diseases 10th Revision (ICD-10) diagnostic codes: (1) F10-F19, F20-F29, F30-F39, F40-F48 for any mental disorder, (2) F10-F19 for substance use disorders, (3) F20-F29 for psychotic disorders, (4) F30-F39 for affective disorders, and (5) F40-F48 for anxiety disorders. “Diagnosed and treated” refers to cases ascertained by diagnosis per the above ICD-10 codes coupled with prescription for anxiolytics/hypnotics/sedatives (N05B, N05C), (2) antidepressants (N06A), (3) antipsychotics (N05A) or (4) stimulants (N06B) per the Anatomical Therapeutic Chemical (ATC) classification codes.
