# Supplementary material for: Deaths with COVID-19 and from all-causes following first-ever SARS-CoV-2 infection in individuals with preexisting mental disorders: A national cohort study from Czechia
Source: PLoS Med. 2024 Jul 15;21(7):e1004422. doi: 10.1371/journal.pmed.1004422 (PMC11285938; doi:10.1371/journal.pmed.1004422)
Supplement: S2 Table — (DOCX) [file pmed.1004422.s004.docx]

Supplementary Table 2 Characteristics of unmatched individuals, cases ascertained by diagnosis per the International Classification of Diseases 10th Revision (ICD-10) diagnostic codes

| Epoch | Characteristic | Any mental disorder | | Substance use disorders | | Psychotic disorders | | Affective disorders | | Anxiety disorders | |
| --- | --- | --- | --- | --- | --- | --- | --- | --- | --- | --- | --- |
|  |  | unmatched | matched | unmatched | matched | unmatched | matched | unmatched | matched | unmatched | matched |
| 1 | Total, n | 235 | 7274 | 22 | 789 | 6 | 271 | 40 | 1647 | 163 | 5797 |
|  | Age, mean (SD) | 56.12 (21.86) | 44.36 (17.95) | 48.09 (17.12) | 42.25 (18.27) | 70.33 (24.46) | 50.39 (20.20) | 64.08 (23.02) | 48.17 (17.42) | 54.80 (22.13) | 43.43 (17.54) |
|  | Sex, n (%) |  | | | | | | | | | |
|  | Females | 163 (69.36) | 4684 (64.39) | 9 (40.91) | 317 (40.18) | 2 (33.33) | 156 (57.56) | 29 (72.50) | 1092 (66.30) | 118 (72.39) | 3892 (67.14) |
|  | Infection month, median (IQR) | 5 (3) | 9 (1) | 6 (1.75) | 9 (2) | 5.5 (2.5) | 9 (1) | 5 (3) | 9 (1) | 5 (3) | 9 (1) |
|  | Infection year, median (IQR) | 2020 (0) | 2020 (0) | 2020 (0) | 2020 (0) | 2020 (0) | 2020 (0) | 2020 (0) | 2020 (0) | 2020 (0) | 2020 (0) |
|  | Charlson Comorbidity Index, mean (SD) | 3.32 (2.36) | 1.2 (1.78) | 4.09 (2.39) | 1.33 (1.98) | 3.33 (1.86) | 1.58 (2.11) | 3.08 (1.7) | 1.47 (1.93) | 3.18 (2.38) | 1.14 (1.71) |
| 2 | Total, n | 39 | 72815 | NA | 8160 | NA | 4300 | 1 | 17396 | 30 | 55758 |
|  | Age, mean (SD) | 25.05 (23.60) | 49.48 (18.20) | NA | 48.27 (17.80) | NA | 56.04 (18.37) | 102.00 ( NA) | 53.50 (17.50) | 23.67 (22.54) | 47.86 (17.86) |
|  | Sex, n (%) |  | | | | | | | | | |
|  | Females | 29 (74.36) | 48654 (66.82) | NA | 3111 (38.12) | NA | 2468 (57.40) | 1 (100.00) | 12341 (70.94) | 23 (76.67) | 39126 (70.17) |
|  | Infection month, median (IQR) | 11 (2) | 11 (1) | NA | 11 (2) | NA | 11 (1) | 11 (0) | 11 (1) | 11 (2) | 11 (1) |
|  | Infection year, median (IQR) | 2020 (0) | 2020 (0) | NA | 2020 (0) | NA | 2020 (0) | 2020 (0) | 2020 (0) | 2020 (0) | 2020 (0) |
|  | Charlson Comorbidity Index, mean (SD) | 4.13 (1.69) | 1.55 (2.03) | NA | 1.79 (2.17) | NA | 1.99 (2.26) | 1 (NA) | 1.82 (2.2) | 4.17 (1.44) | 1.43 (1.93) |
| 3 | Total, n | 223 | 99307 | 22 | 12768 | 13 | 5405 | 31 | 22900 | 135 | 76462 |
|  | Age, mean (SD) | 54.47 (21.34) | 47.82 (17.23) | 53.09 (20.58) | 44.98 (16.26) | 65.69 (15.22) | 50.58 (17.04) | 58.19 (17.17) | 51.81 (16.53) | 53.67 (22.62) | 46.79 (17.04) |
|  | Sex, n (%) |  | | | | | | | | | |
|  | Females | 142 (63.68) | 64115 (64.56) | 10 (45.45) | 4466 (34.98) | 6 (46.15) | 2792 (51.66) | 20 (64.52) | 15971 (69.74) | 93 (68.89) | 52333 (68.44) |
|  | Vaccination status, n (%) |  | | | | | | | | | |
|  | Not vaccinated | 35 (15.70) | 98185 (98.87) | 2 (9.09) | 12673 (99.26) | 0 (0.00) | 5281 (97.71) | 0 (0.00) | 22576 (98.59) | 20 (14.81) | 75648 (98.94) |
|  | First dose | 134 (60.09) | 993 (1.00) | 15 (68.18) | 80 (0.63) | 10 (76.92) | 117 (2.16) | 19 (61.29) | 285 (1.24) | 78 (57.78) | 713 (0.93) |
|  | Full vaccination | 54 (24.22) | 129 (0.13) | 5 (22.73) | 15 (0.12) | 3 (23.08) | 7 (0.13) | 12 (38.71) | 39 (0.17) | 37 (27.41) | 101 (0.13) |
|  | Booster | 0 (0.00) | 0 (0.00) | 0 (0.00) | 0 (0.00) | 0 (0.00) | 0 (0.00) | 0 (0.00) | 0 (0.00) | 0 (0.00) | 0 (0.00) |
|  | Infection month, median (IQR) | 2 (1) | 2 (2) | 2 (0) | 2 (2) | 2 (1) | 2 (2) | 2 (1) | 2 (2) | 2 (2) | 2 (2) |
|  | Infection year, median (IQR) | 2021 (0) | 2021 (0) | 2021 (0) | 2021 (0) | 2021 (0) | 2021 (0) | 2021 (0) | 2021 (0) | 2021 (0) | 2021 (0) |
|  | Charlson Comorbidity Index, mean (SD) | 3.33 (2.54) | 1.43 (1.89) | 3.5 (2.82) | 1.54 (2.02) | 1.46 (1.2) | 1.62 (2.07) | 3.39 (2.92) | 1.68 (2.06) | 3.44 (2.63) | 1.36 (1.82) |
| 4 | Total, n | 682 | 22638 | 73 | 3016 | 26 | 1108 | 122 | 5079 | 472 | 17643 |
|  | Age, mean (SD) | 48.48 (20.49) | 44.12 (17.48) | 51.33 (19.91) | 42.05 (16.23) | 56.54 (23.29) | 47.80 (16.87) | 52.39 (19.80) | 49.20 (16.42) | 46.28 (19.93) | 43.03 (17.30) |
|  | Sex, n (%) |  | | | | | | | | | |
|  | Females | 462 (67.74) | 14589 (64.44) | 24 (32.88) | 1131 (37.50) | 18 (69.23) | 551 (49.73) | 81 (66.39) | 3500 (68.91) | 341 (72.25) | 12104 (68.61) |
|  | Vaccination status, n (%) |  | | | | | | | | | |
|  | Not vaccinated | 269 (39.44) | 18897 (83.47) | 34 (46.58) | 2660 (88.20) | 6 (23.08) | 934 (84.30) | 46 (37.70) | 4105 (80.82) | 175 (37.08) | 14707 (83.36) |
|  | First dose | 193 (28.30) | 727 (3.21) | 22 (30.14) | 65 (2.16) | 10 (38.46) | 42 (3.79) | 36 (29.51) | 188 (3.70) | 152 (32.20) | 566 (3.21) |
|  | Full vaccination | 217 (31.82) | 3014 (13.31) | 17 (23.29) | 291 (9.65) | 9 (34.62) | 132 (11.91) | 38 (31.15) | 786 (15.48) | 143 (30.30) | 2370 (13.43) |
|  | Booster | 3 (0.44) | 0 (0.00) | 0 (0.00) | 0 (0.00) | 1 (3.85) | 0 (0.00) | 2 (1.64) | 0 (0.00) | 2 (0.42) | 0 (0.00) |
|  | Infection month, median (IQR) | 7 (3) | 5 (6) | 7 (3) | 4 (5) | 7.5 (3.75) | 5 (6) | 7 (3.75) | 5 (6) | 7 (3) | 5 (6) |
|  | Infection year, median (IQR) | 2021 (0) | 2021 (0) | 2021 (0) | 2021 (0) | 2021 (0) | 2021 (0) | 2021 (0) | 2021 (0) | 2021 (0) | 2021 (0) |
|  | Charlson Comorbidity Index, mean (SD) | 2.91 (2.14) | 1.19 (1.72) | 3.05 (1.88) | 1.3 (1.82) | 2.15 (1.54) | 1.35 (1.85) | 3.02 (2.36) | 1.46 (1.9) | 2.8 (2.1) | 1.14 (1.66) |
| 5 | Total, n | 453 | 187321 | 44 | 22205 | 21 | 7626 | 61 | 39860 | 272 | 150211 |
|  | Age, mean (SD) | 48.68 (22.93) | 42.22 (16.79) | 52.00 (19.71) | 40.97 (15.69) | 66.05 (26.88) | 46.30 (16.80) | 57.74 (21.19) | 46.58 (16.09) | 46.46 (23.94) | 41.30 (16.59) |
|  | Sex, n (%) |  | | | | | | | | | |
|  | Females | 286 (63.13) | 123962 (66.18) | 10 (22.73) | 8989 (40.48) | 10 (47.62) | 4126 (54.10) | 37 (60.66) | 28043 (70.35) | 171 (62.87) | 104241 (69.40) |
|  | Vaccination status, n (%) |  | | | | | | | | | |
|  | Not vaccinated | 48 (10.60) | 74090 (39.55) | 0 (0.00) | 10021 (45.13) | 2 (9.52) | 2962 (38.84) | 1 (1.64) | 13809 (34.64) | 23 (8.46) | 59521 (39.62) |
|  | First dose | 275 (60.71) | 2791 (1.49) | 40 (90.91) | 475 (2.14) | 12 (57.14) | 143 (1.88) | 42 (68.85) | 538 (1.35) | 165 (60.66) | 2224 (1.48) |
|  | Full vaccination | 47 (10.38) | 83372 (44.51) | 1 (2.27) | 9214 (41.50) | 3 (14.29) | 3413 (44.75) | 3 (4.92) | 18612 (46.69) | 24 (8.82) | 67149 (44.70) |
|  | Booster | 83 (18.32) | 27068 (14.45) | 3 (6.82) | 2495 (11.24) | 4 (19.05) | 1108 (14.53) | 15 (24.59) | 6901 (17.31) | 60 (22.06) | 21317 (14.19) |
|  | Infection month, median (IQR) | 11 (10) | 2 (10) | 2 (9) | 2 (10) | 2 (9) | 2 (9) | 11 (10) | 2 (10) | 11 (10) | 2 (10) |
|  | Infection year, median (IQR) | 2021 (1) | 2022 (1) | 2022 (1) | 2022 (1) | 2022 (1) | 2022 (1) | 2021 (1) | 2022 (1) | 2021 (1) | 2022 (1) |
|  | Charlson Comorbidity Index, mean (SD) | 3.34 (2.14) | 1.12 (1.62) | 3.5 (2.14) | 1.21 (1.73) | 3 (1.73) | 1.29 (1.78) | 3.25 (1.65) | 1.34 (1.78) | 3.34 (2.16) | 1.07 (1.56) |

The results are presented as absolute numbers (n) with proportions (%), means with standard deviations (SD), and medians with interquartile ranges (IQR). The time frames for epochs were: (1) 1^st^ March 2020-30^th^ September 2020 for epoch 1, (2) 1^st^ October 2020-26^th^ December 2020 for epoch 2, (3) 27^th^ December 2020-31^st^ March 2021 for epoch 3, (4) 1^st^ April 2021-31^st^ October 2021 for epoch 4, and (5) 1^st^ November 2021-29^th^ February 2022 for epoch 5. “Matched” and “unmatched” refer to people with the respective mental disorder who were included in the study and those with the same mental disorder who could not be included due to a lack of matched counterparts, respectively. The International Classification of Diseases 10^th^ Revision (ICD-10) diagnostic codes were (1) F10-F19, F20-F29, F30-F39, F40-F48 for any mental disorder, (2) F10-F19 for substance use disorders, (3) F20-F29 for psychotic disorders, (4) F30-F39 for affective disorders, and (5) F40-F48 for anxiety disorders.
