# Supplementary material for: Deaths with COVID-19 and from all-causes following first-ever SARS-CoV-2 infection in individuals with preexisting mental disorders: A national cohort study from Czechia
Source: PLoS Med. 2024 Jul 15;21(7):e1004422. doi: 10.1371/journal.pmed.1004422 (PMC11285938; doi:10.1371/journal.pmed.1004422)
Supplement: S4 Table — (DOCX) [file pmed.1004422.s006.docx]

Supplementary Table 4 Number of matches for cases ascertained by diagnosis per the International Classification of Diseases 10th Revision (ICD-10) diagnostic codes

| Cohort | Epoch | Number of matches | | | | |
| --- | --- | --- | --- | --- | --- | --- |
|  |  | 1 | 2 | 3 | 4 | 5 |
| any mental disorder | 1 | 490 (6.74) | 726 (9.98) | 919 (12.63) | 845 (11.62) | 4294 (59.03) |
| any mental disorder | 2 | 518 (0.71) | 2970 (4.08) | 8726 (11.98) | 12757 (17.52) | 47844 (65.71) |
| any mental disorder | 3 | 1027 (1.03) | 4712 (4.74) | 9940 (10.01) | 14683 (14.79) | 68945 (69.43) |
| any mental disorder | 4 | 1184 (5.23) | 1780 (7.86) | 2477 (10.94) | 2803 (12.38) | 14394 (63.58) |
| any mental disorder | 5 | 2647 (1.41) | 9740 (5.20) | 19561 (10.44) | 25440 (13.58) | 129933 (69.36) |
| substance use disorders | 1 | 31 (3.93) | 24 (3.04) | 23 (2.92) | 33 (4.18) | 678 (85.93) |
| substance use disorders | 2 | 3 (0.04) | 8 (0.10) | 5 (0.06) | 12 (0.15) | 8132 (99.66) |
| substance use disorders | 3 | 16 (0.13) | 21 (0.16) | 29 (0.23) | 32 (0.25) | 12670 (99.23) |
| substance use disorders | 4 | 68 (2.25) | 59 (1.96) | 51 (1.69) | 49 (1.62) | 2789 (92.47) |
| substance use disorders | 5 | 64 (0.29) | 74 (0.33) | 60 (0.27) | 93 (0.42) | 21914 (98.69) |
| psychotic disorders | 1 | 5 (1.85) | 12 (4.43) | 6 (2.21) | 12 (4.43) | 236 (87.08) |
| psychotic disorders | 2 | 1 (0.02) | 2 (0.05) | 2 (0.05) | 6 (0.14) | 4289 (99.74) |
| psychotic disorders | 3 | 18 (0.33) | 18 (0.33) | 12 (0.22) | 16 (0.30) | 5341 (98.82) |
| psychotic disorders | 4 | 21 (1.90) | 17 (1.53) | 26 (2.35) | 19 (1.71) | 1025 (92.51) |
| psychotic disorders | 5 | 18 (0.24) | 15 (0.20) | 17 (0.22) | 21 (0.28) | 7555 (99.07) |
| affective disorders | 1 | 56 (3.40) | 69 (4.19) | 72 (4.37) | 59 (3.58) | 1391 (84.46) |
| affective disorders | 2 | 3 (0.02) | 2 (0.01) | 5 (0.03) | 20 (0.11) | 17366 (99.83) |
| affective disorders | 3 | 47 (0.21) | 47 (0.21) | 47 (0.21) | 110 (0.48) | 22649 (98.90) |
| affective disorders | 4 | 121 (2.38) | 134 (2.64) | 134 (2.64) | 149 (2.93) | 4541 (89.41) |
| affective disorders | 5 | 94 (0.24) | 106 (0.27) | 145 (0.36) | 269 (0.67) | 39246 (98.46) |
| anxiety disorders | 1 | 282 (4.86) | 422 (7.28) | 584 (10.07) | 498 (8.59) | 4011 (69.19) |
| anxiety disorders | 2 | 161 (0.29) | 836 (1.50) | 2165 (3.88) | 5219 (9.36) | 47377 (84.97) |
| anxiety disorders | 3 | 413 (0.54) | 1537 (2.01) | 3587 (4.69) | 6493 (8.49) | 64432 (84.27) |
| anxiety disorders | 4 | 658 (3.73) | 976 (5.53) | 1397 (7.92) | 1544 (8.75) | 13068 (74.07) |
| anxiety disorders | 5 | 1102 (0.73) | 3907 (2.60) | 8172 (5.44) | 14714 (9.80) | 122316 (81.43) |

The results are presented as absolute numbers (n) with proportions (%). The time frames for epochs were: (1) 1^st^ March 2020-30^th^ September 2020 for epoch 1, (2) 1^st^ October 2020-26^th^ December 2020 for epoch 2, (3) 27^th^ December 2020-31^st^ March 2021 for epoch 3, (4) 1^st^ April 2021-31^st^ October 2021 for epoch 4, and (5) 1^st^ November 2021-29^th^ February 2022 for epoch 5. The International Classification of Diseases 10^th^ Revision (ICD-10) diagnostic codes were (1) F10-F19, F20-F29, F30-F39, F40-F48 for any mental disorder, (2) F10-F19 for substance use disorders, (3) F20-F29 for psychotic disorders, (4) F30-F39 for affective disorders, and (5) F40-F48 for anxiety disorders.
