# Supplementary material for: Deaths with COVID-19 and from all-causes following first-ever SARS-CoV-2 infection in individuals with preexisting mental disorders: A national cohort study from Czechia
Source: PLoS Med. 2024 Jul 15;21(7):e1004422. doi: 10.1371/journal.pmed.1004422 (PMC11285938; doi:10.1371/journal.pmed.1004422)
Supplement: S5 Table — (DOCX) [file pmed.1004422.s007.docx]

Supplementary Table 5 Number of matches for cases ascertained by diagnosis per the International Classification of Diseases 10^th^ Revision (ICD-10) diagnostic codes coupled with prescription for psychopharmaceuticals per the Anatomical Therapeutic Chemical (ATC) classification codes

| Cohort | Epoch | Number of matches | | | | |
| --- | --- | --- | --- | --- | --- | --- |
|  |  | 1 | 2 | 3 | 4 | 5 |
| any mental disorder | 1 | 780 (15.23) | 712 (13.90) | 495 (9.67) | 517 (10.10) | 2617 (51.10) |
| any mental disorder | 2 | 6834 (11.93) | 7978 (13.92) | 8324 (14.53) | 5273 (9.20) | 28892 (50.42) |
| any mental disorder | 3 | 6322 (8.24) | 10470 (13.65) | 9776 (12.74) | 7070 (9.21) | 43093 (56.16) |
| any mental disorder | 4 | 1818 (11.14) | 2293 (14.05) | 1955 (11.98) | 1566 (9.59) | 8690 (53.24) |
| any mental disorder | 5 | 9822 (7.28) | 15637 (11.58) | 16006 (11.86) | 12727 (9.43) | 80811 (59.86) |
| substance use disorders | 1 | 45 (10.37) | 15 (3.46) | 23 (5.30) | 29 (6.68) | 322 (74.19) |
| substance use disorders | 2 | 7 (0.13) | 13 (0.24) | 31 (0.57) | 54 (0.99) | 5336 (98.07) |
| substance use disorders | 3 | 18 (0.22) | 25 (0.31) | 44 (0.55) | 72 (0.89) | 7889 (98.02) |
| substance use disorders | 4 | 74 (4.12) | 64 (3.57) | 59 (3.29) | 58 (3.23) | 1540 (85.79) |
| substance use disorders | 5 | 62 (0.47) | 78 (0.59) | 103 (0.78) | 170 (1.28) | 12873 (96.89) |
| psychotic disorders | 1 | 13 (5.26) | 20 (8.10) | 14 (5.67) | 14 (5.67) | 186 (75.30) |
| psychotic disorders | 2 | 1 (0.03) | 9 (0.23) | 10 (0.26) | 19 (0.49) | 3875 (99.00) |
| psychotic disorders | 3 | 35 (0.70) | 27 (0.54) | 20 (0.40) | 15 (0.30) | 4871 (98.05) |
| psychotic disorders | 4 | 36 (3.58) | 29 (2.88) | 24 (2.39) | 28 (2.78) | 889 (88.37) |
| psychotic disorders | 5 | 28 (0.40) | 33 (0.47) | 32 (0.46) | 40 (0.57) | 6848 (98.09) |
| affective disorders | 1 | 136 (9.25) | 105 (7.14) | 83 (5.65) | 71 (4.83) | 1075 (73.13) |
| affective disorders | 2 | 27 (0.17) | 548 (3.38) | 989 (6.10) | 840 (5.19) | 13796 (85.16) |
| affective disorders | 3 | 153 (0.72) | 392 (1.85) | 732 (3.46) | 981 (4.64) | 18907 (89.33) |
| affective disorders | 4 | 221 (4.85) | 257 (5.64) | 222 (4.87) | 237 (5.20) | 3618 (79.43) |
| affective disorders | 5 | 306 (0.85) | 747 (2.07) | 1307 (3.62) | 1292 (3.58) | 32482 (89.89) |
| anxiety disorders | 1 | 497 (12.04) | 536 (12.99) | 345 (8.36) | 386 (9.35) | 2363 (57.26) |
| anxiety disorders | 2 | 2449 (5.54) | 4808 (10.87) | 5646 (12.77) | 4677 (10.58) | 26646 (60.25) |
| anxiety disorders | 3 | 2534 (4.23) | 5899 (9.85) | 6789 (11.33) | 5935 (9.91) | 38761 (64.69) |
| anxiety disorders | 4 | 1111 (8.63) | 1363 (10.59) | 1458 (11.33) | 1273 (9.89) | 7665 (59.56) |
| anxiety disorders | 5 | 4985 (4.56) | 9242 (8.45) | 10893 (9.96) | 10203 (9.33) | 74036 (67.70) |

The results are presented as absolute numbers (n) with proportions (%). The time frames for epochs were: (1) 1^st^ March 2020-30^th^ September 2020 for epoch 1, (2) 1^st^ October 2020-26^th^ December 2020 for epoch 2, (3) 27^th^ December 2020-31^st^ March 2021 for epoch 3, (4) 1^st^ April 2021-31^st^ October 2021 for epoch 4, and (5) 1^st^ November 2021-29^th^ February 2022 for epoch 5. The International Classification of Diseases 10^th^ Revision (ICD-10) diagnostic codes were (1) F10-F19, F20-F29, F30-F39, F40-F48 for any mental disorder, (2) F10-F19 for substance use disorders, (3) F20-F29 for psychotic disorders, (4) F30-F39 for affective disorders, and (5) F40-F48 for anxiety disorders. The considered psychopharmaceuticals per the Anatomical Therapeutic Chemical (ATC) classification codes were (1) anxiolytics/hypnotics/sedatives (N05B, N05C), (2) antidepressants (N06A), (3) antipsychotics (N05A), and (4) stimulants (N06B).
