# Supplementary material for: Deaths with COVID-19 and from all-causes following first-ever SARS-CoV-2 infection in individuals with preexisting mental disorders: A national cohort study from Czechia
Source: PLoS Med. 2024 Jul 15;21(7):e1004422. doi: 10.1371/journal.pmed.1004422 (PMC11285938; doi:10.1371/journal.pmed.1004422)
Supplement: S6 Table — (DOCX) [file pmed.1004422.s008.docx]

Supplementary Table 6 Descriptive statistics on additional confounders for cases ascertained by diagnosis per the International Classification of Diseases 10th Revision (ICD-10) diagnostic codes

| Epoch | Characteristic | Any mental disorder | | Substance use disorders | | Psychotic disorders | | Affective disorders | | Anxiety disorders | |
| --- | --- | --- | --- | --- | --- | --- | --- | --- | --- | --- | --- |
|  |  | unexposed | exposed | unexposed | exposed | unexposed | exposed | unexposed | exposed | unexposed | exposed |
| 1 | Region of permanent residency, n (%) |  | | | | | | | | | |
|  | Prague region | 6228 (21.08) | 1910 (26.26) | 831 (22.64) | 198 (25.10) | 250 (19.61) | 63 (23.25) | 1575 (20.72) | 450 (27.32) | 5296 (21.25) | 1548 (26.70) |
|  | Central-Bohemian region | 4573 (15.48) | 1062 (14.60) | 536 (14.60) | 117 (14.83) | 176 (13.80) | 35 (12.92) | 1135 (14.93) | 240 (14.57) | 3867 (15.51) | 855 (14.75) |
|  | South-Bohemian region | 1288 (4.36) | 292 (4.01) | 151 (4.11) | 22 (2.79) | 62 (4.86) | 19 (7.01) | 323 (4.25) | 64 (3.89) | 1099 (4.41) | 235 (4.05) |
|  | Plzen region | 1544 (5.23) | 372 (5.11) | 195 (5.31) | 34 (4.31) | 87 (6.82) | 14 (5.17) | 453 (5.96) | 85 (5.16) | 1304 (5.23) | 302 (5.21) |
|  | Karlovy Vary region | 728 (2.46) | 182 (2.50) | 89 (2.43) | 24 (3.04) | 28 (2.20) | 11 (4.06) | 206 (2.71) | 39 (2.37) | 648 (2.60) | 147 (2.54) |
|  | Usti region | 1486 (5.03) | 365 (5.02) | 182 (4.96) | 38 (4.82) | 68 (5.33) | 14 (5.17) | 394 (5.18) | 96 (5.83) | 1250 (5.02) | 282 (4.86) |
|  | Liberec region | 989 (3.35) | 205 (2.82) | 105 (2.86) | 16 (2.03) | 37 (2.90) | 5 (1.85) | 232 (3.05) | 35 (2.13) | 833 (3.34) | 177 (3.05) |
|  | Hradec Kralove region | 1020 (3.45) | 217 (2.98) | 127 (3.46) | 28 (3.55) | 46 (3.61) | 6 (2.21) | 249 (3.28) | 53 (3.22) | 859 (3.45) | 169 (2.92) |
|  | Pardubice region | 1231 (4.17) | 284 (3.90) | 133 (3.62) | 18 (2.28) | 60 (4.71) | 8 (2.95) | 313 (4.12) | 94 (5.71) | 1073 (4.30) | 215 (3.71) |
|  | Vysocina region | 1287 (4.36) | 250 (3.44) | 150 (4.09) | 23 (2.92) | 56 (4.39) | 8 (2.95) | 330 (4.34) | 66 (4.01) | 1083 (4.35) | 188 (3.24) |
|  | South-Moravian region | 2920 (9.88) | 621 (8.54) | 340 (9.26) | 58 (7.35) | 117 (9.18) | 27 (9.96) | 739 (9.72) | 161 (9.78) | 2463 (9.88) | 479 (8.26) |
|  | Olomouc region | 1486 (5.03) | 412 (5.66) | 181 (4.93) | 43 (5.45) | 63 (4.94) | 18 (6.64) | 435 (5.72) | 84 (5.10) | 1225 (4.91) | 333 (5.74) |
|  | Zlin region | 1518 (5.14) | 311 (4.28) | 173 (4.71) | 43 (5.45) | 65 (5.10) | 12 (4.43) | 407 (5.35) | 63 (3.83) | 1242 (4.98) | 244 (4.21) |
|  | Moravian-Silesian region | 3224 (10.91) | 791 (10.87) | 473 (12.89) | 127 (16.10) | 158 (12.39) | 31 (11.44) | 807 (10.62) | 117 (7.10) | 2662 (10.68) | 623 (10.75) |
|  | Abroad | 27 (0.09) | 0 (0.00) | 4 (0.11) | 0 (0.00) | 2 (0.16) | 0 (0.00) | 3 (0.04) | 0 (0.00) | 21 (0.08) | 0 (0.00) |
|  | Prescription medications, n (%) |  | | | | | | | | | |
|  | antihypertensives | 4436 (15.01) | 1516 (20.84) | 578 (15.75) | 146 (18.50) | 248 (19.45) | 44 (16.24) | 1329 (17.48) | 385 (23.38) | 3771 (15.13) | 1219 (21.03) |
|  | aspirin | 1377 (4.66) | 422 (5.80) | 215 (5.86) | 42 (5.32) | 95 (7.45) | 21 (7.75) | 491 (6.46) | 119 (7.23) | 1051 (4.22) | 312 (5.38) |
|  | statins | 2778 (9.40) | 788 (10.83) | 381 (10.38) | 69 (8.75) | 208 (16.31) | 33 (12.18) | 999 (13.14) | 230 (13.96) | 2184 (8.76) | 599 (10.33) |
|  | antithrombotic agents | 5338 (18.06) | 1803 (24.79) | 706 (19.24) | 179 (22.69) | 307 (24.08) | 67 (24.72) | 1693 (22.27) | 480 (29.14) | 4468 (17.93) | 1401 (24.17) |
|  | non-steroidal anti-inflammatory medications | 5738 (19.42) | 1898 (26.09) | 715 (19.48) | 175 (22.18) | 296 (23.22) | 56 (20.66) | 1829 (24.06) | 513 (31.15) | 4830 (19.38) | 1515 (26.13) |
|  | bisphosphonates | 128 (0.43) | 52 (0.71) | 25 (0.68) | 2 (0.25) | 8 (0.63) | 0 (0.00) | 47 (0.62) | 18 (1.09) | 108 (0.43) | 39 (0.67) |
|  | oral contraceptives | 1 (0.00) | 0 (0.00) | 0 (0.00) | 0 (0.00) | 0 (0.00) | 0 (0.00) | 0 (0.00) | 0 (0.00) | 0 (0.00) | 0 (0.00) |
|  | hormone replacement therapy | 1023 (3.46) | 322 (4.43) | 81 (2.21) | 13 (1.65) | 35 (2.75) | 7 (2.58) | 297 (3.91) | 82 (4.98) | 921 (3.70) | 270 (4.66) |
|  | anticonvulsants | 822 (2.78) | 746 (10.26) | 143 (3.90) | 78 (9.89) | 65 (5.10) | 94 (34.69) | 347 (4.57) | 281 (17.06) | 751 (3.01) | 548 (9.45) |
|  | cytostatic chemotherapy | 114 (0.39) | 38 (0.52) | 23 (0.63) | 4 (0.51) | 8 (0.63) | 1 (0.37) | 49 (0.64) | 8 (0.49) | 94 (0.38) | 31 (0.53) |
|  | radiotherapy | 8 (0.03) | 3 (0.04) | 2 (0.05) | 1 (0.13) | 2 (0.16) | 0 (0.00) | 2 (0.03) | 0 (0.00) | 7 (0.03) | 2 (0.03) |
|  | immunosuppressant medication | 352 (1.19) | 104 (1.43) | 53 (1.44) | 3 (0.38) | 20 (1.57) | 2 (0.74) | 135 (1.78) | 29 (1.76) | 301 (1.21) | 85 (1.47) |
|  | long-acting beta-agonist | 104 (0.35) | 34 (0.47) | 21 (0.57) | 6 (0.76) | 9 (0.71) | 0 (0.00) | 39 (0.51) | 10 (0.61) | 92 (0.37) | 26 (0.45) |
|  | leukotriene receptor antagonists | 296 (1.00) | 83 (1.14) | 39 (1.06) | 7 (0.89) | 18 (1.41) | 1 (0.37) | 89 (1.17) | 17 (1.03) | 250 (1.00) | 71 (1.22) |
|  | inhaled glucocorticoids | 487 (1.65) | 132 (1.81) | 70 (1.91) | 11 (1.39) | 25 (1.96) | 8 (2.95) | 134 (1.76) | 41 (2.49) | 426 (1.71) | 105 (1.81) |
|  | Number of inpatient contacts, mean (SD) | 0.78 (1.75) | 1.20 (2.26) | 0.92 (2.15) | 1.77 (2.99) | 1.17 (2.22) | 1.92 (3.06) | 0.98 (1.95) | 1.53 (2.66) | 0.78 (1.74) | 1.22 (2.33) |
|  | Number of outpatient contacts, mean (SD) | 79.73 (103.42) | 108.51 (128.98) | 81.55 (104.31) | 105.47 (157.56) | 105.89 (157.91) | 140.20 (217.46) | 97.11 (122.78) | 131.95 (146.93) | 81.23 (105.26) | 110.03 (122.87) |
| 2 | Region of permanent residency, n (%) |  | | | | | | | | | |
|  | Prague region | 28882 (8.95) | 8657 (11.89) | 3774 (9.26) | 1060 (12.99) | 2037 (9.48) | 369 (8.58) | 8022 (9.23) | 2444 (14.05) | 24318 (9.14) | 6715 (12.04) |
|  | Central-Bohemian region | 40650 (12.59) | 9602 (13.19) | 5014 (12.31) | 938 (11.50) | 2663 (12.40) | 672 (15.63) | 11012 (12.67) | 2308 (13.27) | 33945 (12.76) | 7371 (13.22) |
|  | South-Bohemian region | 20490 (6.35) | 4307 (5.91) | 2505 (6.15) | 365 (4.47) | 1292 (6.01) | 241 (5.60) | 5490 (6.32) | 1050 (6.04) | 16828 (6.32) | 3388 (6.08) |
|  | Plzen region | 17115 (5.30) | 4034 (5.54) | 2294 (5.63) | 367 (4.50) | 1166 (5.43) | 244 (5.67) | 4731 (5.44) | 984 (5.66) | 14253 (5.36) | 3173 (5.69) |
|  | Karlovy Vary region | 6240 (1.93) | 1658 (2.28) | 844 (2.07) | 180 (2.21) | 435 (2.03) | 130 (3.02) | 1826 (2.10) | 327 (1.88) | 5076 (1.91) | 1320 (2.37) |
|  | Usti region | 23580 (7.30) | 5477 (7.52) | 3008 (7.38) | 642 (7.87) | 1610 (7.50) | 345 (8.02) | 6267 (7.21) | 1355 (7.79) | 19452 (7.31) | 4143 (7.43) |
|  | Liberec region | 14653 (4.54) | 3305 (4.54) | 1828 (4.49) | 309 (3.79) | 954 (4.44) | 149 (3.47) | 3805 (4.38) | 778 (4.47) | 12040 (4.52) | 2630 (4.72) |
|  | Hradec Kralove region | 18951 (5.87) | 4058 (5.57) | 2252 (5.53) | 392 (4.80) | 1236 (5.75) | 212 (4.93) | 5072 (5.83) | 816 (4.69) | 15346 (5.77) | 3246 (5.82) |
|  | Pardubice region | 16992 (5.26) | 3655 (5.02) | 2100 (5.15) | 370 (4.53) | 1155 (5.38) | 186 (4.33) | 4585 (5.27) | 1069 (6.15) | 13921 (5.23) | 2709 (4.86) |
|  | Vysocina region | 19013 (5.89) | 4096 (5.63) | 2371 (5.82) | 426 (5.22) | 1222 (5.69) | 302 (7.02) | 4949 (5.69) | 1276 (7.34) | 15603 (5.86) | 2856 (5.12) |
|  | South-Moravian region | 32483 (10.06) | 6465 (8.88) | 4149 (10.18) | 696 (8.53) | 2254 (10.49) | 387 (9.00) | 8555 (9.84) | 1654 (9.51) | 26684 (10.03) | 4796 (8.60) |
|  | Olomouc region | 20677 (6.40) | 4860 (6.67) | 2658 (6.52) | 541 (6.63) | 1383 (6.44) | 265 (6.16) | 5595 (6.44) | 1073 (6.17) | 17095 (6.42) | 3762 (6.75) |
|  | Zlin region | 23410 (7.25) | 4770 (6.55) | 2803 (6.88) | 720 (8.82) | 1497 (6.97) | 308 (7.16) | 6392 (7.35) | 792 (4.55) | 18978 (7.13) | 3650 (6.55) |
|  | Moravian-Silesian region | 39695 (12.29) | 7867 (10.80) | 5135 (12.60) | 1153 (14.13) | 2575 (11.99) | 490 (11.40) | 10616 (12.21) | 1470 (8.45) | 32507 (12.22) | 5996 (10.75) |
|  | Abroad | 53 (0.02) | 4 (0.01) | 7 (0.02) | 1 (0.01) | 1 (0.00) | 0 (0.00) | 15 (0.02) | 0 (0.00) | 43 (0.02) | 3 (0.01) |
|  | Prescription medications, n (%) |  | | | | | | | | | |
|  | antihypertensives | 58932 (18.25) | 16828 (23.11) | 8083 (19.84) | 1757 (21.53) | 4854 (22.60) | 767 (17.84) | 18877 (21.71) | 4427 (25.45) | 48805 (18.34) | 13012 (23.34) |
|  | aspirin | 25862 (8.01) | 6173 (8.48) | 3969 (9.74) | 791 (9.69) | 2828 (13.17) | 510 (11.86) | 9035 (10.39) | 1787 (10.27) | 19614 (7.37) | 4105 (7.36) |
|  | statins | 49249 (15.25) | 10798 (14.83) | 7391 (18.14) | 1037 (12.71) | 4938 (22.99) | 600 (13.95) | 16611 (19.11) | 3223 (18.53) | 37672 (14.16) | 7765 (13.93) |
|  | antithrombotic agents | 78562 (24.33) | 21328 (29.29) | 11003 (27.01) | 2459 (30.13) | 7278 (33.88) | 1235 (28.72) | 26102 (30.03) | 5830 (33.51) | 63411 (23.83) | 15714 (28.18) |
|  | non-steroidal anti-inflammatory medications | 75592 (23.41) | 20842 (28.62) | 9592 (23.54) | 1968 (24.12) | 5821 (27.10) | 718 (16.70) | 23635 (27.19) | 5522 (31.74) | 62570 (23.51) | 16582 (29.74) |
|  | bisphosphonates | 2178 (0.67) | 548 (0.75) | 228 (0.56) | 33 (0.40) | 232 (1.08) | 20 (0.47) | 836 (0.96) | 165 (0.95) | 1776 (0.67) | 430 (0.77) |
|  | oral contraceptives | 6 (0.00) | 1 (0.00) | 0 (0.00) | 1 (0.01) | 0 (0.00) | 0 (0.00) | 1 (0.00) | 0 (0.00) | 5 (0.00) | 0 (0.00) |
|  | hormone replacement therapy | 10765 (3.33) | 3027 (4.16) | 817 (2.01) | 163 (2.00) | 510 (2.37) | 57 (1.33) | 3161 (3.64) | 807 (4.64) | 9753 (3.67) | 2561 (4.59) |
|  | anticonvulsants | 14892 (4.61) | 10522 (14.45) | 2681 (6.58) | 1323 (16.21) | 1689 (7.86) | 1448 (33.67) | 6150 (7.07) | 3671 (21.10) | 13471 (5.06) | 7407 (13.28) |
|  | cytostatic chemotherapy | 1983 (0.61) | 464 (0.64) | 351 (0.86) | 37 (0.45) | 217 (1.01) | 15 (0.35) | 729 (0.84) | 127 (0.73) | 1547 (0.58) | 351 (0.63) |
|  | radiotherapy | 113 (0.03) | 28 (0.04) | 16 (0.04) | 0 (0.00) | 6 (0.03) | 1 (0.02) | 30 (0.03) | 8 (0.05) | 94 (0.04) | 23 (0.04) |
|  | immunosuppressant medication | 5263 (1.63) | 1324 (1.82) | 853 (2.09) | 105 (1.29) | 466 (2.17) | 44 (1.02) | 1862 (2.14) | 373 (2.14) | 4535 (1.70) | 1031 (1.85) |
|  | long-acting beta-agonist | 1921 (0.59) | 538 (0.74) | 320 (0.79) | 59 (0.72) | 202 (0.94) | 28 (0.65) | 674 (0.78) | 156 (0.90) | 1516 (0.57) | 387 (0.69) |
|  | leukotriene receptor antagonists | 3237 (1.00) | 951 (1.31) | 414 (1.02) | 51 (0.62) | 248 (1.15) | 20 (0.47) | 1023 (1.18) | 241 (1.39) | 2894 (1.09) | 801 (1.44) |
|  | inhaled glucocorticoids | 6591 (2.04) | 1776 (2.44) | 913 (2.24) | 117 (1.43) | 549 (2.56) | 53 (1.23) | 2050 (2.36) | 421 (2.42) | 5612 (2.11) | 1470 (2.64) |
|  | Number of inpatient contacts, mean (SD) | 1.08 (2.07) | 1.56 (2.77) | 1.28 (2.49) | 2.47 (4.09) | 1.61 (2.79) | 2.28 (4.18) | 1.40 (2.50) | 1.94 (3.32) | 1.09 (2.09) | 1.53 (2.79) |
|  | Number of outpatient contacts, mean (SD) | 103.36 (154.47) | 132.87 (180.53) | 109.85 (167.46) | 135.65 (214.80) | 133.69 (195.39) | 209.18 (349.00) | 127.95 (184.42) | 154.62 (192.61) | 106.94 (161.93) | 127.82 (149.58) |
| 3 | Region of permanent residency, n (%) |  | | | | | | | | | |
|  | Prague region | 45717 (10.30) | 13180 (13.27) | 6882 (10.82) | 1838 (14.40) | 2876 (10.71) | 701 (12.97) | 11947 (10.48) | 3510 (15.33) | 38155 (10.53) | 10149 (13.27) |
|  | Central-Bohemian region | 64603 (14.56) | 14587 (14.69) | 9421 (14.81) | 1777 (13.92) | 3925 (14.61) | 725 (13.41) | 16407 (14.40) | 3410 (14.89) | 53111 (14.66) | 11438 (14.96) |
|  | South-Bohemian region | 25435 (5.73) | 5339 (5.38) | 3547 (5.58) | 540 (4.23) | 1550 (5.77) | 307 (5.68) | 6594 (5.79) | 1301 (5.68) | 20778 (5.73) | 4161 (5.44) |
|  | Plzen region | 29132 (6.57) | 6646 (6.69) | 4268 (6.71) | 786 (6.16) | 1785 (6.65) | 329 (6.09) | 7290 (6.40) | 1570 (6.86) | 23563 (6.50) | 5236 (6.85) |
|  | Karlovy Vary region | 15429 (3.48) | 3790 (3.82) | 2307 (3.63) | 542 (4.24) | 956 (3.56) | 216 (4.00) | 4122 (3.62) | 629 (2.75) | 12444 (3.43) | 3055 (4.00) |
|  | Usti region | 33598 (7.57) | 8019 (8.07) | 4954 (7.79) | 1110 (8.69) | 2047 (7.62) | 495 (9.16) | 8798 (7.72) | 1973 (8.62) | 27493 (7.59) | 6032 (7.89) |
|  | Liberec region | 23026 (5.19) | 5391 (5.43) | 3271 (5.14) | 688 (5.39) | 1308 (4.87) | 253 (4.68) | 5895 (5.17) | 1096 (4.79) | 18622 (5.14) | 4269 (5.58) |
|  | Hradec Kralove region | 33252 (7.49) | 6719 (6.77) | 4660 (7.32) | 730 (5.72) | 2030 (7.56) | 348 (6.44) | 8503 (7.46) | 1229 (5.37) | 27043 (7.46) | 5340 (6.98) |
|  | Pardubice region | 26696 (6.02) | 5731 (5.77) | 3723 (5.85) | 634 (4.97) | 1560 (5.81) | 278 (5.14) | 6557 (5.75) | 1635 (7.14) | 21635 (5.97) | 4282 (5.60) |
|  | Vysocina region | 18354 (4.14) | 3811 (3.84) | 2640 (4.15) | 423 (3.31) | 1108 (4.13) | 226 (4.18) | 4588 (4.03) | 1124 (4.91) | 14854 (4.10) | 2701 (3.53) |
|  | South-Moravian region | 41449 (9.34) | 8110 (8.17) | 5941 (9.34) | 1006 (7.88) | 2511 (9.35) | 538 (9.95) | 10589 (9.29) | 1855 (8.10) | 33879 (9.35) | 6082 (7.95) |
|  | Olomouc region | 22894 (5.16) | 5368 (5.41) | 3254 (5.11) | 707 (5.54) | 1443 (5.37) | 248 (4.59) | 5977 (5.24) | 1154 (5.04) | 18572 (5.13) | 4181 (5.47) |
|  | Zlin region | 18904 (4.26) | 3671 (3.70) | 2516 (3.95) | 557 (4.36) | 1097 (4.08) | 201 (3.72) | 4820 (4.23) | 710 (3.10) | 15326 (4.23) | 2797 (3.66) |
|  | Moravian-Silesian region | 45173 (10.18) | 8943 (9.01) | 6233 (9.80) | 1429 (11.19) | 2661 (9.91) | 540 (9.99) | 11867 (10.41) | 1704 (7.44) | 36853 (10.17) | 6738 (8.81) |
|  | Abroad | 66 (0.01) | 2 (0.00) | 6 (0.01) | 1 (0.01) | 2 (0.01) | 0 (0.00) | 13 (0.01) | 0 (0.00) | 52 (0.01) | 1 (0.00) |
|  | Prescription medications, n (%) |  |  |  |  |  |  |  |  |  |  |
|  | antihypertensives | 69733 (15.72) | 20356 (20.50) | 10456 (16.43) | 2372 (18.58) | 4877 (18.16) | 862 (15.95) | 21800 (19.13) | 5196 (22.69) | 57843 (15.96) | 15953 (20.86) |
|  | aspirin | 30484 (6.87) | 7031 (7.08) | 4506 (7.08) | 849 (6.65) | 2408 (8.97) | 465 (8.60) | 10007 (8.78) | 1996 (8.72) | 23207 (6.40) | 4909 (6.42) |
|  | statins | 64744 (14.59) | 14486 (14.59) | 9280 (14.59) | 1480 (11.59) | 4892 (18.21) | 815 (15.08) | 21019 (18.44) | 4229 (18.47) | 50025 (13.80) | 10436 (13.65) |
|  | antithrombotic agents | 94920 (21.39) | 26087 (26.27) | 13542 (21.28) | 3116 (24.40) | 6841 (25.47) | 1299 (24.03) | 30203 (26.50) | 6923 (30.23) | 76655 (21.15) | 19782 (25.87) |
|  | non-steroidal anti-inflammatory medications | 98270 (22.15) | 28142 (28.34) | 13523 (21.25) | 3012 (23.59) | 6648 (24.75) | 999 (18.48) | 30259 (26.55) | 7264 (31.72) | 81534 (22.50) | 22329 (29.20) |
|  | bisphosphonates | 2825 (0.64) | 747 (0.75) | 268 (0.42) | 44 (0.34) | 226 (0.84) | 23 (0.43) | 1027 (0.90) | 235 (1.03) | 2364 (0.65) | 577 (0.75) |
|  | oral contraceptives | 9 (0.00) | 1 (0.00) | 0 (0.00) | 0 (0.00) | 0 (0.00) | 0 (0.00) | 4 (0.00) | 0 (0.00) | 10 (0.00) | 1 (0.00) |
|  | hormone replacement therapy | 13991 (3.15) | 4029 (4.06) | 1218 (1.91) | 231 (1.81) | 691 (2.57) | 113 (2.09) | 4211 (3.69) | 1008 (4.40) | 12752 (3.52) | 3469 (4.54) |
|  | anticonvulsants | 16931 (3.82) | 13556 (13.65) | 3131 (4.92) | 1855 (14.53) | 1579 (5.88) | 1774 (32.82) | 6792 (5.96) | 4828 (21.08) | 15538 (4.29) | 9825 (12.85) |
|  | cytostatic chemotherapy | 2745 (0.62) | 593 (0.60) | 540 (0.85) | 53 (0.42) | 236 (0.88) | 17 (0.31) | 1002 (0.88) | 147 (0.64) | 2197 (0.61) | 469 (0.61) |
|  | radiotherapy | 112 (0.03) | 22 (0.02) | 22 (0.03) | 2 (0.02) | 7 (0.03) | 0 (0.00) | 53 (0.05) | 6 (0.03) | 108 (0.03) | 19 (0.02) |
|  | immunosuppressant medication | 7139 (1.61) | 1824 (1.84) | 1205 (1.89) | 163 (1.28) | 529 (1.97) | 54 (1.00) | 2375 (2.08) | 506 (2.21) | 5935 (1.64) | 1409 (1.84) |
|  | long-acting beta-agonist | 2417 (0.54) | 718 (0.72) | 392 (0.62) | 103 (0.81) | 221 (0.82) | 44 (0.81) | 851 (0.75) | 208 (0.91) | 1963 (0.54) | 506 (0.66) |
|  | leukotriene receptor antagonists | 3934 (0.89) | 1198 (1.21) | 607 (0.95) | 72 (0.56) | 267 (0.99) | 30 (0.56) | 1207 (1.06) | 313 (1.37) | 3573 (0.99) | 1004 (1.31) |
|  | inhaled glucocorticoids | 8407 (1.89) | 2322 (2.34) | 1255 (1.97) | 193 (1.51) | 575 (2.14) | 83 (1.54) | 2644 (2.32) | 610 (2.66) | 7243 (2.00) | 1864 (2.44) |
|  | Number of inpatient contacts, mean (SD) | 0.88 (1.71) | 1.28 (2.28) | 0.94 (1.90) | 1.87 (3.24) | 1.10 (2.13) | 1.96 (3.61) | 1.11 (2.05) | 1.66 (2.91) | 0.90 (1.75) | 1.33 (2.42) |
|  | Number of outpatient contacts, mean (SD) | 84.63 (100.68) | 108.93 (120.57) | 83.68 (106.37) | 96.73 (124.75) | 96.15 (120.17) | 131.31 (231.62) | 102.80 (122.11) | 130.30 (137.28) | 87.80 (105.90) | 111.14 (104.86) |
| 4 | Region of permanent residency, n (%) |  | | | | | | | | | |
|  | Prague region | 10946 (11.48) | 3285 (14.51) | 1595 (11.02) | 421 (13.96) | 620 (11.62) | 150 (13.54) | 2772 (11.51) | 858 (16.89) | 9208 (11.76) | 2609 (14.79) |
|  | Central-Bohemian region | 10905 (11.44) | 2581 (11.40) | 1653 (11.42) | 306 (10.15) | 558 (10.46) | 93 (8.39) | 2704 (11.22) | 603 (11.87) | 8984 (11.47) | 2080 (11.79) |
|  | South-Bohemian region | 7501 (7.87) | 1763 (7.79) | 1128 (7.79) | 177 (5.87) | 415 (7.78) | 80 (7.22) | 1855 (7.70) | 409 (8.05) | 6083 (7.77) | 1381 (7.83) |
|  | Plzen region | 4376 (4.59) | 1064 (4.70) | 642 (4.43) | 122 (4.05) | 258 (4.84) | 37 (3.34) | 1013 (4.20) | 228 (4.49) | 3557 (4.54) | 855 (4.85) |
|  | Karlovy Vary region | 1012 (1.06) | 284 (1.25) | 154 (1.06) | 34 (1.13) | 57 (1.07) | 13 (1.17) | 238 (0.99) | 54 (1.06) | 831 (1.06) | 236 (1.34) |
|  | Usti region | 7135 (7.48) | 1750 (7.73) | 1144 (7.90) | 257 (8.52) | 413 (7.74) | 104 (9.39) | 1863 (7.73) | 393 (7.74) | 5801 (7.41) | 1330 (7.54) |
|  | Liberec region | 3118 (3.27) | 764 (3.37) | 510 (3.52) | 115 (3.81) | 178 (3.34) | 34 (3.07) | 770 (3.20) | 161 (3.17) | 2576 (3.29) | 604 (3.42) |
|  | Hradec Kralove region | 2008 (2.11) | 482 (2.13) | 301 (2.08) | 51 (1.69) | 114 (2.14) | 16 (1.44) | 539 (2.24) | 84 (1.65) | 1718 (2.19) | 406 (2.30) |
|  | Pardubice region | 4237 (4.44) | 952 (4.21) | 674 (4.65) | 106 (3.51) | 257 (4.82) | 41 (3.70) | 1061 (4.40) | 277 (5.45) | 3414 (4.36) | 717 (4.06) |
|  | Vysocina region | 4950 (5.19) | 1091 (4.82) | 797 (5.50) | 125 (4.14) | 307 (5.76) | 82 (7.40) | 1285 (5.33) | 299 (5.89) | 4052 (5.17) | 802 (4.55) |
|  | South-Moravian region | 11518 (12.08) | 2418 (10.68) | 1709 (11.80) | 305 (10.11) | 625 (11.72) | 136 (12.27) | 2872 (11.92) | 554 (10.91) | 9449 (12.07) | 1843 (10.45) |
|  | Olomouc region | 6860 (7.19) | 1694 (7.48) | 1028 (7.10) | 233 (7.73) | 376 (7.05) | 70 (6.32) | 1685 (6.99) | 349 (6.87) | 5581 (7.13) | 1342 (7.61) |
|  | Zlin region | 6686 (7.01) | 1425 (6.29) | 1019 (7.04) | 272 (9.02) | 374 (7.01) | 69 (6.23) | 1739 (7.22) | 249 (4.90) | 5444 (6.95) | 1061 (6.01) |
|  | Moravian-Silesian region | 14084 (14.77) | 3085 (13.63) | 2120 (14.64) | 492 (16.31) | 781 (14.64) | 183 (16.52) | 3692 (15.32) | 561 (11.05) | 11604 (14.82) | 2377 (13.47) |
|  | Abroad | 21 (0.02) | 0 (0.00) | 6 (0.04) | 0 (0.00) | 1 (0.02) | 0 (0.00) | 4 (0.02) | 0 (0.00) | 15 (0.02) | 0 (0.00) |
|  | Prescription medications, n (%) |  | | | | | | | | | |
|  | antihypertensives | 13061 (13.70) | 4581 (20.24) | 2116 (14.61) | 569 (18.87) | 881 (16.52) | 197 (17.78) | 4128 (17.13) | 1117 (21.99) | 10971 (14.01) | 3663 (20.76) |
|  | aspirin | 4952 (5.19) | 1347 (5.95) | 815 (5.63) | 185 (6.13) | 374 (7.01) | 86 (7.76) | 1750 (7.26) | 354 (6.97) | 3846 (4.91) | 956 (5.42) |
|  | statins | 10549 (11.06) | 2642 (11.67) | 1662 (11.48) | 270 (8.95) | 774 (14.51) | 140 (12.64) | 3646 (15.13) | 791 (15.57) | 8160 (10.42) | 1890 (10.71) |
|  | antithrombotic agents | 17175 (18.01) | 5569 (24.60) | 2744 (18.95) | 724 (24.01) | 1238 (23.21) | 264 (23.83) | 5673 (23.55) | 1454 (28.63) | 14073 (17.97) | 4276 (24.24) |
|  | non-steroidal anti-inflammatory medications | 18767 (19.68) | 6043 (26.69) | 2863 (19.77) | 699 (23.18) | 1242 (23.28) | 215 (19.40) | 5953 (24.71) | 1596 (31.42) | 15638 (19.97) | 4827 (27.36) |
|  | bisphosphonates | 478 (0.50) | 146 (0.64) | 55 (0.38) | 7 (0.23) | 40 (0.75) | 8 (0.72) | 188 (0.78) | 36 (0.71) | 416 (0.53) | 111 (0.63) |
|  | oral contraceptives | 1 (0.00) | 0 (0.00) | 0 (0.00) | 0 (0.00) | 0 (0.00) | 0 (0.00) | 0 (0.00) | 0 (0.00) | 0 (0.00) | 0 (0.00) |
|  | hormone replacement therapy | 2959 (3.10) | 932 (4.12) | 288 (1.99) | 52 (1.72) | 131 (2.46) | 18 (1.62) | 855 (3.55) | 214 (4.21) | 2683 (3.43) | 829 (4.70) |
|  | anticonvulsants | 3012 (3.16) | 2880 (12.72) | 643 (4.44) | 413 (13.69) | 288 (5.40) | 370 (33.39) | 1239 (5.14) | 1027 (20.22) | 2807 (3.58) | 2151 (12.19) |
|  | cytostatic chemotherapy | 468 (0.49) | 145 (0.64) | 80 (0.55) | 18 (0.60) | 43 (0.81) | 9 (0.81) | 188 (0.78) | 37 (0.73) | 377 (0.48) | 111 (0.63) |
|  | radiotherapy | 22 (0.02) | 5 (0.02) | 3 (0.02) | 1 (0.03) | 2 (0.04) | 0 (0.00) | 11 (0.05) | 2 (0.04) | 17 (0.02) | 5 (0.03) |
|  | immunosuppressant medication | 1332 (1.40) | 375 (1.66) | 198 (1.37) | 45 (1.49) | 94 (1.76) | 18 (1.62) | 478 (1.98) | 118 (2.32) | 1148 (1.47) | 286 (1.62) |
|  | long-acting beta-agonist | 441 (0.46) | 162 (0.72) | 91 (0.63) | 28 (0.93) | 21 (0.39) | 6 (0.54) | 157 (0.65) | 39 (0.77) | 339 (0.43) | 116 (0.66) |
|  | leukotriene receptor antagonists | 835 (0.88) | 266 (1.18) | 118 (0.81) | 20 (0.66) | 59 (1.11) | 7 (0.63) | 241 (1.00) | 68 (1.34) | 738 (0.94) | 232 (1.31) |
|  | inhaled glucocorticoids | 1624 (1.70) | 507 (2.24) | 227 (1.57) | 52 (1.72) | 115 (2.16) | 20 (1.81) | 538 (2.23) | 110 (2.17) | 1386 (1.77) | 418 (2.37) |
|  | Number of inpatient contacts, mean (SD) | 0.76 (1.54) | 1.17 (2.12) | 0.82 (1.72) | 1.76 (2.90) | 0.98 (1.86) | 1.81 (3.14) | 0.99 (1.94) | 1.54 (2.62) | 0.78 (1.56) | 1.21 (2.19) |
|  | Number of outpatient contacts, mean (SD) | 75.20 (70.75) | 99.05 (83.96) | 75.28 (74.17) | 88.04 (92.51) | 84.14 (82.05) | 98.02 (121.29) | 90.51 (83.91) | 118.15 (95.61) | 77.33 (70.13) | 103.63 (79.96) |
| 5 | Region of permanent residency, n (%) |  | | | | | | | | | |
|  | Prague region | 108684 (13.06) | 30042 (16.04) | 13938 (12.63) | 3493 (15.73) | 4785 (12.61) | 1081 (14.18) | 26684 (13.47) | 7477 (18.76) | 92712 (13.17) | 24242 (16.14) |
|  | Central-Bohemian region | 109564 (13.17) | 25026 (13.36) | 14165 (12.84) | 2665 (12.00) | 5004 (13.18) | 846 (11.09) | 25946 (13.10) | 5486 (13.76) | 93209 (13.24) | 20477 (13.63) |
|  | South-Bohemian region | 46871 (5.63) | 10099 (5.39) | 6243 (5.66) | 1045 (4.71) | 2143 (5.65) | 470 (6.16) | 11043 (5.58) | 2198 (5.51) | 39684 (5.64) | 8116 (5.40) |
|  | Plzen region | 43307 (5.20) | 10701 (5.71) | 5824 (5.28) | 1116 (5.03) | 2010 (5.30) | 414 (5.43) | 10290 (5.20) | 2271 (5.70) | 36300 (5.16) | 8810 (5.87) |
|  | Karlovy Vary region | 14554 (1.75) | 3787 (2.02) | 1969 (1.78) | 408 (1.84) | 653 (1.72) | 165 (2.16) | 3609 (1.82) | 644 (1.62) | 12332 (1.75) | 3209 (2.14) |
|  | Usti region | 57405 (6.90) | 13193 (7.04) | 7680 (6.96) | 1725 (7.77) | 2601 (6.85) | 575 (7.54) | 13829 (6.98) | 2974 (7.46) | 48915 (6.95) | 10343 (6.89) |
|  | Liberec region | 31523 (3.79) | 7176 (3.83) | 4288 (3.89) | 765 (3.45) | 1439 (3.79) | 215 (2.82) | 7409 (3.74) | 1365 (3.42) | 26632 (3.78) | 5969 (3.97) |
|  | Hradec Kralove region | 40536 (4.87) | 8211 (4.38) | 5288 (4.79) | 878 (3.95) | 1838 (4.84) | 270 (3.54) | 9633 (4.86) | 1452 (3.64) | 34281 (4.87) | 6778 (4.51) |
|  | Pardubice region | 40354 (4.85) | 8637 (4.61) | 5399 (4.89) | 919 (4.14) | 1835 (4.83) | 322 (4.22) | 9349 (4.72) | 2152 (5.40) | 33894 (4.82) | 6804 (4.53) |
|  | Vysocina region | 36902 (4.43) | 7451 (3.98) | 4889 (4.43) | 784 (3.53) | 1684 (4.44) | 386 (5.06) | 8374 (4.23) | 1903 (4.77) | 31068 (4.41) | 5673 (3.78) |
|  | South-Moravian region | 102256 (12.29) | 20140 (10.75) | 13383 (12.13) | 2201 (9.91) | 4710 (12.41) | 892 (11.70) | 24081 (12.16) | 4417 (11.08) | 86239 (12.25) | 15914 (10.59) |
|  | Olomouc region | 52576 (6.32) | 12519 (6.68) | 7214 (6.54) | 1492 (6.72) | 2425 (6.39) | 484 (6.35) | 12545 (6.33) | 2371 (5.95) | 44160 (6.27) | 10183 (6.78) |
|  | Zlin region | 50104 (6.02) | 10315 (5.51) | 6720 (6.09) | 1600 (7.21) | 2286 (6.02) | 508 (6.66) | 11853 (5.98) | 1564 (3.92) | 41924 (5.96) | 8152 (5.43) |
|  | Moravian-Silesian region | 97553 (11.72) | 20023 (10.69) | 13322 (12.07) | 3114 (14.02) | 4542 (11.97) | 998 (13.09) | 23393 (11.81) | 3586 (9.00) | 82491 (11.72) | 15540 (10.35) |
|  | Abroad | 46 (0.01) | 1 (0.00) | 12 (0.01) | 0 (0.00) | 3 (0.01) | 0 (0.00) | 9 (0.00) | 0 (0.00) | 27 (0.00) | 1 (0.00) |
|  | Prescription medications, n (%) |  | | | | | | | | | |
|  | antihypertensives | 114780 (13.79) | 36218 (19.33) | 16372 (14.84) | 4105 (18.49) | 6292 (16.58) | 1235 (16.19) | 34037 (17.19) | 8570 (21.50) | 99618 (14.15) | 29300 (19.51) |
|  | aspirin | 36435 (4.38) | 9250 (4.94) | 5461 (4.95) | 1095 (4.93) | 2465 (6.49) | 479 (6.28) | 12078 (6.10) | 2483 (6.23) | 29063 (4.13) | 6817 (4.54) |
|  | statins | 77571 (9.32) | 18802 (10.04) | 11237 (10.18) | 1871 (8.43) | 5100 (13.44) | 871 (11.42) | 25791 (13.02) | 5324 (13.36) | 62360 (8.86) | 14082 (9.37) |
|  | antithrombotic agents | 137484 (16.52) | 41453 (22.13) | 19391 (17.57) | 4864 (21.90) | 8001 (21.08) | 1671 (21.91) | 42674 (21.55) | 10213 (25.62) | 116491 (16.55) | 32755 (21.81) |
|  | non-steroidal anti-inflammatory medications | 156488 (18.80) | 47647 (25.44) | 21449 (19.44) | 5153 (23.21) | 8512 (22.42) | 1354 (17.76) | 46889 (23.68) | 11875 (29.79) | 134281 (19.08) | 38548 (25.66) |
|  | bisphosphonates | 3489 (0.42) | 1024 (0.55) | 348 (0.32) | 51 (0.23) | 233 (0.61) | 32 (0.42) | 1350 (0.68) | 289 (0.73) | 3034 (0.43) | 804 (0.54) |
|  | oral contraceptives | 5 (0.00) | 1 (0.00) | 1 (0.00) | 0 (0.00) | 0 (0.00) | 0 (0.00) | 1 (0.00) | 0 (0.00) | 4 (0.00) | 1 (0.00) |
|  | hormone replacement therapy | 28623 (3.44) | 8259 (4.41) | 2717 (2.46) | 530 (2.39) | 1113 (2.93) | 212 (2.78) | 8263 (4.17) | 1943 (4.87) | 26385 (3.75) | 7134 (4.75) |
|  | anticonvulsants | 22424 (2.69) | 21756 (11.61) | 4388 (3.98) | 2859 (12.88) | 1851 (4.88) | 2346 (30.76) | 9485 (4.79) | 7644 (19.18) | 21724 (3.09) | 16581 (11.04) |
|  | cytostatic chemotherapy | 3803 (0.46) | 932 (0.50) | 681 (0.62) | 80 (0.36) | 282 (0.74) | 33 (0.43) | 1442 (0.73) | 234 (0.59) | 3271 (0.46) | 744 (0.50) |
|  | radiotherapy | 209 (0.03) | 35 (0.02) | 36 (0.03) | 2 (0.01) | 11 (0.03) | 2 (0.03) | 78 (0.04) | 7 (0.02) | 196 (0.03) | 30 (0.02) |
|  | immunosuppressant medication | 11149 (1.34) | 3126 (1.67) | 1703 (1.54) | 246 (1.11) | 669 (1.76) | 89 (1.17) | 3656 (1.85) | 829 (2.08) | 9716 (1.38) | 2518 (1.68) |
|  | long-acting beta-agonist | 3014 (0.36) | 944 (0.50) | 517 (0.47) | 143 (0.64) | 220 (0.58) | 47 (0.62) | 1049 (0.53) | 247 (0.62) | 2624 (0.37) | 701 (0.47) |
|  | leukotriene receptor antagonists | 7651 (0.92) | 2265 (1.21) | 1029 (0.93) | 152 (0.68) | 387 (1.02) | 52 (0.68) | 1983 (1.00) | 520 (1.30) | 6927 (0.98) | 1935 (1.29) |
|  | inhaled glucocorticoids | 13393 (1.61) | 4083 (2.18) | 1873 (1.70) | 339 (1.53) | 698 (1.84) | 110 (1.44) | 4060 (2.05) | 925 (2.32) | 11949 (1.70) | 3427 (2.28) |
|  | Number of inpatient contacts, mean (SD) | 0.67 (1.36) | 1.00 (1.88) | 0.73 (1.57) | 1.54 (2.71) | 0.84 (1.72) | 1.67 (3.06) | 0.85 (1.66) | 1.26 (2.29) | 0.69 (1.40) | 1.05 (1.96) |
|  | Number of outpatient contacts, mean (SD) | 77.43 (71.68) | 99.85 (88.36) | 78.51 (77.96) | 91.86 (99.47) | 87.66 (86.26) | 111.18 (166.53) | 93.23 (87.97) | 118.42 (99.87) | 79.94 (74.76) | 103.09 (80.74) |

The results are presented as absolute numbers (n) with proportions (%) and means with standard deviations (SD). The time frames for epochs were: (1) 1^st^ March 2020-30^th^ September 2020 for epoch 1, (2) 1^st^ October 2020-26^th^ December 2020 for epoch 2, (3) 27^th^ December 2020-31^st^ March 2021 for epoch 3, (4) 1^st^ April 2021-31^st^ October 2021 for epoch 4, and (5) 1^st^ November 2021-29^th^ February 2022 for epoch 5. “Exposed” and “unexposed” refer to people with the respective mental disorder and their matched counterparts without that mental disorder, respectively. The International Classification of Diseases 10^th^ Revision (ICD-10) diagnostic codes were (1) F10-F19, F20-F29, F30-F39, F40-F48 for any mental disorder, (2) F10-F19 for substance use disorders, (3) F20-F29 for psychotic disorders, (4) F30-F39 for affective disorders, and (5) F40-F48 for anxiety disorders.
