# Supplementary material for: Deaths with COVID-19 and from all-causes following first-ever SARS-CoV-2 infection in individuals with preexisting mental disorders: A national cohort study from Czechia
Source: PLoS Med. 2024 Jul 15;21(7):e1004422. doi: 10.1371/journal.pmed.1004422 (PMC11285938; doi:10.1371/journal.pmed.1004422)
Supplement: S8 Table — (DOCX) [file pmed.1004422.s010.docx]

Supplementary Table 8 Risk of death with COVID-19 up to 28 days in people with pre-existing mental disorders

| Cohort | Epoch | diagnosed | | | | diagnosed and treated | | | |
| --- | --- | --- | --- | --- | --- | --- | --- | --- | --- |
|  |  | aHR^*^  (95% CI) | *p*-value | faHR^†^  (95% CI) | *p*-value | aHR  (95% CI) | *p*-value | faHR  (95% CI) | *p*-value |
| Any mental disorder | 1 | 1.04 (0.79, 1.38) | 0.770 | 0.96 (0.70, 1.31) | 0.802 | 1.17 (0.81, 1.70) | 0.407 | 0.97 (0.61, 1.55) | 0.911 |
|  | 2 | 1.05 (0.99, 1.12) | 0.100 | 1.00 (0.94, 1.06) | 0.904 | 1.14 (1.06, 1.23) | <0.001 | 1.01 (0.93, 1.09) | 0.871 |
|  | 3 | 1.07 (1.02, 1.13) | 0.008 | 1.01 (0.95, 1.06) | 0.782 | 1.10 (1.03, 1.17) | 0.002 | 0.97 (0.91, 1.04) | 0.417 |
|  | 4 | 1.10 (0.96, 1.26) | 0.152 | 1.06 (0.92, 1.22) | 0.430 | 1.13 (0.96, 1.32) | 0.144 | 0.97 (0.81, 1.16) | 0.712 |
|  | 5 | 1.05 (0.97, 1.13) | 0.224 | 0.97 (0.90, 1.06) | 0.526 | 1.14 (1.04, 1.24) | 0.004 | 0.96 (0.87, 1.06) | 0.437 |
| Substance use disorders | 1 | NA | NA | NA | NA | NA | NA | NA | NA |
|  | 2 | 1.11 (0.94, 1.30) | 0.225 | 0.99 (0.83, 1.17) | 0.884 | 1.30 (1.08, 1.56) | 0.005 | 1.10 (0.90, 1.36) | 0.357 |
|  | 3 | 1.36 (1.18, 1.56) | <0.001 | 1.23 (1.06, 1.43) | 0.006 | 1.48 (1.25, 1.74) | <0.001 | 1.24 (1.02, 1.50) | 0.030 |
|  | 4 | 1.74 (1.26, 2.40) | <0.001 | 1.48 (1.02, 2.14) | 0.038 | 1.96 (1.29, 2.96) | 0.002 | 1.62 (0.96, 2.75) | 0.072 |
|  | 5 | 1.30 (1.08, 1.58) | 0.006 | 1.10 (0.89, 1.35) | 0.395 | 1.70 (1.36, 2.13) | <0.001 | 1.28 (0.98, 1.66) | 0.067 |
| Psychotic disorders | 1 | NA | NA | NA | NA | NA | NA | NA | NA |
|  | 2 | 1.49 (1.26, 1.77) | <0.001 | 1.50 (1.25, 1.80) | <0.001 | 1.63 (1.36, 1.95) | <0.001 | 1.46 (1.18, 1.79) | <0.001 |
|  | 3 | 1.69 (1.44, 1.98) | <0.001 | 1.57 (1.32, 1.86) | <0.001 | 1.88 (1.59, 2.23) | <0.001 | 1.72 (1.43, 2.08) | <0.001 |
|  | 4 | 1.87 (1.27, 2.74) | 0.002 | 1.61 (1.03, 2.51) | 0.036 | 2.08 (1.35, 3.20) | <0.001 | 1.93 (1.12, 3.32) | 0.018 |
|  | 5 | 1.94 (1.54, 2.45) | <0.001 | 1.79 (1.38, 2.32) | <0.001 | 1.91 (1.50, 2.43) | <0.001 | 1.53 (1.15, 2.03) | 0.003 |
| Affective disorders | 1 | 1.86 (1.18, 2.94) | 0.008 | 1.75 (0.96, 3.19) | 0.069 | 2.00 (1.15, 3.49) | 0.014 | 1.94 (0.86, 4.37) | 0.108 |
|  | 2 | 1.11 (1.00, 1.24) | 0.051 | 1.06 (0.95, 1.18) | 0.306 | 1.15 (1.03, 1.29) | 0.014 | 1.04 (0.92, 1.18) | 0.523 |
|  | 3 | 1.02 (0.92, 1.12) | 0.744 | 0.97 (0.88, 1.07) | 0.544 | 1.01 (0.92, 1.12) | 0.795 | 0.88 (0.79, 0.98) | 0.019 |
|  | 4 | 0.96 (0.75, 1.23) | 0.756 | 0.90 (0.70, 1.17) | 0.445 | 0.98 (0.75, 1.29) | 0.912 | 0.84 (0.61, 1.15) | 0.265 |
|  | 5 | 1.08 (0.94, 1.24) | 0.276 | 1.02 (0.88, 1.18) | 0.814 | 1.16 (1.00, 1.34) | 0.046 | 1.05 (0.89, 1.23) | 0.545 |
| Anxiety disorders | 1 | 0.89 (0.63, 1.26) | 0.512 | 0.75 (0.51, 1.11) | 0.155 | 1.23 (0.80, 1.91) | 0.348 | 1.03 (0.61, 1.72) | 0.918 |
|  | 2 | 0.93 (0.87, 1.01) | 0.079 | 0.88 (0.81, 0.95) | 0.001 | 1.01 (0.92, 1.10) | 0.889 | 0.89 (0.81, 0.98) | 0.016 |
|  | 3 | 0.96 (0.90, 1.02) | 0.198 | 0.89 (0.83, 0.95) | <0.001 | 0.97 (0.90, 1.04) | 0.374 | 0.85 (0.78, 0.92) | <0.001 |
|  | 4 | 0.94 (0.79, 1.12) | 0.476 | 0.92 (0.76, 1.10) | 0.343 | 1.03 (0.85, 1.26) | 0.734 | 0.90 (0.72, 1.12) | 0.347 |
|  | 5 | 0.90 (0.81, 0.99) | 0.027 | 0.82 (0.74, 0.90) | <0.001 | 0.97 (0.87, 1.08) | 0.567 | 0.78 (0.69, 0.88) | <0.001 |

* “Adjusted hazard ratios”: models were adjusted for matching variables.

† “Fully adjusted hazard ratios”: models were adjusted for matching variables and all additional confounders.

NA denotes situations when the models could not be reliably fit. All results are expressed as hazard ratios with 95% confidence intervals. The time frames for epochs were: (1) 1st March 2020-30th September 2020 for epoch 1, (2) 1st October 2020-26th December 2020 for epoch 2, (3) 27th December 2020-31st March 2021 for epoch 3, (4) 1st April 2021-31st October 2021 for epoch 4, and (5) 1st November 2021-29th February 2022 for epoch 5. “Diagnosed” refers to cases ascertained by diagnosis per the International Classification of Diseases 10th Revision (ICD-10) diagnostic codes: (1) F10-F19, F20-F29, F30-F39, F40-F48 for any mental disorder, (2) F10-F19 for substance use disorders, (3) F20-F29 for psychotic disorders, (4) F30-F39 for affective disorders, and (5) F40-F48 for anxiety disorders. “Diagnosed and treated” refers to cases ascertained by diagnosis per the above ICD-10 codes coupled with prescription for anxiolytics/hypnotics/sedatives (N05B, N05C), (2) antidepressants (N06A), (3) antipsychotics (N05A) or (4) stimulants (N06B) per the Anatomical Therapeutic Chemical (ATC) classification codes.
