# Supplementary material for: Deaths with COVID-19 and from all-causes following first-ever SARS-CoV-2 infection in individuals with preexisting mental disorders: A national cohort study from Czechia
Source: PLoS Med. 2024 Jul 15;21(7):e1004422. doi: 10.1371/journal.pmed.1004422 (PMC11285938; doi:10.1371/journal.pmed.1004422)
Supplement: S9 Table — (DOCX) [file pmed.1004422.s011.docx]

Supplementary Table 9 Risk of death with COVID-19 up to 60 days in people with pre-existing mental disorders

| Cohort | Epoch | diagnosed | | | | diagnosed and treated | | | |
| --- | --- | --- | --- | --- | --- | --- | --- | --- | --- |
|  |  | aHR^*^  (95% CI) | *p*-value | faHR^†^  (95% CI) | *p*-value | aHR  (95% CI) | *p*-value | faHR  (95% CI) | *p*-value |
| Any mental disorder | 1 | 1.06 (0.81, 1.38) | 0.673 | 0.98 (0.73, 1.31) | 0.878 | 1.23 (0.86, 1.75) | 0.254 | 1.06 (0.70, 1.63) | 0.774 |
|  | 2 | 1.05 (0.99, 1.11) | 0.121 | 0.99 (0.93, 1.05) | 0.776 | 1.13 (1.05, 1.21) | <0.001 | 1.00 (0.92, 1.08) | 0.991 |
|  | 3 | 1.07 (1.02, 1.13) | 0.008 | 1.01 (0.96, 1.06) | 0.807 | 1.11 (1.05, 1.18) | <0.001 | 0.98 (0.92, 1.04) | 0.534 |
|  | 4 | 1.11 (0.97, 1.27) | 0.119 | 1.07 (0.93, 1.22) | 0.368 | 1.16 (0.99, 1.35) | 0.069 | 0.98 (0.83, 1.17) | 0.850 |
|  | 5 | 1.05 (0.97, 1.13) | 0.218 | 0.98 (0.91, 1.06) | 0.579 | 1.13 (1.04, 1.24) | 0.004 | 0.97 (0.88, 1.07) | 0.504 |
| Substance use disorders | 1 | NA | NA | NA | NA | NA | NA | NA | NA |
|  | 2 | 1.12 (0.95, 1.31) | 0.175 | 1.00 (0.85, 1.19) | 0.963 | 1.30 (1.08, 1.55) | 0.004 | 1.11 (0.91, 1.35) | 0.319 |
|  | 3 | 1.33 (1.16, 1.52) | <0.001 | 1.21 (1.04, 1.40) | 0.011 | 1.46 (1.24, 1.71) | <0.001 | 1.22 (1.02, 1.48) | 0.033 |
|  | 4 | 1.67 (1.22, 2.30) | 0.002 | 1.37 (0.96, 1.97) | 0.084 | 1.99 (1.32, 2.99) | <0.001 | 1.60 (0.95, 2.69) | 0.077 |
|  | 5 | 1.32 (1.10, 1.58) | 0.003 | 1.13 (0.92, 1.38) | 0.238 | 1.72 (1.39, 2.14) | <0.001 | 1.29 (1.00, 1.66) | 0.049 |
| Psychotic disorders | 1 | NA | NA | NA | NA | NA | NA | NA | NA |
|  | 2 | 1.51 (1.28, 1.77) | <0.001 | 1.50 (1.25, 1.79) | <0.001 | 1.64 (1.38, 1.95) | <0.001 | 1.46 (1.20, 1.79) | <0.001 |
|  | 3 | 1.76 (1.51, 2.05) | <0.001 | 1.64 (1.39, 1.93) | <0.001 | 1.96 (1.66, 2.30) | <0.001 | 1.79 (1.49, 2.15) | <0.001 |
|  | 4 | 1.85 (1.27, 2.70) | 0.001 | 1.65 (1.07, 2.55) | 0.024 | 2.09 (1.36, 3.19) | <0.001 | 1.93 (1.14, 3.27) | 0.014 |
|  | 5 | 1.90 (1.51, 2.39) | <0.001 | 1.72 (1.33, 2.22) | <0.001 | 1.85 (1.46, 2.34) | <0.001 | 1.49 (1.13, 1.97) | 0.005 |
| Affective disorders | 1 | 1.71 (1.09, 2.67) | 0.020 | 1.74 (0.97, 3.11) | 0.062 | 1.82 (1.06, 3.14) | 0.031 | 1.75 (0.81, 3.80) | 0.156 |
|  | 2 | 1.11 (1.00, 1.23) | 0.043 | 1.06 (0.95, 1.18) | 0.301 | 1.14 (1.02, 1.27) | 0.019 | 1.03 (0.92, 1.16) | 0.606 |
|  | 3 | 1.01 (0.92, 1.11) | 0.833 | 0.96 (0.87, 1.06) | 0.437 | 1.02 (0.92, 1.12) | 0.750 | 0.88 (0.79, 0.98) | 0.021 |
|  | 4 | 0.94 (0.74, 1.20) | 0.628 | 0.87 (0.67, 1.12) | 0.277 | 0.97 (0.74, 1.28) | 0.847 | 0.82 (0.60, 1.12) | 0.211 |
|  | 5 | 1.10 (0.96, 1.26) | 0.172 | 1.03 (0.89, 1.19) | 0.674 | 1.18 (1.03, 1.36) | 0.019 | 1.06 (0.91, 1.24) | 0.477 |
| Anxiety disorders | 1 | 0.92 (0.66, 1.27) | 0.607 | 0.80 (0.55, 1.16) | 0.244 | 1.28 (0.85, 1.93) | 0.230 | 1.08 (0.67, 1.74) | 0.751 |
|  | 2 | 0.92 (0.86, 0.99) | 0.030 | 0.87 (0.80, 0.93) | <0.001 | 1.00 (0.91, 1.09) | 0.929 | 0.88 (0.80, 0.96) | 0.006 |
|  | 3 | 0.96 (0.90, 1.02) | 0.157 | 0.89 (0.83, 0.95) | <0.001 | 0.97 (0.90, 1.04) | 0.346 | 0.84 (0.78, 0.91) | <0.001 |
|  | 4 | 0.96 (0.81, 1.13) | 0.615 | 0.93 (0.78, 1.10) | 0.401 | 1.08 (0.90, 1.31) | 0.401 | 0.94 (0.76, 1.16) | 0.548 |
|  | 5 | 0.91 (0.82, 0.99) | 0.037 | 0.83 (0.75, 0.91) | <0.001 | 0.98 (0.88, 1.09) | 0.709 | 0.80 (0.71, 0.90) | <0.001 |

* “Adjusted hazard ratios”: models were adjusted for matching variables.

† “Fully adjusted hazard ratios”: models were adjusted for matching variables and all additional confounders.

NA denotes situations when the models could not be reliably fit. All results are expressed as hazard ratios with 95% confidence intervals. The time frames for epochs were: (1) 1st March 2020-30th September 2020 for epoch 1, (2) 1st October 2020-26th December 2020 for epoch 2, (3) 27th December 2020-31st March 2021 for epoch 3, (4) 1st April 2021-31st October 2021 for epoch 4, and (5) 1st November 2021-29th February 2022 for epoch 5. “Diagnosed” refers to cases ascertained by diagnosis per the International Classification of Diseases 10th Revision (ICD-10) diagnostic codes: (1) F10-F19, F20-F29, F30-F39, F40-F48 for any mental disorder, (2) F10-F19 for substance use disorders, (3) F20-F29 for psychotic disorders, (4) F30-F39 for affective disorders, and (5) F40-F48 for anxiety disorders. “Diagnosed and treated” refers to cases ascertained by diagnosis per the above ICD-10 codes coupled with prescription for anxiolytics/hypnotics/sedatives (N05B, N05C), (2) antidepressants (N06A), (3) antipsychotics (N05A) or (4) stimulants (N06B) per the Anatomical Therapeutic Chemical (ATC) classification codes.
