# Supplementary material for: Deaths with COVID-19 and from all-causes following first-ever SARS-CoV-2 infection in individuals with preexisting mental disorders: A national cohort study from Czechia
Source: PLoS Med. 2024 Jul 15;21(7):e1004422. doi: 10.1371/journal.pmed.1004422 (PMC11285938; doi:10.1371/journal.pmed.1004422)
Supplement: S10 Table — (DOCX) [file pmed.1004422.s012.docx]

Supplementary Table 10 Absolute risk of death with COVID-19 in people with pre-existing mental disorders

| Cohort | Epoch | Death with COVID-19 up to 28 days | | | | Death with COVID-19 up to 60 days | | | |
| --- | --- | --- | --- | --- | --- | --- | --- | --- | --- |
|  |  | diagnosed | | diagnosed and treated | | diagnosed | | diagnosed and treated | |
|  |  | unexposed,  n (%) | exposed,  n (%) | unexposed,  n (%) | exposed,  n (%) | unexposed,  n (%) | exposed,  n (%) | unexposed,  n (%) | exposed,  n (%) |
| Any mental disorder | 1 | 218 (0.74) | 72 (0.99) | 96 (0.51) | 57 (1.11) | 241 (0.82) | 80 (1.10) | 104 (0.55) | 64 (1.25) |
|  | 2 | 5564 (1.72) | 1387 (1.90) | 2717 (1.27) | 1235 (2.16) | 5926 (1.84) | 1471 (2.02) | 2912 (1.37) | 1307 (2.28) |
|  | 3 | 7463 (1.68) | 1820 (1.83) | 4460 (1.49) | 1573 (2.05) | 7983 (1.80) | 1945 (1.96) | 4776 (1.59) | 1681 (2.19) |
|  | 4 | 999 (1.05) | 293 (1.29) | 582 (0.94) | 239 (1.46) | 1059 (1.11) | 313 (1.38) | 614 (0.99) | 254 (1.56) |
|  | 5 | 3421 (0.41) | 871 (0.46) | 1955 (0.36) | 760 (0.56) | 3637 (0.44) | 927 (0.49) | 2091 (0.38) | 807 (0.60) |
| Substance use disorders | 1 | 39 (1.06) | 10 (1.27) | 10 (0.53) | 7 (1.61) | 45 (1.23) | 11 (1.39) | 10 (0.53) | 8 (1.84) |
|  | 2 | 823 (2.02) | 179 (2.19) | 577 (2.14) | 150 (2.76) | 879 (2.16) | 193 (2.37) | 616 (2.28) | 159 (2.92) |
|  | 3 | 960 (1.51) | 258 (2.02) | 634 (1.59) | 189 (2.35) | 1030 (1.62) | 272 (2.13) | 683 (1.71) | 200 (2.49) |
|  | 4 | 142 (0.98) | 53 (1.76) | 87 (1.05) | 35 (1.95) | 152 (1.05) | 54 (1.79) | 91 (1.09) | 36 (2.01) |
|  | 5 | 531 (0.48) | 140 (0.63) | 308 (0.47) | 108 (0.81) | 570 (0.52) | 152 (0.68) | 331 (0.50) | 117 (0.88) |
| Psychotic disorders | 1 | 17 (1.33) | 7 (2.58) | 7 (0.65) | 5 (2.02) | 18 (1.41) | 10 (3.69) | 7 (0.65) | 8 (3.24) |
|  | 2 | 628 (2.92) | 182 (4.23) | 524 (2.69) | 167 (4.27) | 673 (3.13) | 195 (4.53) | 566 (2.90) | 180 (4.60) |
|  | 3 | 627 (2.33) | 210 (3.89) | 516 (2.10) | 194 (3.90) | 669 (2.49) | 232 (4.29) | 548 (2.23) | 214 (4.31) |
|  | 4 | 96 (1.80) | 38 (3.43) | 69 (1.46) | 32 (3.18) | 101 (1.89) | 40 (3.61) | 71 (1.50) | 33 (3.28) |
|  | 5 | 274 (0.72) | 102 (1.34) | 247 (0.71) | 94 (1.35) | 289 (0.76) | 105 (1.38) | 262 (0.76) | 97 (1.39) |
| Affective disorders | 1 | 67 (0.88) | 31 (1.88) | 38 (0.61) | 28 (1.90) | 75 (0.99) | 31 (1.88) | 41 (0.66) | 28 (1.90) |
|  | 2 | 1930 (2.22) | 429 (2.47) | 1432 (1.87) | 414 (2.56) | 2039 (2.35) | 454 (2.61) | 1527 (2.00) | 438 (2.70) |
|  | 3 | 2469 (2.17) | 517 (2.26) | 2147 (2.11) | 495 (2.34) | 2660 (2.33) | 553 (2.41) | 2304 (2.27) | 529 (2.50) |
|  | 4 | 376 (1.56) | 83 (1.63) | 264 (1.29) | 72 (1.58) | 400 (1.66) | 86 (1.69) | 275 (1.35) | 73 (1.60) |
|  | 5 | 1121 (0.57) | 254 (0.64) | 910 (0.53) | 245 (0.68) | 1185 (0.60) | 273 (0.68) | 961 (0.55) | 264 (0.73) |
| Anxiety disorders | 1 | 164 (0.66) | 43 (0.74) | 74 (0.46) | 37 (0.90) | 182 (0.73) | 49 (0.85) | 83 (0.52) | 42 (1.02) |
|  | 2 | 4153 (1.56) | 816 (1.46) | 2173 (1.20) | 748 (1.69) | 4428 (1.66) | 859 (1.54) | 2333 (1.29) | 785 (1.77) |
|  | 3 | 5531 (1.53) | 1118 (1.46) | 3476 (1.38) | 976 (1.63) | 5908 (1.63) | 1192 (1.56) | 3729 (1.48) | 1041 (1.74) |
|  | 4 | 724 (0.92) | 172 (0.97) | 439 (0.85) | 152 (1.18) | 769 (0.98) | 187 (1.06) | 465 (0.90) | 166 (1.29) |
|  | 5 | 2529 (0.36) | 512 (0.34) | 1528 (0.33) | 454 (0.42) | 2687 (0.38) | 551 (0.37) | 1626 (0.35) | 488 (0.45) |

The results are presented as absolute numbers (n) with proportions (%). The time frames for epochs were: (1) 1st March 2020-30th September 2020 for epoch 1, (2) 1st October 2020-26th December 2020 for epoch 2, (3) 27th December 2020-31st March 2021 for epoch 3, (4) 1st April 2021-31st October 2021 for epoch 4, and (5) 1st November 2021-29th February 2022 for epoch 5. “Diagnosed” refers to cases ascertained by diagnosis per the International Classification of Diseases 10th Revision (ICD-10) diagnostic codes: (1) F10-F19, F20-F29, F30-F39, F40-F48 for any mental disorder, (2) F10-F19 for substance use disorders, (3) F20-F29 for psychotic disorders, (4) F30-F39 for affective disorders, and (5) F40-F48 for anxiety disorders. “Diagnosed and treated” refers to cases ascertained by diagnosis per the above ICD-10 codes coupled with prescription for anxiolytics/hypnotics/sedatives (N05B, N05C), (2) antidepressants (N06A), (3) antipsychotics (N05A) or (4) stimulants (N06B) per the Anatomical Therapeutic Chemical (ATC) classification codes.
