# Supplementary material for: Deaths with COVID-19 and from all-causes following first-ever SARS-CoV-2 infection in individuals with preexisting mental disorders: A national cohort study from Czechia
Source: PLoS Med. 2024 Jul 15;21(7):e1004422. doi: 10.1371/journal.pmed.1004422 (PMC11285938; doi:10.1371/journal.pmed.1004422)
Supplement: S11 Table — (DOCX) [file pmed.1004422.s013.docx]

Supplementary Table 11 Risk of all-cause mortality up to 28 days in people with pre-existing mental disorders

| Cohort | Epoch | diagnosed | | | | diagnosed and treated | | | |
| --- | --- | --- | --- | --- | --- | --- | --- | --- | --- |
|  |  | aHR^*^  (95% CI) | *p*-value | faHR^†^  (95% CI) | *p*-value | aHR  (95% CI) | *p*-value | faHR  (95% CI) | *p*-value |
| Any mental disorder | 1 | 1.04 (0.84, 1.30) | 0.696 | 0.97 (0.77, 1.23) | 0.811 | 1.18 (0.88, 1.58) | 0.257 | 1.00 (0.71, 1.42) | 0.978 |
|  | 2 | 1.06 (1.01, 1.11) | 0.024 | 1.00 (0.95, 1.05) | 0.976 | 1.16 (1.10, 1.23) | <0.001 | 1.03 (0.96, 1.09) | 0.438 |
|  | 3 | 1.07 (1.02, 1.12) | 0.005 | 1.01 (0.96, 1.05) | 0.836 | 1.09 (1.03, 1.15) | 0.002 | 0.96 (0.91, 1.02) | 0.179 |
|  | 4 | 1.08 (0.96, 1.21) | 0.210 | 1.03 (0.91, 1.17) | 0.609 | 1.18 (1.03, 1.36) | 0.021 | 1.03 (0.88, 1.20) | 0.724 |
|  | 5 | 1.07 (1.01, 1.14) | 0.033 | 1.01 (0.94, 1.07) | 0.847 | 1.16 (1.08, 1.25) | <0.001 | 0.99 (0.91, 1.07) | 0.756 |
| Substance use disorders | 1 | NA | NA | NA | NA | NA | NA | NA | NA |
|  | 2 | 1.26 (1.11, 1.43) | <0.001 | 1.13 (0.99, 1.30) | 0.072 | 1.42 (1.23, 1.64) | <0.001 | 1.22 (1.03, 1.43) | 0.019 |
|  | 3 | 1.43 (1.27, 1.61) | <0.001 | 1.30 (1.14, 1.47) | <0.001 | 1.59 (1.38, 1.83) | <0.001 | 1.35 (1.15, 1.60) | <0.001 |
|  | 4 | 1.81 (1.40, 2.36) | <0.001 | 1.59 (1.19, 2.12) | 0.002 | 2.34 (1.67, 3.28) | <0.001 | 1.91 (1.25, 2.91) | 0.003 |
|  | 5 | 1.63 (1.41, 1.88) | <0.001 | 1.37 (1.17, 1.60) | <0.001 | 2.01 (1.68, 2.39) | <0.001 | 1.43 (1.17, 1.76) | <0.001 |
| Psychotic disorders | 1 | NA | NA | NA | NA | NA | NA | NA | NA |
|  | 2 | 1.55 (1.36, 1.77) | <0.001 | 1.53 (1.32, 1.77) | <0.001 | 1.67 (1.45, 1.92) | <0.001 | 1.46 (1.24, 1.72) | <0.001 |
|  | 3 | 1.58 (1.37, 1.82) | <0.001 | 1.43 (1.23, 1.66) | <0.001 | 1.82 (1.57, 2.12) | <0.001 | 1.66 (1.41, 1.97) | <0.001 |
|  | 4 | 1.86 (1.32, 2.60) | <0.001 | 1.60 (1.09, 2.34) | 0.016 | 2.05 (1.41, 2.99) | <0.001 | 1.99 (1.25, 3.16) | 0.004 |
|  | 5 | 2.00 (1.66, 2.41) | <0.001 | 1.84 (1.49, 2.26) | <0.001 | 1.99 (1.64, 2.43) | <0.001 | 1.67 (1.33, 2.09) | <0.001 |
| Affective disorders | 1 | 1.65 (1.16, 2.34) | 0.005 | 1.40 (0.93, 2.11) | 0.110 | 1.65 (1.09, 2.52) | 0.019 | 1.41 (0.83, 2.39) | 0.199 |
|  | 2 | 1.06 (0.97, 1.15) | 0.218 | 1.00 (0.91, 1.09) | 0.992 | 1.13 (1.03, 1.23) | 0.010 | 1.00 (0.91, 1.11) | 0.963 |
|  | 3 | 1.00 (0.92, 1.09) | 0.968 | 0.96 (0.88, 1.05) | 0.334 | 1.01 (0.92, 1.10) | 0.910 | 0.87 (0.79, 0.96) | 0.007 |
|  | 4 | 0.95 (0.77, 1.17) | 0.599 | 0.93 (0.75, 1.16) | 0.525 | 1.03 (0.81, 1.30) | 0.825 | 0.91 (0.70, 1.19) | 0.501 |
|  | 5 | 1.02 (0.91, 1.14) | 0.778 | 0.94 (0.84, 1.06) | 0.344 | 1.10 (0.97, 1.24) | 0.130 | 0.99 (0.86, 1.13) | 0.851 |
| Anxiety disorders | 1 | 0.88 (0.67, 1.16) | 0.366 | 0.80 (0.59, 1.07) | 0.134 | 1.12 (0.79, 1.58) | 0.530 | 1.02 (0.69, 1.51) | 0.907 |
|  | 2 | 0.92 (0.87, 0.98) | 0.010 | 0.87 (0.81, 0.92) | <0.001 | 1.01 (0.94, 1.08) | 0.887 | 0.87 (0.80, 0.94) | <0.001 |
|  | 3 | 0.94 (0.89, 1.00) | 0.049 | 0.88 (0.83, 0.93) | <0.001 | 0.95 (0.89, 1.02) | 0.148 | 0.83 (0.77, 0.89) | <0.001 |
|  | 4 | 0.88 (0.76, 1.02) | 0.095 | 0.84 (0.72, 0.99) | 0.036 | 1.03 (0.86, 1.22) | 0.767 | 0.88 (0.73, 1.07) | 0.200 |
|  | 5 | 0.90 (0.83, 0.97) | 0.008 | 0.83 (0.77, 0.90) | <0.001 | 0.98 (0.90, 1.08) | 0.735 | 0.81 (0.74, 0.90) | <0.001 |

* “Adjusted hazard ratios”: models were adjusted for matching variables.

† “Fully adjusted hazard ratios”: models were adjusted for matching variables and all additional confounders.

NA denotes situations when the models could not be reliably fit. All results are expressed as hazard ratios with 95% confidence intervals. The time frames for epochs were: (1) 1st March 2020-30th September 2020 for epoch 1, (2) 1st October 2020-26th December 2020 for epoch 2, (3) 27th December 2020-31st March 2021 for epoch 3, (4) 1st April 2021-31st October 2021 for epoch 4, and (5) 1st November 2021-29th February 2022 for epoch 5. “Diagnosed” refers to cases ascertained by diagnosis per the International Classification of Diseases 10th Revision (ICD-10) diagnostic codes: (1) F10-F19, F20-F29, F30-F39, F40-F48 for any mental disorder, (2) F10-F19 for substance use disorders, (3) F20-F29 for psychotic disorders, (4) F30-F39 for affective disorders, and (5) F40-F48 for anxiety disorders. “Diagnosed and treated” refers to cases ascertained by diagnosis per the above ICD-10 codes coupled with prescription for anxiolytics/hypnotics/sedatives (N05B, N05C), (2) antidepressants (N06A), (3) antipsychotics (N05A) or (4) stimulants (N06B) per the Anatomical Therapeutic Chemical (ATC) classification codes.
