# Supplementary material for: Deaths with COVID-19 and from all-causes following first-ever SARS-CoV-2 infection in individuals with preexisting mental disorders: A national cohort study from Czechia
Source: PLoS Med. 2024 Jul 15;21(7):e1004422. doi: 10.1371/journal.pmed.1004422 (PMC11285938; doi:10.1371/journal.pmed.1004422)
Supplement: S12 Table — (DOCX) [file pmed.1004422.s014.docx]

Supplementary Table 12 Risk of all-cause mortality up to 60 days in people with pre-existing mental disorders

| Cohort | Epoch | diagnosed | | | | diagnosed and treated | | | |
| --- | --- | --- | --- | --- | --- | --- | --- | --- | --- |
|  |  | aHR^*^  (95% CI) | *p*-value | faHR^†^  (95% CI) | *p*-value | aHR  (95% CI) | *p*-value | faHR  (95% CI) | *p*-value |
| Any mental disorder | 1 | 1.07 (0.87, 1.30) | 0.531 | 1.00 (0.81, 1.24) | 0.982 | 1.27 (0.98, 1.66) | 0.074 | 1.13 (0.83, 1.53) | 0.441 |
|  | 2 | 1.06 (1.02, 1.11) | 0.005 | 1.00 (0.96, 1.05) | 0.886 | 1.17 (1.11, 1.24) | <0.001 | 1.02 (0.96, 1.09) | 0.447 |
|  | 3 | 1.08 (1.03, 1.13) | <0.001 | 1.02 (0.97, 1.06) | 0.503 | 1.13 (1.07, 1.18) | <0.001 | 0.99 (0.94, 1.05) | 0.698 |
|  | 4 | 1.05 (0.94, 1.16) | 0.411 | 1.02 (0.91, 1.14) | 0.775 | 1.20 (1.06, 1.37) | 0.006 | 1.04 (0.90, 1.20) | 0.560 |
|  | 5 | 1.10 (1.04, 1.16) | 0.001 | 1.04 (0.98, 1.10) | 0.208 | 1.19 (1.12, 1.27) | <0.001 | 1.02 (0.95, 1.10) | 0.601 |
| Substance use disorders | 1 | NA | NA | NA | NA | NA | NA | NA | NA |
|  | 2 | 1.36 (1.21, 1.53) | <0.001 | 1.22 (1.08, 1.38) | 0.001 | 1.51 (1.33, 1.73) | <0.001 | 1.30 (1.12, 1.51) | <0.001 |
|  | 3 | 1.49 (1.34, 1.66) | <0.001 | 1.36 (1.21, 1.53) | <0.001 | 1.69 (1.48, 1.92) | <0.001 | 1.46 (1.26, 1.70) | <0.001 |
|  | 4 | 1.72 (1.35, 2.21) | <0.001 | 1.52 (1.16, 1.98) | 0.002 | 2.32 (1.70, 3.16) | <0.001 | 1.87 (1.28, 2.73) | 0.001 |
|  | 5 | 1.74 (1.53, 1.98) | <0.001 | 1.46 (1.27, 1.68) | <0.001 | 2.05 (1.75, 2.39) | <0.001 | 1.52 (1.27, 1.82) | <0.001 |
| Psychotic disorders | 1 | NA | NA | NA | NA | NA | NA | NA | NA |
|  | 2 | 1.57 (1.39, 1.77) | <0.001 | 1.52 (1.33, 1.74) | <0.001 | 1.75 (1.54, 1.99) | <0.001 | 1.53 (1.32, 1.78) | <0.001 |
|  | 3 | 1.73 (1.52, 1.96) | <0.001 | 1.57 (1.37, 1.80) | <0.001 | 1.99 (1.74, 2.29) | <0.001 | 1.80 (1.54, 2.10) | <0.001 |
|  | 4 | 1.78 (1.29, 2.45) | <0.001 | 1.55 (1.08, 2.23) | 0.017 | 1.80 (1.26, 2.58) | 0.001 | 1.74 (1.13, 2.67) | 0.012 |
|  | 5 | 1.88 (1.59, 2.23) | <0.001 | 1.72 (1.43, 2.07) | <0.001 | 1.97 (1.65, 2.36) | <0.001 | 1.65 (1.34, 2.03) | <0.001 |
| Affective disorders | 1 | 1.57 (1.13, 2.19) | 0.007 | 1.49 (1.01, 2.19) | 0.043 | 1.61 (1.08, 2.39) | 0.019 | 1.46 (0.90, 2.38) | 0.126 |
|  | 2 | 1.05 (0.97, 1.14) | 0.201 | 0.99 (0.91, 1.08) | 0.811 | 1.13 (1.03, 1.23) | 0.006 | 0.99 (0.90, 1.08) | 0.798 |
|  | 3 | 1.02 (0.94, 1.10) | 0.665 | 0.97 (0.89, 1.05) | 0.492 | 1.04 (0.96, 1.13) | 0.352 | 0.90 (0.83, 0.99) | 0.027 |
|  | 4 | 0.90 (0.74, 1.10) | 0.310 | 0.89 (0.72, 1.09) | 0.250 | 1.01 (0.81, 1.26) | 0.958 | 0.90 (0.70, 1.16) | 0.422 |
|  | 5 | 1.00 (0.90, 1.11) | 1.000 | 0.93 (0.83, 1.04) | 0.187 | 1.10 (0.98, 1.23) | 0.092 | 0.97 (0.86, 1.10) | 0.636 |
| Anxiety disorders | 1 | 0.88 (0.69, 1.13) | 0.322 | 0.84 (0.64, 1.10) | 0.196 | 1.19 (0.87, 1.63) | 0.274 | 1.11 (0.78, 1.58) | 0.563 |
|  | 2 | 0.94 (0.89, 0.99) | 0.021 | 0.87 (0.82, 0.93) | <0.001 | 1.01 (0.95, 1.08) | 0.677 | 0.87 (0.81, 0.94) | <0.001 |
|  | 3 | 0.95 (0.90, 1.00) | 0.067 | 0.88 (0.84, 0.93) | <0.001 | 0.97 (0.92, 1.03) | 0.393 | 0.84 (0.79, 0.90) | <0.001 |
|  | 4 | 0.88 (0.76, 1.01) | 0.064 | 0.85 (0.73, 0.98) | 0.026 | 1.03 (0.87, 1.20) | 0.760 | 0.90 (0.76, 1.08) | 0.255 |
|  | 5 | 0.92 (0.86, 0.99) | 0.023 | 0.86 (0.80, 0.92) | <0.001 | 1.01 (0.93, 1.09) | 0.828 | 0.84 (0.77, 0.92) | <0.001 |

* “Adjusted hazard ratios”: models were adjusted for matching variables.

† “Fully adjusted hazard ratios”: models were adjusted for matching variables and all additional confounders.

NA denotes situations when the models could not be reliably fit. All results are expressed as hazard ratios with 95% confidence intervals. The time frames for epochs were: (1) 1st March 2020-30th September 2020 for epoch 1, (2) 1st October 2020-26th December 2020 for epoch 2, (3) 27th December 2020-31st March 2021 for epoch 3, (4) 1st April 2021-31st October 2021 for epoch 4, and (5) 1st November 2021-29th February 2022 for epoch 5. “Diagnosed” refers to cases ascertained by diagnosis per the International Classification of Diseases 10th Revision (ICD-10) diagnostic codes: (1) F10-F19, F20-F29, F30-F39, F40-F48 for any mental disorder, (2) F10-F19 for substance use disorders, (3) F20-F29 for psychotic disorders, (4) F30-F39 for affective disorders, and (5) F40-F48 for anxiety disorders. “Diagnosed and treated” refers to cases ascertained by diagnosis per the above ICD-10 codes coupled with prescription for anxiolytics/hypnotics/sedatives (N05B, N05C), (2) antidepressants (N06A), (3) antipsychotics (N05A) or (4) stimulants (N06B) per the Anatomical Therapeutic Chemical (ATC) classification codes.
