# Supplementary material for: Deaths with COVID-19 and from all-causes following first-ever SARS-CoV-2 infection in individuals with preexisting mental disorders: A national cohort study from Czechia
Source: PLoS Med. 2024 Jul 15;21(7):e1004422. doi: 10.1371/journal.pmed.1004422 (PMC11285938; doi:10.1371/journal.pmed.1004422)
Supplement: S13 Table — (DOCX) [file pmed.1004422.s015.docx]

Supplementary Table 13 Absolute risk of all-cause mortality in people with pre-existing mental disorders

| Cohort | Epoch | All-cause mortality up to 28 days | | | | All-cause mortality up to 60 days | | | |
| --- | --- | --- | --- | --- | --- | --- | --- | --- | --- |
|  |  | diagnosed | | diagnosed and treated | | diagnosed | | diagnosed and treated | |
|  |  | unexposed,  n (%) | exposed,  n (%) | unexposed,  n (%) | exposed,  n (%) | unexposed,  n (%) | exposed,  n (%) | unexposed,  n (%) | exposed,  n (%) |
| Any mental disorder | 1 | 372 (1.26) | 120 (1.65) | 158 (0.84) | 88 (1.72) | 442 (1.50) | 145 (1.99) | 186 (0.99) | 108 (2.11) |
|  | 2 | 8683 (2.69) | 2191 (3.01) | 4062 (1.90) | 1936 (3.38) | 10178 (3.15) | 2599 (3.57) | 4754 (2.23) | 2293 (4.00) |
|  | 3 | 9761 (2.20) | 2383 (2.40) | 5717 (1.90) | 2055 (2.68) | 11117 (2.51) | 2756 (2.78) | 6452 (2.15) | 2382 (3.10) |
|  | 4 | 1387 (1.45) | 397 (1.75) | 766 (1.24) | 320 (1.96) | 1640 (1.72) | 461 (2.04) | 905 (1.46) | 377 (2.31) |
|  | 5 | 5256 (0.63) | 1375 (0.73) | 2916 (0.54) | 1169 (0.87) | 6382 (0.77) | 1720 (0.92) | 3493 (0.64) | 1443 (1.07) |
| Substance use disorders | 1 | 55 (1.50) | 18 (2.28) | 17 (0.91) | 12 (2.76) | 69 (1.88) | 21 (2.66) | 19 (1.02) | 15 (3.46) |
|  | 2 | 1205 (2.96) | 303 (3.71) | 858 (3.18) | 245 (4.50) | 1405 (3.45) | 379 (4.64) | 1001 (3.70) | 304 (5.59) |
|  | 3 | 1277 (2.01) | 370 (2.90) | 831 (2.08) | 271 (3.37) | 1474 (2.32) | 444 (3.48) | 950 (2.38) | 329 (4.09) |
|  | 4 | 210 (1.45) | 82 (2.72) | 113 (1.36) | 57 (3.18) | 249 (1.72) | 92 (3.05) | 135 (1.62) | 67 (3.73) |
|  | 5 | 785 (0.71) | 262 (1.18) | 444 (0.68) | 188 (1.42) | 956 (0.87) | 342 (1.54) | 558 (0.85) | 242 (1.82) |
| Psychotic disorders | 1 | 31 (2.43) | 13 (4.80) | 13 (1.20) | 10 (4.05) | 38 (2.98) | 18 (6.64) | 16 (1.48) | 15 (6.07) |
|  | 2 | 966 (4.50) | 293 (6.81) | 816 (4.18) | 271 (6.92) | 1146 (5.34) | 351 (8.16) | 946 (4.85) | 325 (8.30) |
|  | 3 | 844 (3.14) | 266 (4.92) | 659 (2.68) | 244 (4.91) | 950 (3.54) | 325 (6.01) | 740 (3.01) | 298 (6.00) |
|  | 4 | 124 (2.32) | 50 (4.51) | 91 (1.93) | 42 (4.17) | 145 (2.72) | 55 (4.96) | 108 (2.29) | 45 (4.47) |
|  | 5 | 410 (1.08) | 159 (2.08) | 368 (1.06) | 146 (2.09) | 522 (1.38) | 192 (2.52) | 442 (1.28) | 174 (2.49) |
| Affective disorders | 1 | 124 (1.63) | 49 (2.98) | 73 (1.17) | 41 (2.79) | 146 (1.92) | 54 (3.28) | 81 (1.30) | 45 (3.06) |
|  | 2 | 3099 (3.56) | 658 (3.78) | 2241 (2.93) | 634 (3.91) | 3656 (4.21) | 774 (4.45) | 2629 (3.44) | 748 (4.62) |
|  | 3 | 3244 (2.85) | 673 (2.94) | 2791 (2.75) | 647 (3.06) | 3727 (3.27) | 784 (3.42) | 3145 (3.10) | 754 (3.56) |
|  | 4 | 513 (2.13) | 112 (2.21) | 353 (1.73) | 96 (2.11) | 598 (2.48) | 127 (2.50) | 405 (1.98) | 109 (2.39) |
|  | 5 | 1751 (0.88) | 370 (0.93) | 1369 (0.79) | 351 (0.97) | 2152 (1.09) | 452 (1.13) | 1648 (0.95) | 428 (1.18) |
| Anxiety disorders | 1 | 268 (1.08) | 69 (1.19) | 125 (0.78) | 57 (1.38) | 326 (1.31) | 85 (1.47) | 147 (0.92) | 69 (1.67) |
|  | 2 | 6528 (2.45) | 1269 (2.28) | 3274 (1.81) | 1148 (2.60) | 7663 (2.88) | 1508 (2.70) | 3875 (2.14) | 1364 (3.08) |
|  | 3 | 7215 (1.99) | 1447 (1.89) | 4480 (1.78) | 1262 (2.11) | 8212 (2.27) | 1666 (2.18) | 5073 (2.01) | 1462 (2.44) |
|  | 4 | 1013 (1.29) | 226 (1.28) | 575 (1.11) | 195 (1.52) | 1193 (1.52) | 267 (1.51) | 691 (1.34) | 234 (1.82) |
|  | 5 | 3947 (0.56) | 806 (0.54) | 2329 (0.50) | 709 (0.65) | 4816 (0.68) | 1010 (0.67) | 2805 (0.60) | 880 (0.80) |

The results are presented as absolute numbers (n) with proportions (%). The time frames for epochs were: (1) 1st March 2020-30th September 2020 for epoch 1, (2) 1st October 2020-26th December 2020 for epoch 2, (3) 27th December 2020-31st March 2021 for epoch 3, (4) 1st April 2021-31st October 2021 for epoch 4, and (5) 1st November 2021-29th February 2022 for epoch 5. “Diagnosed” refers to cases ascertained by diagnosis per the International Classification of Diseases 10th Revision (ICD-10) diagnostic codes: (1) F10-F19, F20-F29, F30-F39, F40-F48 for any mental disorder, (2) F10-F19 for substance use disorders, (3) F20-F29 for psychotic disorders, (4) F30-F39 for affective disorders, and (5) F40-F48 for anxiety disorders. “Diagnosed and treated” refers to cases ascertained by diagnosis per the above ICD-10 codes coupled with prescription for anxiolytics/hypnotics/sedatives (N05B, N05C), (2) antidepressants (N06A), (3) antipsychotics (N05A) or (4) stimulants (N06B) per the Anatomical Therapeutic Chemical (ATC) classification codes.
