# Supplementary material for: Deaths with COVID-19 and from all-causes following first-ever SARS-CoV-2 infection in individuals with preexisting mental disorders: A national cohort study from Czechia
Source: PLoS Med. 2024 Jul 15;21(7):e1004422. doi: 10.1371/journal.pmed.1004422 (PMC11285938; doi:10.1371/journal.pmed.1004422)
Supplement: S14 Table — (DOCX) [file pmed.1004422.s016.docx]

Supplementary Table 14 Negative control exposure analyses

| Cohort | Epoch | Tests, n | Non-null tests, n^*^ | Non-null tests, %^*^ | Mean effect size^†^ |
| --- | --- | --- | --- | --- | --- |
| any mental disorder | 1 | 36 | 2 | 5.56 | 1.05 |
| any mental disorder | 2 | 40 | 13 | 32.50 | 0.83 |
| any mental disorder | 3 | 40 | 17 | 42.50 | 0.84 |
| any mental disorder | 4 | 40 | 1 | 2.50 | 0.76 |
| any mental disorder | 5 | 40 | 15 | 37.50 | 0.71 |
| substance use disorders | 1 | NA | NA | NA | NA |
| substance use disorders | 2 | 38 | 3 | 7.89 | 1.06 |
| substance use disorders | 3 | 40 | 3 | 7.50 | 0.95 |
| substance use disorders | 4 | 34 | 4 | 11.76 | 1.07 |
| substance use disorders | 5 | 40 | 4 | 10.00 | 0.88 |
| psychotic disorders | 1 | NA | NA | NA | NA |
| psychotic disorders | 2 | 37 | 2 | 5.41 | 1.04 |
| psychotic disorders | 3 | 40 | 6 | 15.00 | 0.88 |
| psychotic disorders | 4 | 20 | 0 | 0.00 | 0.59 |
| psychotic disorders | 5 | 40 | 1 | 2.50 | 1.05 |
| affective disorders | 1 | 24 | 0 | 0.00 | 2.28 |
| affective disorders | 2 | 40 | 7 | 17.50 | 0.88 |
| affective disorders | 3 | 40 | 7 | 17.50 | 0.82 |
| affective disorders | 4 | 38 | 6 | 15.79 | 0.91 |
| affective disorders | 5 | 40 | 3 | 7.50 | 0.90 |
| anxiety disorders | 1 | 36 | 0 | 0.00 | 1.21 |
| anxiety disorders | 2 | 40 | 10 | 25.00 | 0.92 |
| anxiety disorders | 3 | 40 | 15 | 37.50 | 0.81 |
| anxiety disorders | 4 | 38 | 0 | 0.00 | 0.84 |
| anxiety disorders | 5 | 40 | 16 | 40.00 | 0.70 |

* Refers to the number and proportion of non-null tests from all conducted negative control exposure tests.

† Refers to the averaged hazard ratios across all conducted negative control exposure tests.

NA denotes situations when the main models could not be reliably fit and thus the presence of potential collider bias was not examined. The theoretical maximum of 40 tests per one epoch-mental disorder combination reflects the fact that each mental disorder was defined using two approaches (i.e., International Classification of Diseases 10th Revision [ICD-10] diagnostic codes or ICD-10 codes coupled with Anatomical Therapeutic Chemical [ATC] classification codes), and there were five negative control exposures and four outcomes (i.e., deaths with COVID-19 and all-cause mortality occurring in 28 and 60 days following the infection). We note that the theoretical maximum was 40 tests per each epoch-mental disorder combination; however, some models could not be reliably fit, resulting in a lower number of performed tests. The time frames for epochs were: (1) 1st March 2020-30th September 2020 for epoch 1, (2) 1st October 2020-26th December 2020 for epoch 2, (3) 27th December 2020-31st March 2021 for epoch 3, (4) 1st April 2021-31st October 2021 for epoch 4, and (5) 1st November 2021-29th February 2022 for epoch 5. The ICD-10 diagnostic codes were (1) F10-F19, F20-F29, F30-F39, F40-F48 for any mental disorder, (2) F10-F19 for substance use disorders, (3) F20-F29 for psychotic disorders, (4) F30-F39 for affective disorders, and (5) F40-F48 for anxiety disorders. The considered psychopharmaceuticals per the ATC classification codes were (1) anxiolytics/hypnotics/sedatives (N05B, N05C), (2) antidepressants (N06A), (3) antipsychotics (N05A), and (4) stimulants (N06B).
