# Supplementary material for: Deaths with COVID-19 and from all-causes following first-ever SARS-CoV-2 infection in individuals with preexisting mental disorders: A national cohort study from Czechia
Source: PLoS Med. 2024 Jul 15;21(7):e1004422. doi: 10.1371/journal.pmed.1004422 (PMC11285938; doi:10.1371/journal.pmed.1004422)
Supplement: S15 Table — (DOCX) [file pmed.1004422.s017.docx]

Supplementary Table 15 E-values for models on deaths with COVID-19

| Cohort | Epoch | Up to 28 days | | Up to 60 days | |
| --- | --- | --- | --- | --- | --- |
|  |  | diagnosed | diagnosed and treated | diagnosed | diagnosed and treated |
| any mental disorder | 1 | NA | NA | NA | NA |
| any mental disorder | 2 | NA | NA | NA | NA |
| any mental disorder | 3 | NA | NA | NA | NA |
| any mental disorder | 4 | NA | NA | NA | NA |
| any mental disorder | 5 | NA | NA | NA | NA |
| substance use disorders | 1 | NA | NA | NA | NA |
| substance use disorders | 2 | NA | NA | NA | NA |
| substance use disorders | 3 | 1.76 | 1.78 | 1.71 | 1.75 |
| substance use disorders | 4 | 2.32 | NA | NA | NA |
| substance use disorders | 5 | NA | NA | NA | 1.9 |
| psychotic disorders | 1 | NA | NA | NA | NA |
| psychotic disorders | 2 | 2.36 | 2.27 | 2.36 | 2.29 |
| psychotic disorders | 3 | 2.52 | 2.84 | 2.66 | 2.98 |
| psychotic disorders | 4 | 2.6 | 3.26 | 2.68 | 3.27 |
| psychotic disorders | 5 | 2.98 | 2.42 | 2.84 | 2.35 |
| affective disorders | 1 | NA | NA | NA | NA |
| affective disorders | 2 | NA | NA | NA | NA |
| affective disorders | 3 | NA | 1.54 | NA | 1.52 |
| affective disorders | 4 | NA | NA | NA | NA |
| affective disorders | 5 | NA | NA | NA | NA |
| anxiety disorders | 1 | NA | NA | NA | NA |
| anxiety disorders | 2 | 1.53 | 1.5 | 1.58 | 1.54 |
| anxiety disorders | 3 | 1.5 | 1.65 | 1.51 | 1.66 |
| anxiety disorders | 4 | NA | NA | NA | NA |
| anxiety disorders | 5 | 1.75 | 1.88 | 1.7 | 1.81 |

We calculated E-values only for models that were not consistent with a null effect. The time frames for epochs were: (1) 1st March 2020-30th September 2020 for epoch 1, (2) 1st October 2020-26th December 2020 for epoch 2, (3) 27th December 2020-31st March 2021 for epoch 3, (4) 1st April 2021-31st October 2021 for epoch 4, and (5) 1st November 2021-29th February 2022 for epoch 5. “Diagnosed” refers to cases ascertained by diagnosis per the International Classification of Diseases 10th Revision (ICD-10) diagnostic codes: (1) F10-F19, F20-F29, F30-F39, F40-F48 for any mental disorder, (2) F10-F19 for substance use disorders, (3) F20-F29 for psychotic disorders, (4) F30-F39 for affective disorders, and (5) F40-F48 for anxiety disorders. “Diagnosed and treated” refers to cases ascertained by diagnosis per the above ICD-10 codes coupled with prescription for anxiolytics/hypnotics/sedatives (N05B, N05C), (2) antidepressants (N06A), (3) antipsychotics (N05A) or (4) stimulants (N06B) per the Anatomical Therapeutic Chemical (ATC) classification codes.
