# Supplementary material for: Deaths with COVID-19 and from all-causes following first-ever SARS-CoV-2 infection in individuals with preexisting mental disorders: A national cohort study from Czechia
Source: PLoS Med. 2024 Jul 15;21(7):e1004422. doi: 10.1371/journal.pmed.1004422 (PMC11285938; doi:10.1371/journal.pmed.1004422)
Supplement: Attachment — Submitted filename: Authors_reply.docx [file pmed.1004422.s020.docx]

**Responses to Editorial and Reviewer Comments**

We are extremely grateful to editors and reviewers for their time in appraising our manuscript. The comments and suggestions have helped us to improve our work. Below, we present a point-by-point list of editorial and reviewer comments alongside our responses. We submit two versions of the amended manuscript; one clean copy and a second annotated copy with tracked changes. The documents also include page and line numbers, which we refer to in the comments below.

**Editorial Comments**

**Point 1a:** “In line with the reviewer's comments, and given that you do not have the data, we feel that the lack of adjustment for health-related confounders such as body mass index as a limitation needs to be discussed in more detail.”

**Reply:** Thank you for this comment. From the most important previously identified clinical, sociodemographic, and behavioural confounders, we were unable to directly control for body mass index, smoking status, and socioeconomic status [1]. However, all these covariates are known to influence an individual’s overall health [2-7]. To consider overall health, we matched on a widely used comorbidity index and subsequently adjusted for the overall number of contacts with inpatient services and the overall number of contacts with outpatient services (disregarding contacts related to the exposure), and prescription medications, including antihypertensives (ATC code C02) and statins (ATC code C10AA) that would be administered for conditions commonly present in people who smoke or who are obese [8-11].

As demonstrated by VanderWeele using the causal diagram below, adjusting for a measured covariate C, even in the presence of an unmeasured covariate U, removes confounding between exposure A and outcome Y [12].

Correspondingly, while we did not directly control for body mass index, smoking status, and socioeconomic status, adjusting for overall health, represented by a comorbidity index, overall number of contacts with inpatient services and overall number of contacts with outpatient services, and prescription medications, likely contributed to substantial reduction of potential confounding due to these covariates. On the other hand, we cannot rule out that there are also unknown confounders that would influence our estimates. Consequently, the most plausible direction of bias seems to be the overestimation of true effects.

We have now elaborated on these in the Discussion as follows (page 11 line 497-509):

- “Second, we exactly-matched on key sociodemographic and clinical covariates and subsequently adjusted for a wide range of additional health-related confounders; however, we did not control for a number of previously identified influential clinical, sociodemographic, and behavioural confounders [1]. In particular, we had no information on body mass index, smoking status, and socioeconomic status *per se*; however, these are likely to influence an individual’s overall health [2-7], which we considered by controlling for a comorbidity index, overall number of contacts with inpatient and outpatient services, and prescription medications, including antihypertensives and statins that would be administered for conditions commonly present in people who smoke or who are obese [8-11]. These steps likely reduced the level of confounding due to these covariates; however, we cannot rule out that part of the detected associations is still due to residual confounding, both related to these known and directly unmeasured and potential unknown confounders, with the most plausible direction of bias being the overestimation of true effects.”.

Furthermore, we have now explicitly acknowledged that of part the detected associations might be due to residual confounding in the Abstract as follows (page 2 line 87-89):

- “While it cannot be ruled out that part of the detected associations is due to residual confounding, this excess mortality cannot be fully explained by lower vaccination uptake and more clinically-recorded physical comorbidities in these patient groups.”.

and in the Discussion as follows (page 12 line 531-535):

- “While we cannot rule out that part of the detected associations is due to residual confounding, the consistently increased vulnerability beyond vaccination uptake and clinically-recorded physical comorbidity aligns with existing evidence on fatal health inequalities in these patient groups and underlines the importance of implementing systemic efforts to fully reverse these.”.

**Point 1b:** “We also believe that the presentation of data from the Czech Republic is a strength of your study, and you may wish to include points about this aspect and mental health care in the Central and Eastern European region in the introduction and/or discussion.”

**Reply:** Thank you for this comment. We have now provided discussion of the Czech mental health care in the Methods as follows (page 4 line 170-178):

- “Mental health care in Central and Eastern European region relies on large psychiatric hospitals [13-15]. Considering Czechia in particular, more than 50% of its mental health budget is allocated to inpatient services [16], with the majority of inpatient care provided in outdated psychiatric hospitals [17]. However, Czechia has launched its mental health reform in 2013, with its initial main goals focusing on deinstitutionalization. This entails the expansion of community-based services, alongside a reduction in long-term inpatient beds and complemented by educational, destigmatization and other implementation programs aimed at improving the quality of care and overall quality of life of people with psychiatric conditions [15, 18].”.

**Reviewer #1**

**Point 1:** “I would clarify in the abstract that psychiatric disorders were defined two ways, first by ICD codes and second by ICD plus prescriptions - as written it seems it is only the second definition which could be restrictive.”

**Reply:** Thank you for this comment. We have now explicitly distinguished the two exposure definitions in the Abstract as follows (page 2 line 57-61):

- “In these people, we ascertained cases of mental disorders using two approaches: (1) per ICD-10 diagnostic codes for substance use, psychotic, affective, and anxiety disorders and (2) per ICD-10 diagnostic codes for the above mental disorders coupled with a prescription for anxiolytics/hypnotics/sedatives, antidepressants, antipsychotics or stimulants per ATC codes.”.

**Point 2:** “The abstract should briefly describe the findings for all psychiatric disorder groups assessed - as it current does not present affective disorders.”

**Reply:** Thank you for this comment. We have now included the results for affective disorders in the Abstract as follows (page 2 line 79-82):

- “People diagnosed and treated for affective disorders had a decreased risk of both COVID-19 and all-cased death in epoch 3 (from 0.87 [95% CIs, 0.79-0.96] to 0.90 [95% CIs, 0.83-0.99]), but demonstrated null effects in other epochs.”.

We have now also elaborated on these in the Results as follows:

- “Additionally, people diagnosed and treated for affective disorders had a decreased risk of COVID-19 death in epoch 3, both 28 (0.88 [95% CIs, 0.79-0.98]) and 60 (0.88 [95% CIs, 0.79-0.98]) days post-infection, but demonstrated null effects in other epochs.” (page 8 line 356-359).
- “In addition, people diagnosed and treated for affective disorders had a decreased risk of all-cause death in epoch 3, both 28 (0.87 [95% CIs, 0.79-0.96]) and 60 days (0.90 [95% CIs, 0.83-0.99]) following SARS‑CoV‑2 infection, but demonstrated null effects in other epochs.” (page 9 line 383-386).

**Point 3:** “I don't necessarily think vaccination status can be considered a true confounder, which should precede the exposure and be associated with the outcome (e.g., sex/gender); while vaccination status is likely related to psychiatric disorders, in this sample it seems that psychiatric diagnoses were believed to precede vaccination (e.g., arguably it is possible vaccination is on the pathway between psychiatric diagnoses and covid outcomes). Similarly for physical comorbidities - depending on the timing, it is possible these preceded or occurred after psychiatric disorder onset. I don't think it is necessarily invalid to adjust for these factors (particularly comorbidities depending on their timing of diagnosis), but the authors should describe more precisely how they conceptualize these variables in the causal structure of relationships and why they are adjustment for them.”

**Reply:** Thank you for raising this important point. Pre-existing comorbidity has been consistently shown to be among the factors most strongly associated with worse prognosis following a SARS‑CoV‑2 infection [19]. Correspondingly, existing research has consistently demonstrated that under-vaccination is strongly associated with severe COVID-19 outcomes [20], including across different pandemic epochs [21].

Therefore, to provide associations between mental disorders and post-SARS‑CoV‑2 infection mortality that are not confounded by pre-existing comorbidity and vaccination status, we controlled for both pre-existing comorbidity and vaccination status, even though neither of them can be considered the cause of mental disorders in this study. Our interpretation of confounders thus aligns with VanderWeele’s “disjunctive cause criterion”, in which one controls for covariates that are causes of the exposure *or* causes of the outcome *or* are causes of both [12, 22].

We have now explicitly provided in the Methods how we conceptualized confounders as follows (page 6 line 255-257):

- “In our identification and selection of potential confounders, we followed the “disjunctive cause criterion”, in which one controls for covariates that are causes of the exposure *or* causes of the outcome *or* are causes of both [12, 22].”.

However, while it is not plausible that having a pre-existing mental disorder would influence the effect of vaccinations (i.e., a vaccination being a mediator), it cannot be ruled out that in some instances physical comorbidity is the consequence of having pre-existing mental disorders. Consequently, it is possible that for some individuals, physical comorbidity would act as a mediator, thus raising the possibility of overadjustment bias [23].

We have now explicitly elaborated on this possibility in the Discussion as follows (page 11 line 514-518):

- “Fourth, while clinically-recorded physical comorbidities are among the factors most strongly associated with worse prognosis following a SARS‑CoV‑2 infection [19], we cannot rule out that in some individuals with mental disorders, they would act as a mediator instead of an confounder, thus raising the possibility of overadjustment bias [23].”.

**Point 4:** “What was the reason behind including individuals aged 10 and above?”

**Reply:** Thank you for this question. Adolescence, typically defined as an age range from 10 to 19 years, is a life stage in which patterns of health and behaviour dynamically develop [24] and when most mental disorders have their typical onset [25]. In accordance with a comprehensive study assessing incidence rates and lifetime risk of mental disorders, we considered the age of 10 years as the earliest plausible onset age of the studied mental disorders [26].

We have now specific in the Methods as follows (page 5 line 216-218):

- “We retrieved all individuals aged 10 or above – the earliest plausible onset age of the studied mental disorders [26] – with first-ever laboratory-confirmed SARS‑CoV‑2 infection occurring in five epochs:”.

**Point 5:** “Potential limitation of under ascertainment of COVID-19 cases if individuals did not have data in the nationwide testing - were there at home tests available?”

**Reply:** Thank you for this question. If there were no data on SARS‑CoV‑2 testing for a particular individual in the Information System of Infectious Diseases, then we were unable to ascertain their testing status. This would be a limitation particularly if our aim were to estimate SARS‑CoV‑2 testing and/or infection rates in people with and without mental disorders. However, in our study, we conditioned on those who tested positively for a SARS‑CoV‑2 infection. Moreover, if the tested people with mental disorders had potentially different characteristics than the tested people without mental disorders, then we matched these two groups on multiple key characteristics and subsequently controlled for a wide range of additional covariates. Therefore, while potential under-ascertainment of SARS‑CoV‑2 cases cannot be ruled out, it seems improbable that it would materially impact our estimates.

**Point 6:** “What is the distinction between ICD-10 cause and external cause of death?”

**Reply:** Thank you for raising this question. External causes of death refer to unnatural causes of death such as suicides, accidents or homicides. We included this information in our initial submission to provide a full description of the registers; however, this particular information has no implications for the current study given that the outcomes were either all-cause mortality or COVID-19 mortality.

**Point 7:** “The ICD codes used for the psychiatric disorders should be listed in the supplement. Were stress-related disorders included (e.g., was PTSD included in anxiety disorders)?”

**Reply:** Thank you for this question. We considered all ICD-10 diagnoses related to neurotic, stress-related and somatoform disorders and labelled them as “anxiety disorders”. This included also post-traumatic stress disorder (PTSD).

We have now provided a table in the Supplement Methods with codes and names per ICD-10 and the corresponding labels we used in our study.

**Point 8:** “What was the justification behind including the second definition of psychiatric disorders (i.e., plus prescriptions) - do the authors think this identified a subgroup with more severe disease requiring rx treatment? It is also possible that these individuals have better managed psychiatric symptoms if they are receiving efficacious medications, so somewhat hard to interpret.”

**Reply:** Thank you for this comment. We ascertained cases of mental disorders using two approaches: (1) per ICD-10 diagnostic codes for substance use, psychotic, affective, and anxiety disorders and (2) per ICD-10 diagnostic codes for the above mental disorders coupled with prescription for anxiolytics/hypnotics/sedatives, antidepressants, antipsychotics or stimulants per ATC codes.

The second ascertainment approach potentially identified two groups of people with mental disorders: (1) those who might be severe enough cases to require treatment with psychopharmaceuticals and (2) those who might have their condition better managed. While these two groups would have potentially different outcomes, our aim was to investigate whether the two case ascertainment approaches would produce broadly consistent results.

We have now elaborated on the rationale behind using two ascertainment approaches in the Methods as follows (page 6 line 250-252):

- “We used the two case ascertainment approaches to investigate the consistency of estimates across different exposure definitions: broadly consistent results between these would increase the confidence in the robustness of inferences.”.

Consequently, the broadly consistent results between the two ascertainment approaches across mental epoch-mental disorder combinations suggest that the results we detected in this study are unlikely to be driven by exposure definitions.

We have now elaborated on this in the Discussion as follows (page 9 line 405-411):

- “Using Czech national health register data, we demonstrated that people with pre-existing psychotic disorders were more likely to die of COVID-19 or due to any cause following SARS‑CoV‑2 infection during each pandemic phase. We demonstrated less robust associations for COVID-19 deaths in people with substance use disorders but they had a consistent and sustained elevated risk of all-cause mortality following SARS‑CoV‑2 infection. The two exposure definitions produced broadly consistent results across each epoch-mental disorder combination.”.

**Point 9:** “How was vaccination status defined - any vaccine at least 14 days after, or full vaccine regimen? Any distinction by vaccination type (depending on what was available) or booster doses?”

**Reply:** Thank you for this comment. We distinguished those who (1) were not vaccinated, (1) received the first dose of two-dose regimen, (3) received the second dose of two-dose regimen or one-dose regimen (i.e., fully vaccinated), (4) and those who received a booster. To consider a dose, it had to be administered more than 14 days before the infection. For instance, if an individual received the first dose of two-dose regimen 2 months before the infection but the second dose 10 days before the infection, then we considered them as receiving the first dose of two-dose regimen. We used this strategy since vaccination do not confer an immediate protection and matching partially and fully inoculated individuals could bias the survival estimates.

We have now specified this in the Methods as follows (page 6 line 261-267):

- “In the three subsequent epochs, we exactly-matched on age, sex, month and year of infection, vaccination status, and the CCI. Since vaccination do not confer an immediate protection, we did not consider vaccinations that were administered 14 or less days before the infection. For instance, when an individual received the first dose of a two-dose regimen more than 14 days before the infection, whereas the second dose 14 or less days before the infection, we considered them as having received the first dose at the time of the infection.”.

**Point 10:** “How might having unmatched psychiatric patients impact the findings? Or how do the authors interpret the lack of matched non-psychiatric patients for these individuals?”

**Reply:** Thank you for this comment. In each cohort, the proportion of unmatched individuals was less than 10%. In cohorts of people with psychotic and substance use disorders, the highest proportion of unmatched individuals was approximately 6%. While in principle any proportion of unmatched individuals may induce “bias due to incomplete matching” [27], we think that these proportions are too low to be responsible for the detected results.

However, the differences between matched and unmatched individuals – as provided in Supplementary Table 2-3 – suggest that the unmatched individuals tended to be, on average, older and have a higher number of comorbidities. Thus, it is possible that the unmatched individuals tended to be the most severe cases of mental disorders. Since the most severe cases of mental disorders are likely to have the worst health outcomes, it is possible that the omission of these individuals led to bias, with the most likely direction of bias being the underestimation of the true effects.

We have now explicitly elaborated on this in the Discussion as follows (page 11 line 509-514):

- “Third, we were able to match the vast majority of people with mental disorders with counterparts without mental disorders; however, “bias due to incomplete matching” cannot be ruled out [27]. In particular, the unmatched individuals with mental disorders tended to be, on average, older and have a higher number of comorbidities. Since these individuals can be expected to have the worse outcomes post-infection, the most plausible direction of bias seems to be the underestimation of true effects.”.

**Point 11:** “More detail should be added in the description of the negative control analyses in the main paper description - it is currently a bit unclear what the actual analyses are. E.g., these seem to be negative control exposures, but it is unclear how there are specific proportions for the studied disorders (i.e., the psychiatric disorders)?”

**Reply:** Thank you for raising this point. The rationale behind negative control analyses was to select exposures that are known or assumed to be unrelated to the outcome within the chosen cohorts [28]. Then, null associations between such exposures and the outcome would suggest the absence of collider bias [28].

We considered (1) migraine (ICD-10 code G43), (2) fracture of forearm (ICD-10 code S52), (3) acne (ICD-10 code L70), (4) mild allergies (ICD-10 codes J301, L500 and L23), and (5) transport accidents (ICD-10 codes V01-V99) occurring in the time period of five years prior to the positive SARS-CoV-2 test as exposures in the negative control analyses.

The results reported in Supplementary Table 14 correspond to the performed negative control analyses. The column “Tests, n” denoted the total number of performed, with the theoretical maximum being 40 tests per one epoch-mental disorder combination (2 case ascertainment definitions X 5 exposures X 4 outcomes). The columns “Non-null tests, n” and “Non-null tests, %” indicated the number and proportion of non-null tests, respectively. Then, since some of our cohorts are considerably large (i.e., anxiety disorders in epochs 2 to 5), it would be possible to have non-null results even if the effect sizes were negligible [29]. Thus, in the column “Mean effect size”, we provided a complementary averaged hazards ratio across the 40 tests per one epoch-mental disorder combination. Here, results closer to 1 would suggest the absence of collider bias.

We have now elaborated on these in the Methods as follows (page 7 line 309-320):

- “Having a history of a mental disorder might influence the risk of being tested for SARS-CoV-2 infection; thus, restricting the analysis to individuals who had a positive test might lead to collider bias [28]. To examine potential presence of collider bias, we conducted negative control analyses by assessing the associations between the characteristics that are expected to be unrelated to the outcome (e.g., accidents) and the outcome itself within the chosen cohorts [28]. We reported the total number and proportion of non-null tests, with the theoretical maximum being 40 (or 100%) per one epoch-mental disorder combination. Since some of our cohorts were considerably large (i.e., anxiety disorders in epochs 2 to 5), it would be possible to have non-null results even if the effect sizes were negligible [29]. Thus, we complementarily provided averaged HRs across the 40 tests per one epoch-mental disorder combination. Proportion of non-null tests closer to 0% and/or averaged HRs closer to 1 would suggest the absence of collider bias (see details in Supplementary Methods).”.

and in the Results as follows (page 9 line 391-398):

- “In negative control analyses, the proportion of non-null tests ranged from 7.50% (averaged HRs = 0.95) to 11.76% (averaged HRs = 1.07) for substance use disorders, from 0.00% (averaged HRs = 0.59) to 15.00 (averaged HRs = 0.88) for people with psychotic disorders, from 0.00% (averaged HRs = 2.28) to 17.50% (averaged HRs = 0.82 and 0.88) for people with affective disorders, and from 0.00% (averaged HRs = 0.84 and 1.21) to 40.00% (averaged HRs = 0.70) for people with anxiety disorders. Proportion of non-null tests closer to 0% and/or averaged HRs closer to 1 increase the confidence in the lack of collider bias.”.

Moreover, we have now included additional information on the approach in Supplementary Methods as follows:

- “To examine potential presence of collider bias, we conducted negative control analyses by assessing the associations between the characteristics that are expected to be unrelated to the outcome and the outcome within the chosen cohorts [28]. To do so, we considered the occurrence of (1) migraine (ICD-10 code G43), (2) fracture of forearm (ICD-10 code S52), (3) acne (ICD-10 code L70), (4) mild allergies (ICD-10 codes J301, L500 and L23), and (5) transport accidents (ICD-10 codes V01-V99) in the time period of five years prior to the positive SARS-CoV-2 test. We assessed the occurrence of each of these separately. Then, we fitted stratified Cox proportional hazards models with the above characteristics being the exposures, while using the confounders and outcomes from the main analysis. We reported the total number and proportion of non-null tests, with the theoretical maximum being 40 (or 100%) per one epoch-mental disorder combination. Since some of our cohorts were considerably large (i.e., anxiety disorders in epochs 2 to 5), it would be possible to have non-null results even if the effect sizes were negligible [29]. Thus, we complementarily provided averaged hazards ratio across the 40 tests per one epoch-mental disorder combination. Proportion of non-null tests closer to 0% and/or averaged hazards ratios closer to 1 would suggest the absence of collider bias.”.

**Point 12:** “When discussing the previous findings of antipsychotics and decreased mortality in individuals with psychosis, it would be helpful to explicitly describe the current findings for psychosis plus medications, and potentially sensitivity analyses directly testing this same hypothesis (psychosis plus the same antipsychotic tested in the prior study).”

**Reply:** Thank you for this comment. The associations between antipsychotics use and risk of all-cause mortality in people with schizophrenia demonstrated in the Discussion did not refer to the pandemic period. These are pre-pandemic results showing that antipsychotics use, in general, decreases the risk of all-cause mortality in people with schizophrenia.

We have now elaborated on this in the Discussion as follows (page 10 line 445-450):

- “Pharmacological treatments for psychotic disorders are known to contribute to metabolic disturbances [30], interact with or limit the use of treatment for somatic conditions, thus potentially contributing to lower survival also post-SARS‑CoV‑2 infection. However, pre-pandemic research has demonstrated that the use of antipsychotics is associated with decreased risk of all-cause mortality in people with schizophrenia [31], with no differences between concomitant use of several ones compared with monotherapy [32].”

In our study, we used the case ascertainment involving diagnosis and prescription of psychopharmaceuticals, including antipsychotics, to investigate whether we would obtain broadly consistent results across different exposure definitions. Consequently, the broadly consistent results between the two ascertainment approaches across epoch-mental disorder combinations suggest that the results we detected in this study are unlikely to be driven by exposure definitions.

We have now elaborated on this in the Discussion as follows (page 9 line 405-411):

- “Using Czech national health register data we demonstrated that people with pre-existing psychotic disorders were more likely to die of COVID-19 or due to any cause following SARS‑CoV‑2 infection during each pandemic phase. We demonstrated less robust associations for COVID-19 deaths in people with substance use disorders but they had a consistent and sustained elevated risk of all-cause mortality following SARS‑CoV‑2 infection. The two exposure definitions produced broadly consistent results across each epoch-mental disorder combination.”.

**Point 13:** “The results section should describe and discussion should interpret associations with "any mental disorder", since these were assessed.”

**Reply:** Thank you for this comment. We have now provided the results for any of the studied mental disorder in the Results as follows:

- “The results for any studied mental disorder were – regardless of case ascertainment definition – consistent with a null effect in all epochs.” (page 8 line 359-360).
- “The results for any studied mental disorder were – regardless of case ascertainment definition – consistent with a null effect in all epochs.” (page 9 line 386-387).

We have refrained from discussing these results because of the inherent heterogeneity of such a broad category coupled with vastly different definitions across existing studies that would make comparisons unfeasible.

**Reviewer #2**

**Point 1:** “In the Methods section, it is stated that exact matching was performed on age, sex, month+year of infection & Charlson Comorbidity Index (CCI), and also vaccination status in the latter three epoches. Further confounding was addressed by adjusting for region of permanent residency, overall number of contacts with inpatient/outpatient services, and medications up to one year prior.

A major concern would be that known COVID-19 mortality risk factors (e.g. race/ethnicity, comorbidities not covered in the CCI such as hypertension, smoker status, etc. from "Mortality-related risk factors of COVID-19: a systematic review and meta-analysis of 42 studies and 423,117 patients", Dessie & Zewotir, BMC Infectious Diseases 21, 855 [2021]) appear not to be currently included in either the exact matching procedure, or the later adjustments. While this potential residual confounding is acknowledged as a limitation, the impact is possibly significant due to the number of such risk factors that were not considered. Some justification/estimation of the magnitude of the residual confounding would thus be welcome.”

**Reply:** Thank you for raising this important point. While the comorbidity index we used in this study did not include hypertension, we subsequently adjusted for the prescription of antihypertensives (ATC code C02) in the period of one year before the infection. Then, we had no information on body mass index, smoking status, and socioeconomic status *per se*; however, all of these are likely to influence an individual’s overall health [2-7]. To consider overall health, we matched on a comorbidity index, and subsequently adjusted for the overall number of contacts with inpatient services and the overall number of contacts with outpatient services (disregarding contacts related to the exposure), and prescription medications, including antihypertensives and statins (ATC code C10AA) that would be administered for conditions commonly present in people who smoke or who are obese [8-11].

As demonstrated by VanderWeele using the causal diagram below, adjusting for a measured covariate C, even in the presence of an unmeasured covariate U, removes confounding between exposure A and outcome Y [12].

Correspondingly, while we did not directly control for hypertension, body mass index, smoking status, and socioeconomic status, adjusting for antihypertensives and overall health, represented by a comorbidity index, overall number of contacts with inpatient services and overall number of contacts with outpatient services, and prescription medications, likely contributed to substantial reduction of potential confounding due to these covariates.

We have now elaborated on this in the Discussion as follows (page 11 line 497-509):

- “Second, we exactly-matched on key sociodemographic and clinical covariates and subsequently adjusted for a wide range of additional health-related confounders; however, we did not control for a number of previously identified influential clinical, sociodemographic, and behavioural confounders [1]. In particular, we had no information on body mass index, smoking status, and socioeconomic status *per se*; however, these are likely to influence an individual’s overall health [2-7], which we considered by controlling for a comorbidity index, overall number of contacts with inpatient and outpatient services, and prescription medications, including antihypertensives and statins that would be administered for conditions commonly present in people who smoke or who are obese [8-11]. These steps likely reduced the level of confounding due to these covariates; however, we cannot rule out that part of the detected associations is still due to residual confounding, both related to these known and directly unmeasured and potential unknown confounders, with the most plausible direction of bias being the overestimation of true effects.”.

**Point 2:** “In the Matching subsection, it is stated that each exposed individual was matched with up to five unique unexposed counterparts. This suggests that such individuals would be matched with fewer than five counterparts (possibly zero, equivalent to exclusion), if fewer exact matches according to the relevant criteria were found. This would appear to possibly bias the analysis towards more common demographics for controls, since less-common demographic combinations would have fewer matches included. It might be clarified as to the distribution of matches (zero to five, with zero stated as <10% for each cohort), and possibly explore methods to mitigate the matching bias (e.g. matching with replacement).”

**Reply:** Thank you for this comment. We followed the reviewer’s suggestion, and now we present the distribution of matches in Supplementary Table 4-5. Across each epoch-mental disorder combination, the vast majority of people with pre-existing mental disorders had four to five matched unique counterparts without the respective mental disorder. This would suggest that the results would not be biased towards more common combinations of matching variables.

Moreover, throughout the study, we used stratified Cox proportional hazards models, with each stratum consisting of one individual with a given mental disorder and up to five individually-matched counterparts without that mental disorder. The procedure involves comparisons within each strata, allowing to control for the characteristics used in matching [33]. Consequently, while it is possible that some people with mental disorders did not have exactly five matched counterparts, the number of counterparts without the given mental disorder in a given stratum should not bias the estimates towards more common combinations of matching variables.

On the other hand, while matching with replacement increases the absolute cohort size, it decreases the effect cohort size and thus decreases precision in estimates [34]. Moreover, matching with replacement may increase reliance on a relatively few unexposed individuals [35] and contribute to inference becoming more complex since the unexposed individuals are no longer independent [36].

**Point 3:** “In the Additional Confounders subsection, "prescription medications" are mentioned. It might be clarified whether this includes medications for SARS‑CoV-2 infection not including vaccination, and also other treatments (e.g. mechanical ventilation).”

**Reply:** Thank you for this comment. We considered medications that were prescribed in the period of one year before the infection. We used commonly prescribed medications to potentially capture an individual’s overall health that might influence their survival post-infection. For these reasons, we did not consider medications or other treatment modalities following the infection itself.

**Point 4:** “In the Sensitivity Analyses subsection, it is stated that E-values are provided in Supplementary Table 11, and that higher values increase the confidence that the detected associations are not due to unaccounted for confounding. Some context on the magnitudes of the E-values might be provided, if possible - can they be interpreted as confidence percentages?”

**Reply:** Thank you for this comment. The E-value indicate the strength of association – here expressed in hazards ratio – an unmeasured confounder, or set of confounders, would need to have with both the exposure and the outcome to nullify the association between the exposure and the outcome observed in the model [37] . For example, an E-value of 3 would suggest that to nullify the association between the exposure and the outcome observed in the model, an unmeasured confounder, or set of confounders, would need to be associated with both the exposure and the outcome by hazards ratio of 3 [37]. Smaller E-values indicate lower confidence in the results not being due to residual confounding [37].

We have now elaborated on this in the Methods as follows (page 7-8 line 322-328):

- “To assess the level of potential unmeasured confounding, we calculated E-values for each of our regression model where the results were not consistent with a null effect. The E-value indicate the strength of association – here expressed in hazards ratio – an unmeasured confounder, or set of confounders, would need to have with both the exposure and the outcome to nullify the association between the exposure and the outcome observed in the model [37]. Smaller E-values indicate lower confidence in the results not being due to residual confounding [37].”.

We have now also expanded the reporting of E-values in the Results as follows (page 9 line 400-403):

- “For models not consistent with a null-effect, the E-values ranged from 1.71 to 3.22 for substance use disorders, from 2.21 to 3.39 for psychotic disorders, and from 1.50 to 1.88 for anxiety disorders. Higher E-values increase the confidence that the detected associations are not due to unaccounted for confounding. See details in Supplementary Table 15-16.”.

**Reviewer #3**

**Point 1a:** “This is a well written and properly executed paper of Czech health registry data analysis comparing individuals infected with SARS-COV-2 having a mental health disorder (i.e., psychotic disorders, SUDs and anxiety/affective disorders) in the 5 years prior to the pandemic. Authors claim this is the first whole-country registry data analysis including a diverse range of patient-settings and controlling for vaccination uptake and somatic co-morbidity. This indeed makes this a very valuable study with a high degree of generalizability of findings. But at the same time, the introduction is a bit limited in its reflection on the existing literature regarding the risk of COVID-19-related mortality among the population with pre-existing mental disorders (e.g., recent study of Gibbs et al., on Swedish registry data or umbrella review of Bertolini et al., 2023 not covered).”

**Reply:** Thank you for this comment. We have now reworded the Introduction to include a broader presentation of existing research, including the Swedish register-based study that was suggested by the review as follows (page 4 line 141-146):

- “Previous research has demonstrated that individuals with a diagnosis of a mental disorder had an increased risk for SARS-CoV-2 infection [38] as well as for breakthrough infection after vaccination [39]. Existing evidence on mortality post-infection showed consistently increased risk in people with psychotic [40-47] and substance use disorders [48-51], elevated risk [41, 43, 46, 52] or null effects [45, 48] in people with anxiety disorders, and elevated risk [41, 44, 46, 53] or null effects [45] in people with affective disorders.”.

We have not included the suggested umbrella review [54] because we wished to focus on primary studies using national register-based data.

**Point 1b:** “Role of other potential relevant factors beyond low SES, BMI and smoking such as ageing not properly discussed. This could be improved.”

**Reply:** Thank you for this important remark. From the most important previously identified clinical, sociodemographic, and behavioural confounders, we were unable to directly control for body mass index, smoking status, and socioeconomic status [1]. However, all these covariates are known to influence an individual’s overall health [2-7]. To consider overall health, we matched on a widely used comorbidity index and subsequently adjusted for the overall number of contacts with inpatient services and the overall number of contacts with outpatient services (disregarding contacts related to the exposure), and prescription medications, including antihypertensives (ATC code C02) and statins (ATC code C10AA) that would be administered for conditions commonly present in people who smoke or who are obese [8-11].

As demonstrated by VanderWeele using the causal diagram below, adjusting for a measured covariate C, even in the presence of an unmeasured covariate U, removes confounding between exposure A and outcome Y [12].

Correspondingly, while we did not directly control for body mass index, smoking status, and socioeconomic status, adjusting for overall health, represented by a comorbidity index, overall number of contacts with inpatient services and overall number of contacts with outpatient services, and prescription medications, likely contributed to substantial reduction of potential confounding due to these covariates. On the other hand, we cannot rule out that there are also unknown confounders that would influence our estimates. Consequently, the most plausible direction of bias seems to be the overestimation of true effects.

We have now elaborated on these in the Discussion as follows (page 11 line 497-509):

- “Second, we exactly-matched on key sociodemographic and clinical covariates and subsequently adjusted for a wide range of additional health-related confounders; however, we did not control for a number of previously identified influential clinical, sociodemographic, and behavioural confounders [1]. In particular, we had no information on body mass index, smoking status, and socioeconomic status *per se*; however, these are likely to influence an individual’s overall health [2-7], which we considered by controlling for a comorbidity index, overall number of contacts with inpatient and outpatient services, and prescription medications, including antihypertensives and statins that would be administered for conditions commonly present in people who smoke or who are obese [8-11]. These steps likely reduced the level of confounding due to these covariates; however, we cannot rule out that part of the detected associations is still due to residual confounding, both related to these known and directly unmeasured and potential unknown confounders, with the most plausible direction of bias being the overestimation of true effects.”.

**Point 2:** “Methodology description is sufficiently clear and authors provided detailed information on relevant additional analyses (e.g., unmeasured confounding) and also the potential collider bias has been accurately addressed by performing a sensitivity analysis by controlling for negative tests as well (instead only on those with a positive test). Would be good though that authors provide a bit more explanation on collider bias in this respect by explaining these negative control analyses as a commonly used tool of epidemiologists in assessing the validity of their findings and identifying potential sources of bias or errors in the research. That in this analysis, exposure status to COVID-19 infection and other characteristics are evaluated and expected to be unrelated to the outcome of interest. This concept may be challenging for readers to understand, so please offer a more straightforward explanation.”

**Reply:** Thank you for this comment. The rationale behind negative control analyses was to select exposures that are known or assumed to be unrelated to the outcome within the chosen cohorts [28]. Then, null associations between such exposures and the outcome would suggest the absence of collider bias [28].

We considered (1) migraine (ICD-10 code G43), (2) fracture of forearm (ICD-10 code S52), (3) acne (ICD-10 code L70), (4) mild allergies (ICD-10 codes J301, L500 and L23), and (5) transport accidents (ICD-10 codes V01-V99) occurring in the time period of five years prior to the positive SARS-CoV-2 test as exposures in the negative control analyses.

The results reported in Supplementary Table 14 correspond to the performed negative control analyses. The column “Tests, n” denoted the total number of performed, with the theoretical maximum being 40 tests per one epoch-mental disorder combination (2 cohort definitions X 5 exposures X 4 outcomes). The columns “Non-null tests, n” and “Non-null tests, %” indicated the number and proportion of non-null tests, respectively. Then, since some of our cohorts are considerably large (i.e., anxiety disorders in epochs 2 to 5), it would be possible to have non-null results even if the effect sizes were negligible [29]. Thus, in the column “Mean effect size”, we provided a complementary averaged hazards ratio across the 40 tests per one epoch-mental disorder combination. Here, results closer to 1 would suggest the absence of collider bias.

We have now elaborated on these in the Methods as follows (page 7 line 309-320):

- “Having a history of a mental disorder might influence the risk of being tested for SARS-CoV-2 infection; thus, restricting the analysis to individuals who had a positive test might lead to collider bias [28]. To examine potential presence of collider bias, we conducted negative control analyses by assessing the associations between the characteristics that are expected to be unrelated to the outcome (e.g., accidents) and the outcome itself within the chosen cohorts [28]. We reported the total number and proportion of non-null tests, with the theoretical maximum being 40 (or 100%) per one epoch-mental disorder combination. Since some of our cohorts were considerably large (i.e., anxiety disorders in epochs 2 to 5), it would be possible to have non-null results even if the effect sizes were negligible [29]. Thus, we complementarily provided averaged HRs across the 40 tests per one epoch-mental disorder combination. Proportion of non-null tests closer to 0% and/or averaged HRs closer to 1 would suggest the absence of collider bias (see details in Supplementary Methods).”.

and in the Results as follows (page 9 line 391-398):

- “In negative control analyses, the proportion of non-null tests ranged from 7.50% (averaged HR = 0.95) to 11.76% (averaged HR = 1.07) for substance use disorders, from 0.00% (averaged HR = 0.59) to 15.00 (averaged HR = 0.88) for people with psychotic disorders, from 0.00% (averaged HR = 2.28) to 17.50% (averaged HR = 0.82 and 0.88) for people with affective disorders, and from 0.00% (averaged HR = 0.84 and 1.21) to 40.00% (averaged HR = 0.70) for people with anxiety disorders. Proportion of non-null tests closer to 0% and/or averaged HRs closer to 1 increase the confidence in the lack of collider bias.”.

Moreover, we have now included additional information on the approach in Supplementary Methods as follows:

- “To examine potential presence of collider bias, we conducted negative control analyses by assessing the associations between the characteristics that are expected to be unrelated to the outcome and the outcome within the chosen cohorts [28]. To do so, we considered the occurrence of (1) migraine (ICD-10 code G43), (2) fracture of forearm (ICD-10 code S52), (3) acne (ICD-10 code L70), (4) mild allergies (ICD-10 codes J301, L500 and L23), and (5) transport accidents (ICD-10 codes V01-V99) in the time period of five years prior to the positive SARS-CoV-2 test. We assessed the occurrence of each of these separately. Then, we fitted stratified Cox proportional hazards models with the above characteristics being the exposures, while using the confounders and outcomes from the main analysis. We reported the total number and proportion of non-null tests, with the theoretical maximum being 40 (or 100%) per one epoch-mental disorder combination. Since some of our cohorts were considerably large (i.e., anxiety disorders in epochs 2 to 5), it would be possible to have non-null results even if the effect sizes were negligible [29]. Thus, we complementarily provided averaged hazards ratio across the 40 tests per one epoch-mental disorder combination. Proportion of non-null tests closer to 0% and/or averaged hazards ratios closer to 1 would suggest the absence of collider bias.”.

**Point 3a:** “The distinction between diagnosed and diagnosed and treated is clear from the Tables and mentioned in the text but rationale behind it is a bit lacking in the methodology.”

**Reply:** Thank you for this remark. We ascertained cases of mental disorders using two approaches: (1) per ICD-10 diagnostic codes for substance use, psychotic, affective, and anxiety disorders and (2) per ICD-10 diagnostic codes for the above mental disorders coupled with prescription for anxiolytics/hypnotics/sedatives, antidepressants, antipsychotics or stimulants per ATC codes.

The second ascertainment approach potentially identified two groups of people with mental disorders: (1) those who might be severe enough cases to require treatment with psychopharmaceuticals and (2) those who might have their condition better managed. While these two groups would have potentially different outcomes, our aim was to investigate whether the two ascertainment approaches would produce broadly consistent results.

We have now elaborated on the rationale behind using two ascertainment approaches in the Methods as follows (page 6 line 250-252):

“We used the two ascertainment approaches to investigate the consistency of estimates across different exposure definitions: broadly consistent results between these would increase the confidence in the robustness of inferences.”.

Consequently, the broadly consistent results between the two ascertainment approaches across mental epoch-mental disorder combinations suggest that the results we detected in this study are unlikely to be driven by exposure definitions.

We have now elaborated on this in the Discussion as follows (page 9 line 405-411):

- “Using Czech national health register data we demonstrated that people with pre-existing psychotic disorders were more likely to die of COVID-19 or due to any cause following SARS‑CoV‑2 infection during each pandemic phase. We demonstrated less robust associations for COVID-19 deaths in people with substance use disorders but they had a consistent and sustained elevated risk of all-cause mortality following SARS‑CoV‑2 infection. The two exposure definitions produced broadly consistent results across each epoch-mental disorder combination.”.

**Point 3b:** “Same holds for the different epochs; it seems that findings from epoch 3 are a bit different (e.g., less broad CI's and more often significantly different from 1 (no difference)) compared to other epochs. Is there a role of the waves - such as in terms of vaccination status? Here, the reader could be better guided in interpreting the tables and (relevant) figures.”

**Reply:** Thank you for this remark. Epoch 4 followed after the second wave of the pandemic which hit Czechia particularly hard (epoch 2 and 3). In addition to this, epoch 4 covered the period from 1^st^ April 2021 to 31^st^ October 2021 that corresponds to spring and summer in Czechia. For these reasons, the number of infections and therefore the cohort sizes were smaller than in epoch 3 or the subsequent epoch 5, resulting also in wider confidence intervals for estimates from this epoch.

We have now provided the number of incident infections corresponding to phases of the pandemic covered by this study in the Methods as follows (page 5 line 180-190):

- “The first wave of the pandemic in Czechia lasted roughly from 1^st^ March 2020 to 30^th^ September 2020, with a State of Emergency being in place from 12^th^ March 2020 to 25^th^ May 2020. The first wave resulted in 70,968 incident infections [55]. The second wave of the pandemic lasted approximately from 1^st^ October 2020 to 31^th^ March 2020, with a State of Emergency imposed from 5^th^ October 2020 to 11^th^ April 2021. The second wave led to 1,486,198 incident infections [55]. The Czech National Vaccination Strategy was launched in December 2020, and it considered no pre-existing mental disorders as reason for priority inoculation [56]. The period of post-second wave lasted roughly from 1^st^ April 2021 to 31^st^ October 2021, resulting in 231,286 incident infections [55]. Then, the 2021-2022 winter wave lasted from approximately 1^st^ November 2021 to 28^th^ February 2022, and led to 2,017,028 incident infections [55].”.

**Point 4:** “In the Discussion, the authors delve into the most relevant underlying explanatory mechanisms of the increased mortality risk in individuals with psychotic disorders or substance abuse, such as individual and structural lifestyle factors like low socioeconomic status (SES), high body mass index (BMI), smoking, (interactions with) inconsistent medication use, as well as late recognition of COVID-related physical symptoms and limited access to somatic healthcare, etc. Authors righteously address the shortcoming that they do not control for several of these factors in the current study. I also wondered about the quality of the health registry data. Main shortcomings in the data from national health registries of European countries like Czech republic are not addressed in the Discussion such as potential of underregistration, uncertainties in coding of diagnoses, selection bias, and lack of longitudinal data of individual patients beyond 5 years ago.”

**Reply:** Thank you for this remark. The Czech healthcare system is based on the compulsory insurance model, with the population coverage being virtually universal [57]. Then, our data were national and covered all healthcare settings, including primary care, and all serologically-confirmed SARS‑CoV‑2 infections. Given universal health coverage and the fact that virtually the entire Czech healthcare system was covered, the presence of selection bias that would have a material impact on our estimates, seems unlikely. Then, there are data covering the entire healthcare system since 2010: we conceptually restricted the histories of mental disorders and somatic comorbidities to the past five years. We did so since health conditions recorded further away in an individual’s past might be less relevant to their clinical profile at the of time infection.

Potential under-registration and/or errors in diagnoses coding cannot be ruled out. However, beyond monitoring of public health, information from the registers is used to reimburse the service providers by insurance companies. The insurance companies have their own internal mechanisms to evaluate the validity of reported information, as well as strong economic incentives to ensure that the reporting is indeed correct. For these reasons, we think that it is reasonable to assume that the extent of reporting and validity of the diagnoses is adequate.

We have not elaborated on this in the Discussion as follows (page 11-12 line 518-521):

- “Fifth, both the curator of the data, IHIS, and the insurance companies who use them to reimburse service providers employ mechanisms to ensure the validity of data; however, all diagnoses used in this study have not been fully validated yet. Thus, under-registration and/or errors in diagnoses coding cannot be ruled out.”.

**Point 5:** “It is also noteworthy and a strength in this study that multiple waves in the pandemic show similar results but authors do not reflect on the Czech (or EU) situation during the pandemic waves.”

**Reply:** Thank you for this comment. We have now provided a brief discussion of the Czech pandemic situation during the study period in the Discussion as follows (page 5 line 180-190):

- “The first wave of the pandemic in Czechia lasted roughly from 1^st^ March 2020 to 30^th^ September 2020, with a State of Emergency being in place from 12^th^ March 2020 to 25^th^ May 2020. The first wave resulted in 70,968 incident infections [55]. The second wave of the pandemic lasted approximately from 1^st^ October 2020 to 31^th^ March 2020, with a State of Emergency imposed from 5^th^ October 2020 to 11^th^ April 2021. The second wave led to 1,486,198 incident infections [55]. The Czech National Vaccination Strategy was launched in December 2020, and it considered no pre-existing mental disorders as reason for priority inoculation [56]. The period of post-second wave lasted roughly from 1^st^ April 2021 to 31^st^ October 2021, resulting in 231,286 incident infections [55]. Then, the 2021-2022 winter wave lasted from approximately 1^st^ November 2021 to 28^th^ February 2022, and led to 2,017,028 incident infections [55].”.

**Point 6a:** “The main explanation of the findings is being related to previous evidence on fatal health inequalities in these patientgroups - the solutions offered for the future are a bit narrow-minded (in the sense that it is only aimed at scaling up vaccination campaigns in future pandemic). The claim in the Discussion that the results are not fully explicable by differences in vaccination uptake or clinically-recorded physical comorbidities are not clearly substantiated in the Results yet. This could be enhanced. Particularly because the main recommendation made is related to increasing vaccination campaign.”

**Reply:** Thank you for raising this point. We have now specified in the Results that the models controlled for vaccination status and physical comorbidities as follows:

- “In the models adjusting for all considered confounders, including vaccination uptake and clinically-recorded physical comorbidities, people diagnosed with or diagnosed and treated for psychotic disorders had a consistently elevated risk of COVID-19 death in each of the studied epochs, both 28 (adjusted HRs ranging from 1.46 [95% CIs, 1.18-1.79] to 1.93 [95% CIs, 1.12-3.32]) and 60 days (1.46 [95% CIs, 1.20-1.79] to 1.93 [95% CIs, 1.14-3.27]) following SARS‑CoV‑2 infection.“ (page 8 line 341-346).
- “In the models adjusting for all considered confounders, including vaccination uptake and clinically-recorded physical comorbidities, people diagnosed with or diagnosed and treated for psychotic disorders were more likely to die in each of the studied epochs, both 28 (1.43 [95% CIs, 1.23-1.66] to 1.99 [95% CIs, 1.25-3.16]) and 60 days (1.52 [95% CIs, 1.33-1.74] to 1.80 [95% CIs, 1.54-2.10]) post-infection.” (page 8 line 364-368).

Then, we have now broadened the Discussion to include the extent of change required to address the fatal health inequalities as follows (page 10 line 454-459):

- “Overall, the existing evidence and our results suggest the presence of fatal but largely tractable health inequalities in these patient groups. Wholesale system approaches, predicated on evidence-based policy changes and, ideally, combined with evaluation, are required to address the multi-layered factors behind these fatal health inequalities. In a future pandemic or other health emergency, substance use and psychotic disorders need to be considered as a specific vulnerability factor beyond liability to the health threat, itself.”.

**Point 6b:** “At the same time the findings may suggest other factors at play as well - SES, smoking and BMI (which were uncontrolled for).”

**Reply:** Thank you for raising this point. We have now explicitly acknowledged that of part the detected associations might be due to residual confounding in the Abstract as follows (page 2 line 87-89):

- “While it cannot be ruled out that part of the detected associations is due to residual confounding, this excess mortality cannot be fully explained by lower vaccination uptake and more clinically-recorded physical comorbidities in these patient groups.”.

and in the Discussion as follows (page 12 line 531-535):

- “While we cannot rule out that part of the detected associations is due to residual confounding, the consistently increased vulnerability beyond vaccination uptake and clinically-recorded physical comorbidity aligns with existing evidence on fatal health inequalities in these patient groups and underlines the importance of implementing systemic efforts to fully reverse these.”.

**Additional Changes**

**Change 1:** We have now specified which authors received funding as follows (page 13 line 566-570):

- “This study was supported by the Czech Health Research Council (grant number NU22-D-146; TF, HM, KM, PW, and JJ), the Ministry of Health, Czech Republic (funding number 00023752; TF, LP, KM, PM, and PW), and the National Institute for Health and Care Research (NIHR) Applied Research Collaboration East of England at Cambridge and Peterborough NHS Foundation Trust (TF).”.

**Change 2:** We have changed our “Data Sharing” section to “Data Availability Statement”. Furthermore, we have now included how access to data can be requested as follows (page 12 line 539-545):

- “Due to legal regulations, individual-level patient data cannot be publicly deposited. For sharing the data, access must be granted by IHIS. Access to data can be requested at the web page of IHIS: <https://www.uzis.cz/>. The full analytical code is available at a dedicated GitHub repository: https://github.com/libpot/COVID_Mortality_Preexisting_Mental_Disorders.”.

**Change 3:** We have followed the editorial suggestion to include “Czechia” in the title of the article. The new article title is “Mortality from COVID-19 and from All-causes following First-ever SARS‑CoV‑2 Infection in Individuals with Pre-existing Mental Disorders: A National Cohort Study from Czechia”.

**Change 4:** Following the journal’s reporting style, we have now included p-values in Supplementary Results. We have specified this as follows (page 7 line 302-304):

- “In line with the statement from the American Statistical Association on *p*-values [58], we present effect sizes with 95% CIs throughout the manuscript. However, we provide *p*-values as complementary information in Supplementary Results.”.

**Change 5:** Following the journal’s reporting style, we have now changed the reporting of results to “effect size [95% confidence intervals, lower interval-upper interval]” throughout the manuscript.

**Change 6:** LP has started a new position and conducted additional work on this study as part of that position. Therefore, we updated the list of affiliations to reflect this.

**Change 7:** To enhance readability of the main graphs, we removed the line and corresponding axis tick mark denoting hazard ratios = 3.

**References**

1.	Dessie ZG, Zewotir T. Mortality-related risk factors of COVID-19: a systematic review and meta-analysis of 42 studies and 423,117 patients. BMC Infectious Diseases. 2021;21(1):855. doi: 10.1186/s12879-021-06536-3.

2.	Nørtoft E, Chubb B, Borglykke A. Obesity and healthcare resource utilization: comparative results from the UK and the USA. Obesity Science & Practice. 2018;4(1):41-5. doi: <https://doi.org/10.1002/osp4.148>.

3.	Kivimäki M, Batty GD, Pentti J, Shipley MJ, Sipilä PN, Nyberg ST, et al. Association between socioeconomic status and the development of mental and physical health conditions in adulthood: a multi-cohort study. The Lancet Public Health. 2020;5(3):e140-e9. doi: 10.1016/S2468-2667(19)30248-8.

4.	Azagba S, Sharaf MF, Xiao Liu C. Disparities in health care utilization by smoking status in Canada. International Journal of Public Health. 2013;58(6):913-25. doi: 10.1007/s00038-013-0452-7.

5.	Haapanen-Niemi N, Miilunpalo S, Vuori I, Pasanen M, Oja P. The impact of smoking, alcohol consumption, and physical activity on use of hospital services. American Journal of Public Health. 1999;89(5):691-8. doi: 10.2105/AJPH.89.5.691.

6.	Elrashidi MY, Jacobson DJ, St. Sauver J, Fan C, Lynch BA, Rutten LJF, et al. Body Mass Index Trajectories and Healthcare Utilization in Young and Middle-aged Adults. Medicine. 2016;95(2).

7.	Tian J, Venn AJ, Blizzard L, Patton GC, Dwyer T, Gall SL. Smoking status and health-related quality of life: a longitudinal study in young adults. Quality of Life Research. 2016;25(3):669-85. doi: 10.1007/s11136-015-1112-6.

8.	Virdis A, Giannarelli C, Fritsch Neves M, Taddei S, Ghiadoni L. Cigarette Smoking and Hypertension. Current Pharmaceutical Design. 2010;16(23):2518-25. doi: <http://dx.doi.org/10.2174/138161210792062920>.

9.	Hall JE, do Carmo JM, da Silva AA, Wang Z, Hall ME. Obesity-Induced Hypertension. Circulation Research. 2015;116(6):991-1006. doi: 10.1161/CIRCRESAHA.116.305697.

10.	Powell-Wiley TM, Poirier P, Burke LE, Després J-P, Gordon-Larsen P, Lavie CJ, et al. Obesity and Cardiovascular Disease: A Scientific Statement From the American Heart Association. Circulation. 2021;143(21):e984-e1010. doi: 10.1161/CIR.0000000000000973.

11.	Aune D, Schlesinger S, Norat T, Riboli E. Tobacco smoking and the risk of heart failure: A systematic review and meta-analysis of prospective studies. European Journal of Preventive Cardiology. 2019;26(3):279-88. doi: 10.1177/2047487318806658.

12.	VanderWeele TJ. Principles of confounder selection. European Journal of Epidemiology. 2019;34(3):211-9. doi: 10.1007/s10654-019-00494-6.

13.	Krupchanka D, Winkler P. State of mental healthcare systems in Eastern Europe: do we really understand what is going on? BJPsych International. 2016;13(4):96-9. Epub 2018/01/02. doi: 10.1192/S2056474000001446.

14.	Winkler P, Krupchanka D, Roberts T, Kondratova L, Machů V, Höschl C, et al. A blind spot on the global mental health map: a scoping review of 25 years' development of mental health care for people with severe mental illnesses in central and eastern Europe. The Lancet Psychiatry. 2017;4(8):634-42. doi: 10.1016/S2215-0366(17)30135-9.

15.	Pec O. Mental health reforms in the Czech Republic. BJPsych International. 2019;16(1):4-6. Epub 2018/03/15. doi: 10.1192/bji.2017.27.

16.	Broulikova HM, Dlouhy M, Winkler P. Expenditures on Mental Health Care in the Czech Republic in 2015. Psychiatric Quarterly. 2020;91(1):113-25. doi: 10.1007/s11126-019-09688-3.

17.	Nechanská B, Jann J, Nováková Z, Kudrna K, Slábová V, Pašingerová R. Psychiatric Care 2016. Prague: Insititute of Health Information and Statistics, 2017.

18.	Ministry of Health of the Czech Republic. Stratefy of the Reform of Psychiatric Care. 2013.

19.	Zhang J-j, Dong X, Liu G-h, Gao Y-d. Risk and Protective Factors for COVID-19 Morbidity, Severity, and Mortality. Clinical Reviews in Allergy & Immunology. 2023;64(1):90-107. doi: 10.1007/s12016-022-08921-5.

20.	Kerr S, Bedston S, Cezard G, Sampri A, Murphy S, Bradley DT, et al. Undervaccination and severe COVID-19 outcomes: meta-analysis of national cohort studies in England, Northern Ireland, Scotland, and Wales. The Lancet. 2024;403(10426):554-66. doi: 10.1016/S0140-6736(23)02467-4.

21.	Nab L, Parker EPK, Andrews CD, Hulme WJ, Fisher L, Morley J, et al. Changes in COVID-19-related mortality across key demographic and clinical subgroups in England from 2020 to 2022: a retrospective cohort study using the OpenSAFELY platform. The Lancet Public Health. 2023;8(5):e364-e77. doi: 10.1016/S2468-2667(23)00079-8.

22.	VanderWeele TJ, Shpitser I. A New Criterion for Confounder Selection. Biometrics. 2011;67(4):1406-13. doi: <https://doi.org/10.1111/j.1541-0420.2011.01619.x>.

23.	van Zwieten A, Tennant PWG, Kelly-Irving M, Blyth FM, Teixeira-Pinto A, Khalatbari-Soltani S. Avoiding overadjustment bias in social epidemiology through appropriate covariate selection: a primer. Journal of Clinical Epidemiology. 2022;149:127-36. doi: <https://doi.org/10.1016/j.jclinepi.2022.05.021>.

24.	Sawyer SM, Afifi RA, Bearinger LH, Blakemore S-J, Dick B, Ezeh AC, et al. Adolescence: a foundation for future health. The Lancet. 2012;379(9826):1630-40. doi: 10.1016/S0140-6736(12)60072-5.

25.	Kessler RC, Berglund P, Demler O, Jin R, Merikangas KR, Walters EE. Lifetime Prevalence and Age-of-Onset Distributions of DSM-IV Disorders in the National Comorbidity Survey Replication. Archives of General Psychiatry. 2005;62(6):593-602. doi: 10.1001/archpsyc.62.6.593.

26.	Pedersen CB, Mors O, Bertelsen A, Waltoft BL, Agerbo E, McGrath JJ, et al. A Comprehensive Nationwide Study of the Incidence Rate and Lifetime Risk for Treated Mental Disorders. JAMA Psychiatry. 2014;71(5):573-81. doi: 10.1001/jamapsychiatry.2014.16.

27.	Rosenbaum PR, Rubin DB. The Bias Due to Incomplete Matching. Biometrics. 1985;41(1):103-16. doi: 10.2307/2530647.

28.	Griffith GJ, Morris TT, Tudball MJ, Herbert A, Mancano G, Pike L, et al. Collider bias undermines our understanding of COVID-19 disease risk and severity. Nature Communications. 2020;11(1):5749. doi: 10.1038/s41467-020-19478-2.

29.	Lin M, Lucas HC, Shmueli G. Too Big to Fail: Large Samples and the p-Value Problem. Information Systems Research. 2013;24(4):906-17. doi: 10.1287/isre.2013.0480.

30.	Pillinger T, McCutcheon RA, Vano L, Mizuno Y, Arumuham A, Hindley G, et al. Comparative effects of 18 antipsychotics on metabolic function in patients with schizophrenia, predictors of metabolic dysregulation, and association with psychopathology: a systematic review and network meta-analysis. The Lancet Psychiatry. 2020;7(1):64-77. doi: <https://doi.org/10.1016/S2215-0366(19)30416-X>.

31.	Taipale H, Tanskanen A, Mehtälä J, Vattulainen P, Correll CU, Tiihonen J. 20-year follow-up study of physical morbidity and mortality in relationship to antipsychotic treatment in a nationwide cohort of 62,250 patients with schizophrenia (FIN20). World Psychiatry. 2020;19(1):61-8. doi: <https://doi.org/10.1002/wps.20699>.

32.	Tiihonen J, Suokas JT, Suvisaari JM, Haukka J, Korhonen P. Polypharmacy With Antipsychotics, Antidepressants, or Benzodiazepines and Mortality in Schizophrenia. Archives of General Psychiatry. 2012;69(5):476-83. doi: 10.1001/archgenpsychiatry.2011.1532.

33.	Sariaslan A, Sharpe M, Larsson H, Wolf A, Lichtenstein P, Fazel S. Psychiatric comorbidity and risk of premature mortality and suicide among those with chronic respiratory diseases, cardiovascular diseases, and diabetes in Sweden: A nationwide matched cohort study of over 1 million patients and their unaffected siblings. PLOS Medicine. 2022;19(1):e1003864. doi: 10.1371/journal.pmed.1003864.

34.	Austin PC. A comparison of 12 algorithms for matching on the propensity score. Statistics in Medicine. 2014;33(6):1057-69. doi: <https://doi.org/10.1002/sim.6004>.

35.	Greifer N, Stuart EA. Matching Methods for Confounder Adjustment: An Addition to the Epidemiologist’s Toolbox. Epidemiologic Reviews. 2021;43(1):118-29. doi: 10.1093/epirev/mxab003.

36.	Stuart EA. Matching Methods for Causal Inference: A Review and a Look Forward. Statistical Science. 2010;25(1):1-21. doi: 10.1214/09-STS313.

37.	VanderWeele TJ, Ding P. Sensitivity Analysis in Observational Research: Introducing the E-Value. Annals of Internal Medicine. 2017;167(4):268-74. doi: 10.7326/M16-2607.

38.	Wang Q, Xu R, Volkow ND. Increased risk of COVID-19 infection and mortality in people with mental disorders: analysis from electronic health records in the United States. World Psychiatry. 2021;20(1):124-30. doi: <https://doi.org/10.1002/wps.20806>.

39.	Nishimi K, Neylan TC, Bertenthal D, Seal KH, O’Donovan A. Association of Psychiatric Disorders With Incidence of SARS-CoV-2 Breakthrough Infection Among Vaccinated Adults. JAMA Network Open. 2022;5(4):e227287-e. doi: 10.1001/jamanetworkopen.2022.7287.

40.	Blom JD, Martínez-González MÁ, Molendijk ML, Molero P, Reina G, Reinken A, et al. COVID-19 risk, course and outcome in people with mental disorders: a systematic review and meta-analyses. Epidemiology and Psychiatric Sciences. 2023;32:e61. Epub 2023/10/20. doi: 10.1017/S2045796023000719.

41.	Teixeira AL, Krause TM, Ghosh L, Shahani L, Machado-Vieira R, Lane SD, et al. Analysis of COVID-19 Infection and Mortality Among Patients With Psychiatric Disorders, 2020. JAMA Network Open. 2021;4(11):e2134969-e. doi: 10.1001/jamanetworkopen.2021.34969.

42.	Jeon H-L, Kwon JS, Park S-H, Shin J-Y. Association of mental disorders with SARS-CoV-2 infection and severe health outcomes: nationwide cohort study. The British Journal of Psychiatry. 2021;218(6):344-51. Epub 2021/01/07. doi: 10.1192/bjp.2020.251.

43.	Yang H, Chen W, Hu Y, Chen Y, Zeng Y, Sun Y, et al. Pre-pandemic psychiatric disorders and risk of COVID-19: a UK Biobank cohort analysis. The Lancet Healthy Longevity. 2020;1(2):e69-e79. doi: 10.1016/S2666-7568(20)30013-1.

44.	Goldberger N, Bergman-Levy T, Haklai Z, Yoffe R, Davidson M, Susser E, et al. COVID-19 and severe mental illness in Israel: testing, infection, hospitalization, mortality and vaccination rates in a countrywide study. Molecular Psychiatry. 2022;27(7):3107-14. doi: 10.1038/s41380-022-01562-2.

45.	Nemani K, Li C, Olfson M, Blessing EM, Razavian N, Chen J, et al. Association of Psychiatric Disorders With Mortality Among Patients With COVID-19. JAMA Psychiatry. 2021;78(4):380-6. doi: 10.1001/jamapsychiatry.2020.4442.

46.	Ranger TA, Clift AK, Patone M, Coupland CAC, Hatch R, Thomas K, et al. Preexisting Neuropsychiatric Conditions and Associated Risk of Severe COVID-19 Infection and Other Acute Respiratory Infections. JAMA Psychiatry. 2022. doi: 10.1001/jamapsychiatry.2022.3614.

47.	Gibbs A, Maripuu M, Öhlund L, Widerström M, Nilsson N, Werneke U. COVID-19-associated mortality in individuals with serious mental disorders in Sweden during the first two years of the pandemic– a population-based register study. BMC Psychiatry. 2024;24(1):189. doi: 10.1186/s12888-024-05629-y.

48.	Descamps A, Frenkiel J, Zarca K, Laidi C, Godin O, Launay O, et al. Association between mental disorders and COVID-19 outcomes among inpatients in France: A retrospective nationwide population-based study. Journal of Psychiatric Research. 2022;155:194-201. doi: <https://doi.org/10.1016/j.jpsychires.2022.08.019>.

49.	Allen B, El Shahawy O, Rogers ES, Hochman S, Khan MR, Krawczyk N. Association of substance use disorders and drug overdose with adverse COVID-19 outcomes in New York City: January–October 2020. Journal of Public Health. 2021;43(3):462-5. doi: 10.1093/pubmed/fdaa241.

50.	Wang QQ, Kaelber DC, Xu R, Volkow ND. COVID-19 risk and outcomes in patients with substance use disorders: analyses from electronic health records in the United States. Molecular Psychiatry. 2021;26(1):30-9. doi: 10.1038/s41380-020-00880-7.

51.	Wen-Jan T, Hailey MK, Robert PL. Assessing the risk of COVID-19 reinfection and severe outcomes among individuals with substance use disorders: a retrospective study using real-world electronic health records. BMJ Open. 2023;13(12):e074993. doi: 10.1136/bmjopen-2023-074993.

52.	Schwarzinger M, Luchini S, Teschl M, Alla F, Mallet V, Rehm J. Mental disorders, COVID-19-related life-saving measures and mortality in France: A nationwide cohort study. PLOS Medicine. 2023;20(2):e1004134. doi: 10.1371/journal.pmed.1004134.

53.	Lee D-W, Bae YS, Lee J-R, Sohn JH, Lee H, Lee JY. COVID-19 vaccination, incidence, and mortality rates among individuals with mental disorders in South Korea: A nationwide retrospective study. Asian Journal of Psychiatry. 2023;85:103600. doi: <https://doi.org/10.1016/j.ajp.2023.103600>.

54.	Bertolini F, Witteveen AB, Young S, Cuijpers P, Ayuso-Mateos JL, Barbui C, et al. Risk of SARS-CoV-2 infection, severe COVID-19 illness and COVID-19 mortality in people with pre-existing mental disorders: an umbrella review. BMC Psychiatry. 2023;23(1):181. doi: 10.1186/s12888-023-04641-y.

55.	Komenda M, Panoška P, Bulhart V, Žofka J, Brauner T, Hak J, et al. COVID-19: Overview of the Current Situation in Czechia Prague: Ministry of Health of the Czech Republic; [cited 2024 10th April]. Available from: <https://onemocneni-aktualne.mzcr.cz/covid-19>.

56.	Ministry of Health of the Czech Republic. COVID-19 Vaccination Strategy in Czech Republic. 2020.

57.	Bryndová L, Šlegerová L, Votápková J, Hrobonˇ P, Shuftan N, Spranger A. Czechia: Health System Review. 2023.

58.	Wasserstein RL, Lazar NA. The ASA Statement on p-Values: Context, Process, and Purpose. The American Statistician. 2016;70(2):129-33. doi: 10.1080/00031305.2016.1154108.
