# Supplementary material for: Deaths with COVID-19 and from all-causes following first-ever SARS-CoV-2 infection in individuals with preexisting mental disorders: A national cohort study from Czechia
Source: PLoS Med. 2024 Jul 15;21(7):e1004422. doi: 10.1371/journal.pmed.1004422 (PMC11285938; doi:10.1371/journal.pmed.1004422)
Supplement: Attachment — Submitted filename: Authors_reply.docx [file pmed.1004422.s021.docx]

**Responses to Editorial and Reviewer Comments**

We would like to reiterate our gratitude to the editors and reviewers for their time in appraising our manuscript. The detailed and thoughtful feedback have helped us to further improve our work. Below, we present a point-by-point list of the remaining editorial and reviewer comments alongside our responses. We submit two versions of the amended manuscript; one clean copy and a second annotated copy with tracked changes. The documents also include page and line numbers, which we refer to in the comments below.

**Editorial Comments**

**Point 1:** “We kindly ask you to reduce the number of supplementary figures and combining multiple graphs into one figure, for example, one figure showing all graphs for "28 days post-infection, epochs 1 through 5, affective disorders, diagnosed and treated". We do not have a formal limit on supplemental display items, but we feel that more than 200 figures is excessive for readers to navigate.”

**Reply:** Thank you for this comment. Each figure refers to one specific model, and we have 200 models overall. It is highly recommended to report these since they provide a number of additional statistics and allow to assess the presence of certain biases [1]. Given the structure of these graphs (i.e., the curves and the tables), we do not see a viable option to combine multiple ones together, thus we respectfully decided to retain all 200 graphs.

**Point 2:** “When reporting statistical information, please use commas to separate upper and lower bounds, as opposed to hyphens as these can be confused with reporting of negative values.”

**Reply:** Thank you for this comment. We have now reported the results accordingly throughout the manuscript.

**Point 3:** “l.55: Please simplify the description of the database. We suggest changing “We used Czech national, whole population, all healthcare encompassing register-based data…” to “We used population-level data from a Czech healthcare registry…” (or similar).”

**Reply:** Thank you for this comment. We have now provided this information as follows (page 2 line 53-56):

- “We used data from Czech national health registers to identify first-ever serologically-confirmed SARS‑CoV‑2 infections in five epochs related to different phases of the pandemic: 1^st^ March 2020-30^th^ September 2020, 1^st^ October 2020-26^th^ December 2020, 27^th^ December 2020-31^st^ March 2021, 1^st^ April 2021-31^st^ October 2021, and 1^st^ November 2021-29^th^ February 2022.”.

**Point 4:** “l.56: Please include the time frames of the five epochs.”

**Reply:** Thank you for this comment. We have now provided this information as follows (page 2 line 53-56):

- “We used data from the Czech national health registers to identify first-ever serologically-confirmed SARS‑CoV‑2 infections in five epochs related to different phases of the pandemic: 1^st^ March 2020-30^th^ September 2020, 1^st^ October 2020-26^th^ December 2020, 27^th^ December 2020-31^st^ March 2021, 1^st^ April 2021-31^st^ October 2021, and 1^st^ November 2021-29^th^ February 2022.”.

**Point 5:** “l.58: Please define ‘ICD’ at first use.”

**Reply:** Thank you for this comment. We have now provided this information as follows (page 2 line 56-62):

- “In these people, we ascertained cases of mental disorders using two approaches: (1) per the International Classification of Diseases 10^th^ Revision (ICD-10) diagnostic codes for substance use, psychotic, affective, and anxiety disorders and (2) per ICD-10 diagnostic codes for the above mental disorders coupled with a prescription for anxiolytics/hypnotics/sedatives, antidepressants, antipsychotics or stimulants per the Anatomical Therapeutic Chemical (ATC) classification codes.”.

**Point 6:** “l.61: Please define ‘ATC’ at first use”

**Reply:** Thank you for this comment. Thank you for this comment. We have now provided this information as follows (page 2 line 56-62):

- “In these people, we ascertained cases of mental disorders using two approaches: (1) per the International Classification of Diseases 10^th^ Revision (ICD-10) diagnostic codes for substance use, psychotic, affective, and anxiety disorders and (2) per ICD-10 diagnostic codes for the above mental disorders coupled with a prescription for anxiolytics/hypnotics/sedatives, antidepressants, antipsychotics or stimulants per the Anatomical Therapeutic Chemical (ATC) classification codes.”.

**Point 7:** “l.61: Please remove the word ‘exact’ (“We matched…").”

**Reply:** Thank you for this comment. We have now adjusted this throughout the manuscript.

**Point 8:** “ll.63-64: Please change to “death with COVID-19” (please edit this consistently throughout the paper).”

**Reply:** Thank you for this comment. We have now used “deaths with COVID-19” when referring to the outcome involving the codes U071 and U072 listed as a cause of death on the death certificate throughout the manuscript.

**Point 9:** “l.66: Please briefly state the number of people included in the study together with their demographics (age, sex etc.).”

**Reply:** Thank you for this comment. Since the cohort sizes and the distributions per age and sex vary across epoch-mental disorder combinations, we have followed the reporting style we used in the main text to provide these as follows (page 2 line 67-72):

- “The number of individuals in matched-cohorts ranged from 1,328 in epoch 1 to 854,079 in epoch 5. The proportion of females ranged from 34.98% in people diagnosed with substance use disorders in epoch 3 to 71.16% in individuals diagnosed and treated with anxiety disorders in epoch 5. The mean age ranged from 40.97 years (standard deviation [SD] = 15.69 years) in individuals with substance use disorders in epoch 5 to 56.04 years (SD = 18.37 years) in people with psychotic disorders in epoch 2.”.

**Point 10:** “l.67: As above “dying from COVID-19” – Is it 100% certain that these people died from COVID-19 or was it possibly death with COVID-19?”

**Reply:** Thank you for this comment. We have now used “deaths with COVID-19” when referring to the outcome involving the codes U071 and U072 listed as a cause of death on the death certificate throughout the manuscript.

**Point 11:** “ll.68 ff: Please present the values for each of the epochs instead of using a “from..to” format. Please revise throughout the entire Abstract.”

**Reply:** Thank you for this comment. We explored the possibility of including each data value per epoch-mental disorders combinations; however, the number of these is so large (e.g., 16 data values exclusively for psychotic disorders) that the Abstract becomes effectively unreadable. For these reasons, we would like to respectfully retain our original reporting style in the Abstract. Nevertheless, we have now followed other suggestions from the editors and adjusted the reporting of results in the Abstract as follows (page 2 line 72-90):

- “People diagnosed with or diagnosed and treated for psychotic disorders had a consistently elevated risk of dying with COVID-19 in epochs 2, 3, 4, and 5, with adjusted hazard ratios (aHR) ranging from 1.46 [95% confidence intervals (CIs), 1.18, 1.79] to 1.93 [95% CIs, 1.12, 3.32]. This patient group demonstrated also a consistently elevated risk of all-cause mortality in epochs 2, 3, 4, and 5 (aHR from 1.43 [95% CIs, 1.23, 1.66] to 1.99 [95% CIs, 1.25, 3.16]). The models could not be reliably fit for psychotic disorders in epoch 1. People diagnosed with substance use disorders had an increased risk of all-cause mortality 28 days post-infection in epoch 3, 4, and 5 (aHR from 1.30 [95% CIs, 1.14, 1.47] to 1.59 [95% CIs, 1.19, 2.12]) and 60 days post-infection in epoch 2, 3, 4, and 5 (aHR from 1.22 [95% CIs, 1.08, 1.38] to 1.52 [95% CIs, 1.16, 1.98]). Cases ascertained based on diagnosis of substance use disorders and treatment had increased risk of all-cause mortality in epoch 2, 3, 4, and 5 (aHR from 1.22 [95% CIs, 1.03, 1.43] to 1.91 [95% CIs, 1.25, 2.91]). The models could not be reliably fit for substance use disorders in epoch 1. In contrast to these, people diagnosed with anxiety disorders had a decreased risk of death with COVID-19 in epoch 2, 3, and 5 (aHR from 0.78 [95% CIs, 0.69, 0.88] to 0.89 [95% CIs, 0.81, 0.98]) and all-cause mortality in epoch 2, 3, 4, and 5 (aHR from 0.83 [95% CIs, 0.77, 0.90] to 0.88 [95% CIs, 0.83, 0.93]). People diagnosed and treated for affective disorders had a decreased risk of both death with COVID-19 and from all-causes in epoch 3 (aHR from 0.87 [95% CIs, 0.79, 0.96] to 0.90 [95% CIs, 0.83, 0.99]), but demonstrated broadly null effects in other epochs.”.

**Point 12:** “l.77: “COVID-19 death” – Please change to “death with COVID-19”.”

**Reply:** Thank you for this comment. We have now used “deaths with COVID-19” when referring to the outcome involving the codes U071 and U072 listed as a cause of death on the death certificate throughout the manuscript.

**Point 13:** “l.80: Please change to “death with COVID-19”.

**Reply:** Thank you for this comment. We have now used “deaths with COVID-19” when referring to the outcome involving the codes U071 and U072 listed as a cause of death on the death certificate throughout the manuscript.

**Point 14:** “ll.80-81: “all-cased death” – please revise.”

**Reply:** Thank you for this comment, we have now corrected this.

**Point 15:** “In the last sentence of the Abstract Methods and Findings section, please describe the main limitation(s) of the study's methodology.”

**Reply:** Thank you for this comment. We have now provided this information as follows (page 2-3 line 90-93):

- “Given the unavailability of data on a number of potentially influential confounders, particularly body mass index, tobacco smoking status, and socioeconomic status, part of the detected associations might be due to residual confounding.”.

**Point 16:** “ll.92-93: Please change to: “Existing research has demonstrated consistently elevated risk of death with COVID-19 or all-cause mortality in people..”. Please revise throughout the entire Author Summary.”

**Reply:** Thank you for this comment. We have now used “deaths with COVID-19” when referring to the outcome involving the codes U071 and U072 listed as a cause of death on the death certificate throughout the manuscript.

**Point 17:** “ll.112-113: Please change to: “These associations could not be fully explained by differences in vaccination uptake or clinically-recorded physical comorbidities.”.”

**Reply:** Thank you for this comment, we have now corrected this.

**Point 18:** “In the final bullet point of ‘What Do These Findings Mean?’, please describe the main limitations of the study in non-technical language.”

**Reply:** Thank you for this comment. We have now provided this as follows (page 4 line 151-155):

- “The main limitation of the present study was its inability to fully control for a number of characteristics, particularly body mass index, tobacco smoking status, and socioeconomic status, that might confound the associations between mental disorders and mortality: future studies should explore these associations while accounting for these confounders.”.

**Point 19:** “ll.185-186: The following sentence is unclear: “The Czech National Vaccination Strategy was launched in December 2020, and it considered no pre-existing mental disorders as reason for priority inoculation [32].” Does this mean it did not consider pre-existing mental disorders as a reason for priority inoculation (as written, it suggests that people without preexisting mental disorders were given priority)? Please revise for clarity.”

**Reply:** Thank you for this comment. The Czech vaccination strategy did not, in fact, include mental disorders as reason for priority inoculation. We have now provided this information more explicitly as follows (page 6 line 217-219):

- “The Czech National Vaccination Strategy was launched in December 2020, with pre-existing mental disorders not considered as reason for priority inoculation [2].”.

**Point 20:** “l.199: “The register covers nearly the entire Czech population.” – Is it possible to provide actual numbers here?”

**Reply:** Thank you for this comment. The healthcare system in Czechia is based on the compulsory health insurance model [3]; thus, the coverage of registers is essentially equal to the population size. We have now included this information as follows (page 6 line 231-232):

- “The register covers nearly the entire Czech population (approximately 10.7 million inhabitants).”.

**Point 21:** “l.211: Should this be “IHIS” instead of “NHIS”?”

**Reply:** Thank you for this comment. Data from the National Registry of Reimbursed Health Services (NRRHS) are part of the Czech National Health Information System (NHIS). We have now provided this information as follows (page 6 line 229-231):

- “We used data from the National Registry of Reimbursed Health Services (NRRHS), part of the National Health Information System (NHIS), covering inpatient and outpatient services, including primary care, as well as prescription medications.”.

**Point 22:** “l.260: Please change “exactly-matched” to “matched” (also at l.497).”

**Reply:** Thank you for this comment. We have now adjusted this throughout the manuscript.

**Point 23:** “ll.262-263: Please change “Since vaccination do not confer…” to “Since vaccination does not confer…””

**Reply:** Thank you for this comment, we have now corrected this.

**Point 24:** “ll.264-266: Please change to “For instance, when an individual received the first dose of a two-dose regimen more than 14 days before the infection, and the second dose 14 or less days before the infection…””

**Reply:** Thank you for this comment, we have now corrected this.

**Point 25:** “l.285 ff: "We considered (1) death due to COVID-19 (ICD-10 codes U071 and U072)." - We feel that it should be made clear to the reader that these codes do not identify the cause of death, but rather that a person was diagnosed with COVID-19 with or without the virus identified. Therefore, we feel that "Death with COVID-19" (or e.g. “death in a person diagnosed with COVID-19”) along with an explanation that other underlying causes may have been the actual cause of the death would be a more appropriate description. While we understand that you are trying to rule out competing causes of death (as a sensitivity analysis), we feel that the description needs to be much clearer. Is there an entry on death certificates that states U071 or U072 in a specific cause of death field?”

**Reply:** Thank you for this important comment. Those individuals we considered as to have died due to COVID-19 had the U071 and U072 ICD-10 diagnostic codes listed as a cause of death on their death certificates. However, COVID-19 may lead to multi(organ) failure, particularly in those with pre-existing comorbidities, and we did not consider specifically those individuals who had COVID-19 listed as their sole cause of death. Thus, we agree with the editors to take a cautious approach and refer to deaths with COVID-19 throughout the manuscript. We have now also included that the U071 and U072 diagnostic codes were listed as a cause of death on the death certificate as follows (page 8 line 317-319):

- “We considered (1) death with COVID-19 (ICD-10 codes U071 and U072 listed as a cause of death on the death certificate) and (2) all-cause mortality occurring in the period of (1) 28 days and (2) 60 days after a positive test for SARS‑CoV‑2.”.

**Point 26:** “l.285 ff: Given that the ICD-10 code "U072" is used to identify an individual whose COVID-19 disease has not yet been confirmed by laboratory testing, we wonder how you have calculated the time period for individuals without a COVID-19 diagnosis (i.e., when does the time period begin)?

**Reply:** Thank you for this comment. To be present in our cohorts, an individual must have tested positively for a SARS-CoV-2 infection. Thus, the follow-up for each individual was the time from the positive test to either the outcome, here defined as death with COVID-19 using the U071 and U072 ICD-10 diagnostic codes listed on death certificates or all-cause mortality, or the end of the follow-up period.

Since the U071 and U072 diagnostic codes were introduced during a major health emergency, we wanted to account also for potential uncertainty in coding. Thus, to maximize the coverage of relevant cases, we included also the U072 diagnostic code to identify those who died with COVID-19.

**Point 27:** “l.335: Please define ‘SD’ at first use.”

**Reply:** Thank you for this comment, we have now corrected this.

**Point 28:** “l.340: Please change to: “Risk of COVID-19 Related Death in People with Pre-existing Mental Disorders””

**Reply:** Thank you for this comment. We have now used “deaths with COVID-19” when referring to the outcome involving the codes U071 and U072 listed as a cause of death on the death certificate throughout the manuscript.

**Point 29:** “l.341 ff: Please refrain from writing “COVID-19 death”, but use “COVID-19 related death” or similar. Please revise throughout the main text.”

**Reply:** Thank you for this comment. We have now used “deaths with COVID-19” when referring to the outcome involving the codes U071 and U072 listed as a cause of death on the death certificate throughout the manuscript.

**Point 30:** “l.341 ff: We feel it could be misleading to only present values from a specific epoch. We therefore suggest either presenting all values for the epochs discussed or removing the values and referring to the figure and/or table where readers may find the values in full. For readability, we prefer referring to the relevant figures and/or tables. Please revise throughout the manuscript.”

**Reply:** Thank you for this comment. We have now followed this suggestion and removed all data values from the main text as follows:

- “In the models adjusting for all considered confounders, including vaccination uptake and clinically-recorded physical comorbidities, people diagnosed with or diagnosed and treated for psychotic disorders had an elevated risk of death with COVID-19 in epochs 2, 3, 4, and 5, both 28 and 60 following SARS‑CoV‑2 infection. The models could not be reliably fit for psychotic disorders in epoch 1. Those diagnosed with substance use disorders had an increased risk of death with COVID-19 28 days post-infection in epoch 3 and 4 and 60 days post-infection in epoch 3. Cases ascertained based on diagnosis of substance use disorders and treatment by psychopharmaceuticals had an elevated risk of death with COVID-19 in epoch 3, both 28 and 60 days following infection. The models could not be reliably fit for substance use disorders in epoch 1, and the remaining ones were consistent with a null effect.

In contrast, people diagnosed with or diagnosed and treated for anxiety disorders had a decreased risk of death with COVID-19 in epoch 2, 3, and 5, both 28 and 60 days post-infection. The remaining models for anxiety disorders were consistent with a null effect. Additionally, people diagnosed and treated for affective disorders had a decreased risk of death with COVID-19 in epoch 3, both 28 and 60 days post-infection, but all other models involving affective disorders were broadly consistent with a null effect. The results for any studied mental disorder were – regardless of case ascertainment definition – consistent with a null effect in all epochs. For detailed results see Figure 1, Supplementary Table 8-10, and Supplementary Figure 2-101.” (page 9 line 381-400).

- “In the models adjusting for all considered confounders, including vaccination uptake and clinically-recorded physical comorbidities, people diagnosed with or diagnosed and treated for psychotic disorders were more likely to die in epochs 2, 3, 4, and 5, both 28 and 60 days post-infection. The models could not be reliably fit for psychotic disorders in epoch 1. In those diagnosed with substance use disorders, there was an elevated risk of all-cause mortality 28 days post-infection in epoch 3, 4, and 5 and 60 days post-infection in epoch 2, 3, 4, and 5. Cases ascertained based on diagnosis of substance use disorders and treatment had increased risk of all-cause mortality in epoch 2, 3, 4, and 5, both 28 and 60 days post-infection. The models could not be reliably fit for substance use disorders in epoch 1, and the remaining ones were consistent with a null effect.

Conversely, people diagnosed with anxiety disorders had a decreased risk of all-cause mortality in epoch 2, 3, 4, and 5, both 28 and 60 days post-infection. Cases ascertained based on diagnosis of anxiety disorders and treatment by psychopharmaceuticals demonstrated broadly consistent results, with decreased risks of all-cause mortality in epoch 2, 3, and 5, both 28 and 60 days post-infection. The remaining models for anxiety disorders were consistent with a null effect. In addition, people diagnosed and treated for affective disorders had a decreased risk of all-cause death in epoch 3, both 28 and 60 following SARS‑CoV‑2 infection, but all other models involving affective disorders were broadly consistent with a null effect. The results for any studied mental disorder were – regardless of case ascertainment definition – consistent with a null effect in all epochs. For detailed results see Figure 2, Supplementary Table 11-13, and Supplementary Figure 102-201.” (page 9-10 line 403-424).

**Point 31:** “l.343: Please clearly describe which epochs were covered for the different disorders. For example, you write "in each of the studied epochs," which may lead readers to think of all five epochs, whereas for psychotic disorders only epochs 2, 3, 4, and 5 were covered in the analysis.”

**Reply:** Thank you for this comment. We have now explicitly listed all epochs not consistent with a null effect and included information on the epoch-mental disorder combinations for which the models could be reliable fit as follows:

- “In the models adjusting for all considered confounders, including vaccination uptake and clinically-recorded physical comorbidities, people diagnosed with or diagnosed and treated for psychotic disorders had an elevated risk of death with COVID-19 in epochs 2, 3, 4, and 5, both 28 and 60 following SARS‑CoV‑2 infection. The models could not be reliably fit for substance use disorders in epoch 1. Those diagnosed with substance use disorders had an increased risk of all-cause mortality 28 days post-infection in epoch 3 and 4 and 60 days post-infection in epoch 3. Cases ascertained based on diagnosis of substance use disorders and treatment by psychopharmaceuticals had an elevated risk of death with COVID-19 in epoch 3, both 28 and 60 days following infection. The models could not be reliably fit for substance use disorders in epoch 1, and the remaining ones were consistent with a null effect.

In contrast, people diagnosed with or diagnosed and treated for anxiety disorders had a decreased risk of death with COVID-19 in epoch 2, 3, and 5, both 28 and 60 days post-infection. The remaining models for anxiety disorders were consistent with a null effect. Additionally, people diagnosed and treated for affective disorders had a decreased risk of death with COVID-19 in epoch 3, both 28 and 60 days post-infection, but all other models involving affective disorders were broadly consistent with a null effect. The results for any studied mental disorder were – regardless of case ascertainment definition – consistent with a null effect in all epochs. For detailed results see Figure 1, Supplementary Table 8-10, and Supplementary Figure 2-101.” (page 9 line 381-400).

- “In the models adjusting for all considered confounders, including vaccination uptake and clinically-recorded physical comorbidities, people diagnosed with or diagnosed and treated for psychotic disorders were more likely to die in epochs 2, 3, 4, and 5, both 28 and 60 days post-infection. The models could not be reliably fit for substance use disorders in epoch 1. In those diagnosed with substance use disorders, there was an elevated risk of all-cause mortality 28 days post-infection in epoch 3, 4, and 5 and 60 days post-infection in epoch 2, 3, 4, and 5. Cases ascertained based on diagnosis of substance use disorders and treatment had increased risk of all-cause mortality in epoch 2, 3, 4, and 5, both 28 and 60 days post-infection. The models could not be reliably fit for substance use disorders in epoch 1, and the remaining ones were consistent with a null effect.

Conversely, people diagnosed with anxiety disorders had a decreased risk of all-cause mortality in epoch 2, 3, 4, and 5, both 28 and 60 days post-infection. Cases ascertained based on diagnosis of anxiety disorders and treatment by psychopharmaceuticals demonstrated broadly consistent results, with decreased risks of all-cause mortality in epoch 2, 3, and 5, both 28 and 60 days post-infection. The remaining models for anxiety disorders were consistent with a null effect. In addition, people diagnosed and treated for affective disorders had a decreased risk of all-cause death in epoch 3, both 28 and 60 following SARS‑CoV‑2 infection, but all other models involving affective disorders were broadly consistent with a null effect. The results for any studied mental disorder were – regardless of case ascertainment definition – consistent with a null effect in all epochs. For detailed results see Figure 2, Supplementary Table 11-13, and Supplementary Figure 102-201.” (page 9-10 line 403-424).

**Point 32:** “l.345 ff: Please ensure to include the relevant statistical details in each pair of parenthesis (e.g. l.345 “..and 60 days (aHR 1.46 [95% CIs, 1.20-1.79] to 1.93 [95% CIs, 1.14-3.27]) following..”). Please revise throughout the main text.”

**Reply:** Thank you for this comment. Following the editors’ suggestion, we have now removed the reporting of data values in the main text.

**Point 33:** “l.390 ff: We find the presentation of the sensitivity analysis data difficult to understand. As above, we suggest either presenting the data values in full or referring to a relevant table and/or figure where the data can be seen in full. In addition, we feel that the description of the sensitivity analyses in the supplementary material should be incorporated into the main text (ll.308-320), as it is not significantly longer and provides important details, particularly about the selected negative control characteristics. Please also briefly explain how the theoretical maximum of 40 is calculated.”

**Reply:** Thank you for this comment. The theoretical maximum of 40 tests per one epoch-mental disorder combination reflects the fact that the presence of each mental disorder was defined using two approaches (i.e., ICD-10 codes or ICD-10 codes and ATC codes), and there were always five negative control exposures and four outcomes (i.e., deaths with COVID-19 and all-cause mortality occurring in 28 and 60 days following the infection).

We have now elaborated on the methodology of negative control exposure analyses as follows (page 8-9 line 342-360):

- “Having a history of a mental disorder might influence the risk of being tested for SARS-CoV-2 infection; thus, restricting the analysis to individuals who had a positive test might lead to collider bias [4]. To examine potential presence of collider bias, we conducted negative control exposure analyses by assessing the associations between the characteristics that are expected to be unrelated to the outcome and the outcome itself within the chosen cohorts [4]. To do so, we considered the occurrence of (1) migraine (ICD-10 code G43), (2) fracture of forearm (ICD-10 code S52), (3) acne (ICD-10 code L70), (4) mild allergies (ICD-10 codes J301, L500 and L23), and (5) transport accidents (ICD-10 codes V01-V99) in the time period of five years prior to the positive SARS-CoV-2 test. We assessed the occurrence of each of these separately. Then, we fitted stratified Cox proportional hazards models with the above characteristics being the exposures, while using the confounders and outcomes from the main analysis. We reported the total number and proportion of non-null tests, with the theoretical maximum being 40 (or 100%) per one epoch-mental disorder combination (i.e., two case ascertainment definitions X five negative control exposures X four outcomes). Since some of our cohorts were considerably large (i.e., anxiety disorders in epochs 2 to 5), it would be possible to have non-null results even if the effect sizes were negligible [5]. Thus, we complementarily provided averaged HRs across the 40 tests per one epoch-mental disorder combination. Proportion of non-null tests closer to 0% and/or averaged HRs closer to 1 would suggest the absence of collider bias.”.

Then, we have now simplified the reporting of the results of negative control exposure analyses as follows (page 10 line 427-431):

- “In negative control exposure analyses, the proportion of non-null tests did not exceed 15% for substance use disorders, 15% for psychotic disorders, 20% for affective disorders, 40% for anxiety disorders, and 45% for any of the studied mental disorders. Proportion of non-null tests closer to 0% increases the confidence in the lack of collider bias. See details, including averaged HRs, in Supplementary Table 14.”.

**Point 34:** “Please remove any subheadings.”

**Reply:** Thank you for this comment. We have now removed the subheadings from the Discussion.

**Point 35:** “ll.491-493: Please change to “Strengths included..”. We suggest describing the strengths of the study in more detail as currently the description “in a complex and detailed way” is rather vague.”

**Reply:** Thank you for this comment. We have now elaborated these as follows (page 12 line 523-528):

- “Strengths included the use of national, whole population, fully standardized data on SARS‑CoV‑2 infection status, healthcare utilization, and mortality. Next, we investigated the robustness of our results through using multiple definitions of the exposure and the outcome. Then, we used negative control exposures to explore the potential presence of collider bias and E-values to establish what level of unaccounted for confounding would explain away the observed associations.”.

**Point 36:** “Table 1/2: Please ensure to define all abbreviations used in the table below the table (SD, Inf., IQR, ICD, ATC). Please mention in a footnote the relevant ICD-10 codes for the different groups as well as what “Comorbidities” encompasses. Please define “exposed” and “unexposed”.”

**Reply:** Thank you for this comment. We have now included this additional information in the manuscript as follows:

- “The results are presented as absolute numbers (n) with proportions (%), means with standard deviations (SD), and medians with interquartile ranges (IQR). The time frames for epochs were: (1) 1^st^ March 2020-30^th^ September 2020 for epoch 1, (2) 1^st^ October 2020-26^th^ December 2020 for epoch 2, (3) 27^th^ December 2020-31^st^ March 2021 for epoch 3, (4) 1^st^ April 2021-31^st^ October 2021 for epoch 4, and (5) 1^st^ November 2021-29^th^ February 2022 for epoch 5. “Exposed” and “unexposed” refer to people with the respective mental disorder and their matched counterparts without that mental disorder, respectively. The International Classification of Diseases 10^th^ Revision (ICD-10) diagnostic codes were (1) F10-F19, F20-F29, F30-F39, F40-F48 for any mental disorder, (2) F10-F19 for substance use disorders, (3) F20-F29 for psychotic disorders, (4) F30-F39 for affective disorders, and (5) F40-F48 for anxiety disorders.”.

**Point 37:** “Figure 1/2: In the figure description, please explain the time frame of the different epochs and provide the relevant ICD-10 codes for the different groups. Please ensure to define all abbreviations used (ICD, ATC).”

**Reply:** Thank you for this comment. We have now included this additional information in the manuscript as follows:

- “All results are expressed as hazard ratios with 95% confidence intervals. The models were adjusted for matching variables and all additional confounders. The time frames for epochs were: (1) 1^st^ March 2020-30^th^ September 2020 for epoch 1, (2) 1^st^ October 2020-26^th^ December 2020 for epoch 2, (3) 27^th^ December 2020-31^st^ March 2021 for epoch 3, (4) 1^st^ April 2021-31^st^ October 2021 for epoch 4, and (5) 1^st^ November 2021-29^th^ February 2022 for epoch 5. “Diagnosed” refers to cases ascertained by diagnosis per the International Classification of Diseases 10^th^ Revision (ICD-10) diagnostic codes: (1) F10-F19, F20-F29, F30-F39, F40-F48 for any mental disorder, (2) F10-F19 for substance use disorders, (3) F20-F29 for psychotic disorders, (4) F30-F39 for affective disorders, and (5) F40-F48 for anxiety disorders. “Diagnosed and treated” refers to cases ascertained by diagnosis per the above ICD-10 codes **coupled with prescription for** anxiolytics/hypnotics/sedatives (N05B, N05C), (2) antidepressants (N06A), (3) antipsychotics (N05A) or (4) stimulants (N06B) per the Anatomical Therapeutic Chemical (ATC) classification codes. The models could not be reliably fit for substance use and psychotic disorders in epoch 1.**”.**

**Point 38:** “Please be sure to revise the supplementary figures according to the comments on the main figures and tables. Please remember that all figures and/or tables should be self-explanatory on a stand-alone basis.”

**Reply:** Thank you for this comment. We have now provided these throughout our Supplementary Tables.

**Point 39:** “Where website addresses are cited, when specifying the date of access, please use the word “accessed” instead of “cited” (e.g. [accessed: 10/04/2024]).”

**Reply:** Thank you for this comment. We have now amended the references accordingly.

**Point 40:** “Please ensure that journal name abbreviations match those found in the National Center for Biotechnology Information (NCBI) databases (https://eur03.safelinks.protection.outlook.com/?url=http%3A%2F%2Fwww.ncbi.nlm.nih.gov%2Fnlmcatalog%2Fjournals&data=05%7C02%7Ctf363%40universityofcambridgecloud.onmicrosoft.com%7C0ab3756e80ba42eab62608dc74be7a52%7C49a50445bdfa4b79ade3547b4f3986e9%7C1%7C0%7C638513608644475508%7CUnknown%7CTWFpbGZsb3d8eyJWIjoiMC4wLjAwMDAiLCJQIjoiV2luMzIiLCJBTiI6Ik1haWwiLCJXVCI6Mn0%3D%7C0%7C%7C%7C&sdata=WHJRvcqrqPC%2BZvqCYy0fKLP%2FeHNpDnswiIa48nazWIM%3D&reserved=0), and are appropriately formatted and capitalised. For example, “The Lancet Healthy Longevity” in reference [13] should be “Lancet Healthy Longev”.”

**Reply:** Thank you for this comment. We have now made sure that the journal name abbreviations match those in the National Center for Biotechnology Information databases.

**Point 41:** “To help us extend the reach of your research, please provide any X (formerly known as Twitter) handle(s) that would be appropriate to tag, including your own, your co-authors’, your institution, funder, or lab. Please enter in the submission form any handles you wish to be included when we post about this paper.”

**Reply:** Thank you for this comment. Please use these handles: @tmfmnk (Tomáš Formánek), @NUDZ_cz (National Institute of Mental Health), @psychiatry_ucam (Department of Psychiatry, University of Cambridge), @agentura_azvcr (Czech Health Research Council).

**Reviewer #1**

**Point 1:** “I appreciate the efforts made by the authors to address my and my fellow reviewers' constructive comments. Changes have improved the manuscript and increased its impact.”

**Reply:** Thank you once again for the assessment of our study.

**Reviewer #2**

**Point 1:** “We thank the authors for largely addressing our previous comments. The explanation on other medications/treatments might be indicated in the text, possibly as a limitation.”

**Reply:** Thank you once again for the assessment of our study. We have now provided this as a potential limitation as follows (page 12 line 560-561):

- “Eighth, we did not investigate the responses to medications and/or other treatment modalities following the infection itself in people with pre-existing mental disorders.”.

**Additional Changes**

**Change 1:** During our quality control, we noticed that we provided an incorrect end date for the first State of Emergency in Czechia. We have now corrected it as follows (page 6 line 212-214):

- “The first wave of the pandemic in Czechia lasted roughly from 1^st^ March 2020 to 30^th^ September 2020, with a State of Emergency being in place from 12^th^ March 2020 to 17^th^ May 2020. The first wave resulted in 70,968 incident infections [6].”.

We have now also provided information on a month-long third State of Emergency that we previously overlooked as follows (page 6 line 220-222):

- “Then, the 2021-2022 winter wave lasted from approximately 1^st^ November 2021 to 28^th^ February 2022, with a State of Emergency imposed from 26^th^ November to 25^th^ December, and led to 2,017,028 incident infections [6].”.

**Change 2:** During our quality control, we noticed that we provided one of the tables containing the E-values two times. We have now corrected this. Additionally, we have now provided the E-values also for affective disorders as follows (page 10 line 433-437):

- ”For models not consistent with a null-effect, the E-values ranged from 1.71 to 3.22 for substance use disorders, from 2.21 to 3.39 for psychotic disorders, from 1.50 to 1.88 for anxiety disorders, and from 1.45 to 1.55 for affective disorders. Higher E-values increase the confidence that the detected associations are not due to unaccounted for confounding. See details in Supplementary Table 15-16.”.

**References**

1.	Morris TP, Jarvis CI, Cragg W, Phillips PPJ, Choodari-Oskooei B, Sydes MR. Proposals on Kaplan–Meier plots in medical research and a survey of stakeholder views: KMunicate. BMJ Open. 2019;9(9):e030215. doi: 10.1136/bmjopen-2019-030215.

2.	Ministry of Health of the Czech Republic. COVID-19 Vaccination Strategy in Czech Republic. 2020.

3.	Kinkorová J, Topolčan O. Overview of healthcare system in the Czech Republic. EPMA Journal. 2012;3(1):4. doi: 10.1007/s13167-012-0139-9.

4.	Griffith GJ, Morris TT, Tudball MJ, Herbert A, Mancano G, Pike L, et al. Collider bias undermines our understanding of COVID-19 disease risk and severity. Nat Commun. 2020;11(1). doi: 10.1038/s41467-020-19478-2.

5.	Lin M, Lucas HC, Shmueli G. Too Big to Fail: Large Samples and the p-Value Problem. Inf Syst Res. 2013;24(4):906-17. doi: 10.1287/isre.2013.0480.

6.	Komenda M, Panoška P, Bulhart V, Žofka J, Brauner T, Hak J, et al. COVID-19: Overview of the Current Situation in Czechia Prague: Ministry of Health of the Czech Republic; 2020 [accessed: 10/04/2024]. Available from: <https://onemocneni-aktualne.mzcr.cz/covid-19>.
